# Applying High‐Dimensional Propensity Scores in a Study of Inhaled Corticosteroids and COVID‐19 Outcomes

**DOI:** 10.1002/pds.70248

**Published:** 2025-11-24

**Authors:** Marleen Bokern, John Tazare, Christopher T. Rentsch, Jennifer K. Quint, Ian J Douglas, Anna Schultze

**Affiliations:** ^1^ London School of Hygiene and Tropical Medicine London UK; ^2^ Faculty of Medicine National Heart & Lung Institute, Imperial College London London UK

**Keywords:** COVID‐19, high‐dimensional propensity scores, pharmacoepidemiology, residual confounding, respiratory epidemiology

## Abstract

**Background:**

In pharmacoepidemiologic studies of COVID‐19, there were concerns about bias from residual confounding. We investigated the effects of inhaled corticosteroids (ICS) on COVID‐19 outcomes, applying high‐dimensional propensity scores (HDPS) to adjust for unmeasured confounding.

**Methods:**

We selected patients with chronic obstructive pulmonary disease on 01 March 2020 from Clinical Practice Research Datalink (CPRD) Aurum, comparing ICS/LABA/(+/−LAMA) and LABA/LAMA users. ICS effects on the outcomes COVID‐19 hospitalisation and death were assessed through IPT‐weighted and unweighted Cox regression. HDPS were estimated from primary care observations, prescriptions and hospitalisations. SNOMED‐CT codes and dictionary of medicines and devices codes from CPRD Aurum were mapped to International Classification of Disease 10th revision codes and British National Formulary paragraphs, respectively. We estimated propensity scores (PS) combining prespecified and HDPS covariates, selecting the top 100, 250, 500, 750 and 1000 covariates ranked by confounding potential.

**Results:**

When excluding triple therapy users, conventional PS‐weighted estimates showed weak evidence of increased COVID‐19 hospitalisation risk among ICS users (HR 1.19 [95% CI: 0.92–1.54]). Results varied slightly based on the number of covariates included in HDPS (HR using 100 HDPS covariates excluding triple therapy 1.01 [95% CI: 0.76–1.33], HR using 250 HDPS covariates excluding triple therapy 1.24 [95% CI: 0.83–1.87]). Conventional PS‐weighted models showed weak evidence of a harmful association of ICS with COVID‐19 death when excluding triple therapy users (HR 1.24 [95% CI: 0.87–1.75]). HDPS‐weighting moved estimates toward the null (HR using 250 HDPS covariates excluding triple therapy 1.08 [95% CI: 0.73–1.59]).

**Conclusions:**

HDPS may have better controlled confounding for COVID‐19 deaths in this case. HDPS results can be sensitive to the number of covariates included, highlighting the importance of sensitivity analyses.

**Summary**

- Residual confounding, including residual confounding by indication, is a major concern in pharmacoepidemiologic studies of COVID‐19 outcomes.
- We apply high‐dimensional propensity scores (HDPS) to reduce residual confounding in a case study of inhaled corticosteroids (ICS) on COVID‐19 hospitalisation and death in CPRD Aurum.
- Conventional PS‐weighted analyses using only prespecified covariates suggested harmful effects of ICS on COVID‐19 hospitalisation and, to a lesser extent, deaths.
- HDPS weighted analyses of COVID‐19 hospitalisations were sensitive to the number of covariates included, with results moving towards the null for smaller number of covariates and away from the null when including more covariates, while for deaths, estimates moved towards the null consistently.
- HDPS demonstrated promise in addressing confounding even when comparison groups are suboptimal, but its performance depends on the careful selection and ranking of covariates.

## 1 Introduction

In non‐interventional research, it can be difficult to control for hard‐to‐measure concepts such as frailty or disease severity. Many methods exist to address concerns regarding residual confounding; for example, quantitative bias analysis (QBA) [1] and *e*‐values [2]. Most QBA methods rely on pre‐specifying the prevalence of and exposure/outcome association with a single confounder, while *e*‐values provide a measure of how strong an unmeasured confounder would need to be to explain away an observed association. High‐dimensional propensity scores (HDPS) aim to optimise confounding adjustment by considering recorded data as proxy variables [3].

Inhaled corticosteroids (ICS) are commonly used anti‐inflammatory therapies for COPD and were explored as a potential treatment for COVID‐19. Randomised controlled trials (RCTs) indicated that one inhaled ICS, budesonide, had a protective effect against severe COVID‐19 in patients with mild COVID‐19 [4, 5], but found no effect of ciclesonide or fluticasone [6, 7, 8]. Observational studies also examined the relationship between ICS and COVID‐19 outcomes, but findings were inconsistent and potentially influenced by biases including residual confounding [9, 10].

Given the inconsistent results obtained by observational studies, and the differences between trial results and observational study results on the question of ICS and COVID‐19 outcomes, we previously conducted an analysis investigating outcome misclassification in this clinical question and found that confounding was likely the main source of bias [11]. Therefore, we conducted this methodological extension to assess whether data‐driven confounding control methods could be used to improve inference from observational studies on this question.

We were particularly concerned about residual confounding from frailty and COPD severity (i.e., confounding by indication), which may not be fully captured in electronic health records (EHRs) by variables such as prior exacerbation history. These are difficult to measure directly but may be partially addressed through the use of proxy variables via HDPS. We aimed to compare conventional, investigator‐led propensity scores and HDPS when investigating the association between ICS and COVID‐19 hospitalisation and death in CPRD Aurum.

## 2 Methods

The study protocol was registered on ENCEPP EU PAS (register number 47885). This analysis was planned post hoc after results suggesting residual confounding may explain observed harmful associations [11].

The data sources, study population, and exposure, outcome and covariate definitions have previously been described [11], and are summarised below. The study design is depicted in Figure S1.

### 2.1 Data Source

This study used UK primary care data recorded in the Clinical Practice Research Datalink (CPRD) Aurum. CPRD Aurum includes data on 41 million patients (May 2022 build), with > 13 million patients currently registered (20% of UK population) [12] from > 1300 practices using EMIS management software [11]. CPRD Aurum is broadly representative of the English population [13].

CPRD Aurum was linked to Hospital Episode Statistics (HES) Admitted Patient Care (APC) and Office for National Statistics (ONS) Death Registry [13, 14]. HES APC holds information on all in‐patient contacts at NHS hospitals in England [14, 15]. The ONS death registry contains information on deaths occurring in England and Wales, including cause of death documented using International Classification of Disease 10th revision (ICD‐10) codes [15, 16]. Data were also linked to the Index of Multiple Deprivation (IMD), a postcode‐level indicator of socioeconomic status.

### 2.2 Study Population

We defined a cohort diagnosed with COPD before 1 March 2020 (index date) based on a validated algorithm [17]. Patients had to be alive, aged ≥ 35, and registered in CPRD Aurum on the index date. Patients needed to have 12 months' continuous registration and a record of current/former smoking before the index date. We excluded people with asthma recorded within 3 years before the index date, leukotriene receptor antagonist use within 4 months before the index date as this indicates asthma, or other chronic respiratory disease at any point before the index date. Patients were followed until the outcomes (as recorded in HES and ONS, respectively), death (recorded in ONS or CPRD), deregistration or 31 August 2020, whichever came first. The study period (1 March 2020–31 August 2020) approximated the time period between the start of the pandemic and the start of the second wave in the United Kingdom [18]. If death was registered in ONS, we used that date as the date of death. If death was missing in ONS but registered in CPRD, we used the date recorded in CPRD as the date of death.

#### 2.2.1 Exposure

Continuous treatment episodes were generated based on the recorded prescription issue date and information on intended duration, prescribed amount and dosage [11].

We used treatment episodes to categorise people as exposed to ICS (e.g., beclomethasone, budesonide)/long‐acting β‐agonist (LABA, e.g., formoterol, salmeterol) or LABA/long‐acting muscarinic antagonist (LAMA, e.g., tiotropium, umeclidinium) on the index date. We conducted two separate analyses. In the first, people using ICS + LABA + LAMA (i.e., triple therapy) were included in the ICS/LABA group. While this comparison is likely subject to strong confounding by indication as patients requiring triple therapy typically have more severe disease, this analysis assesses whether HDPS can address residual confounding in challenging settings with strong confounding. In the second, triple therapy users were excluded as we expected these patients to be sicker than those on dual therapy, creating a more suitable active comparator design. ICS/LABA was the exposure of interest and LABA/LAMA was the active comparator. We did not censor at the estimated discontinuation dates due to low discontinuation rates and concern that discontinuations would be inaccurately estimated due to pandemic‐related healthcare disruptions [19].

#### 2.2.2 Outcome

Outcomes were (i) hospitalisation with a primary diagnosis code for COVID‐19 in HES APC, and (ii) death with a diagnosis code for COVID‐19 as cause of death anywhere on the death certificate in the ONS Death Registry. COVID‐19 hospitalisation and death were identified with ICD‐10 codes U07.1 and U07.2.

### 2.3 Prespecified Covariates

The following covariates, selected based on input from a practising respiratory consultant clinician and based on previous studies [9] and defined on 1 March 2020, were included in propensity scores (PSs): age, gender, BMI (most recent within 10 years, categorised as underweight [< 18.5], normal [18.5–24.9], overweight [25–29.9] or obese [≥ 30]), smoking (current vs. former), ethnicity, cancer (ever), diabetes (ever), chronic kidney disease (ever), cardiovascular disease (ever), hypertension (ever), asthma (> 3 years ago), immunosuppression, influenza vaccination (past year), pneumococcal vaccination (past 5 years), IMD quintile and number of COPD exacerbations in the past 12 months as a marker of COPD severity, based on a validated algorithm [20]. A directed acyclic graph (DAG) depicting the assumed causal structure is in Figure S2.

### 2.4 Statistical Analyses

Cohort characteristics were summarised using descriptive statistics by exposure group. There were missing data for BMI (*n* = 550, 0.7%), ethnicity (*n* = 7785, 9.9%) and IMD (*n* = 42, 0.05%). People with missing BMI were assigned to the ‘normal’ BMI category, as we assumed individuals with extreme values were more likely to have this recorded in clinical practice, and therefore the small number of missing BMI values were likely to be closer to the ‘normal’ range [21]. Missing ethnicity was treated as a separate category [22, 23, 24], and patients with missing IMD were excluded as this may indicate poor quality records [25]. In a sensitivity analysis, we used multiple imputation to handle missing ethnicity values.

We estimated PSs and used inverse probability of treatment weighting (IPTW) to estimate the average treatment effect (ATE). Conventional PSs were estimated using logistic regression including the prespecified covariates listed above. Stabilised weights were calculated as $\frac{P\left(E=\mathrm{ICS}/\text{LABA}\right)}{\mathrm{PS}}\;\left(\mathrm{ICS}/\text{LABA}\right)$ and $\frac{P\left(E=\text{LABA}/\text{LAMA}\right)}{1-\mathrm{PS}}\;\left(\text{LABA}/\text{LAMA}\right)$, where the PS is the probability of receiving ICS. Overlap of the PSs across treatment groups was assessed graphically and by summarising PSs by treatment group. PSs were trimmed to the region of common support [26], that is, the PS range where individuals from both treatment groups are represented. This was applied for all analyses.

Cox regression was used to estimate hazard ratios (HRs) and 95% confidence intervals (CIs) for the association between exposure and outcomes. The proportional hazards assumption was assessed using Schoenfeld residuals. The Cox regression models were IPT‐weighted, and patients were censored at the outcomes, death, transfer out of the GP practice, or 31 August 2020 (end of study period).

We additionally calculate IPT‐weighted risk differences and survival curves, and present odds ratios estimated by logistic regression.

### 2.5 HDPS

When applying HDPS, data is separated into dimensions reflecting different care facets [3]. We separated available data into primary care observations (CPRD Aurum), prescription (CPRD Aurum) and hospitalisation (HES APC) information [27]. In CPRD Aurum, clinical observations are coded using SNOMED‐CT, while prescriptions are coded using dictionary of medicines and devices (dm + d) codes. Observations in HES are recorded using ICD‐10 codes. ICD‐10 codes are hierarchical, with the first three characters denoting clinical categories. To avoid sparsity, SNOMED‐CT codes were mapped to ICD‐10 using a mapping file supplied by the NHS, and subsequently truncated to three characters [28, 29]. dm + d codes were mapped to British National Formulary (BNF) paragraphs using a mapping file from the Bennett Institute [30]. For unmatched dm + d codes, the first six characters of the BNF chapter variable supplied in the CPRD code browser were used. Unmapped codes that had over 1 000 000 recordings in CPRD Aurum were mapped manually (*n* = 16). The top 100 unmapped codes for clinical observations and prescriptions are in Tables S1 and S2. BNF paragraphs correspond to drug classes or treatment indications rather than specific mechanisms of action (e.g., paragraph 030101 corresponds to bronchodilators, specifically adrenoceptor agonists) [27]. We excluded medications used to define the treatment groups (ICS, LABA and LAMA, as single‐ingredient or combined inhalers), and ICD‐10 codes used to define outcomes (U07) from the HDPS. We did not exclude codes used to define the prespecified covariates.

We assessed the occurrence of codes in the 12 months before the index date (1 March 2020). The original algorithm applies a prevalence filter which includes the top codes ranked based on marginal prevalence [3]. In line with Schuster et al., we did not apply this filter as variables with a low marginal prevalence can still be important confounders [31].

For each patient, we generated variables indicating if a code was recorded at least once (≥ 1), sporadically (≥ median) or frequently (≥ 75%‐ile) in the year before the index date, relative to the number of occurrences of the relevant code per patient among those who had ≥ 1 occurrence of the code. For the primary care observation dimension, we considered codes that were ever‐present before 1 March 2020, reflecting the fact that conditions, particularly chronic ones, may not be recorded at every consultation. Consistent with other applications of HDPS in CPRD, this information was included by redefining ‘once’ variables for this dimension [27].

Covariates were prioritised using the Bross formula, which uses covariate prevalence in exposed (P_C1_) and unexposed (P_C0_), and the relative risk between confounder and outcome (RR_CD_) to rank covariates by their potential to bias the exposure–outcome association [32].

We excluded variables that behaved like instrumental variables, that is, strongly associated with exposure but not outcomes; defined using cut‐offs based on the relative risk between confounder and exposure (RR_CE_) and the RR_CD_ (|log(RR_CE_)| > 1.1 and |log(RR_CD_)| < 0.5) [29].

Covariates identified using HDPS were included in logistic regression models alongside predefined covariates to estimate PSs. Subsequently, IPTWs were used to weight logistic and Cox regression models to estimate the impact of ICS on the outcomes. We calculated standardised mean differences (SMDs) in covariate distributions between treatment groups before PS weighting, after conventional PS weighting using prespecified covariates, and after HDPS weighting.

We varied the number of covariates included in the HDPS (100, 250, 500, 750, 1000), with no predefined number specified. The top 30 ranked covariates for each cohort and outcome are available in Tables S3–S6. Post hoc, we conducted an analysis where covariates ranked 1st–250th were added one by one to assess the sensitivity of results to the inclusion of particular covariates.

A summary of key features and decisions of the HDPS approach is available in Table S7 [29].

Data were managed using Stata version 17.0 [33] and analysis carried out using R (version 4.3.3) [34]. Code lists and data management and analysis code are on GitHub (https://github.com/bokern/ics_hdps).

## 3 Results

Cohort characteristics have previously been described [11]. We included 56 029 patients using ICS/LABA (14 905 excluding triple therapy users) and 22 307 patients using LABA/LAMA at baseline (Table 1).

**TABLE 1:** Baseline cohort characteristics.

	Including triple therapy users						Excluding triple therapy users					
			Standardised mean differences						Standardised mean differences			
	LABA/LAMA (*n* = 22 307)	ICS/LABA (*n* = 56 029)	Unweighted	Weighted conventional PS	250 HDPS covariates (hospitalisation)	250 HDPS covariates (death)	LABA/LAMA (*n* = 22 307)	ICS/LABA (*n* = 14 905)	Unweighted	Weighted conventional PS	250 HDPS covariates (hospitalisation)	250 HDPS covariates (death)
Gender = female	10 068 (45.1)	26 217 (46.8)	0.033	0.004	0.002	0.001	10 068 (45.1)	7089 (47.6)	0.049	0.001	< 0.001	0.001
Age, mean (SD)	70.83 (10.23)	71.32 (10.47)	0.048	0.002	0.01	0.007	70.83 (10.23)	71.38 (11.29)	0.051	0.002	0.002	0.002
IMD			0.045	0.004	0.005	0.007			0.027	0.002	0.002	0.002
1	3048 (13.7)	7178 (12.8)					3048 (13.7)	2021 (13.6)				
2	3813 (17.1)	9213 (16.4)					3813 (17.1)	2562 (17.2)				
3	4077 (18.3)	9971 (17.8)					4077 (18.3)	2759 (18.5)				
4	5012 (22.5)	12 722 (22.7)					5012 (22.5)	3473 (23.3)				
5	6357 (28.5)	16 945 (30.2)					6357 (28.5)	4090 (27.4)				
Diabetes	5515 (24.7)	14 074 (25.1)	0.009	0.003	0.01	0.011	5515 (24.7)	3684 (24.7)	< 0.001	0.001	< 0.001	0.001
Hypertension	11 312 (50.7)	28 592 (51.0)	0.006	0.003	0.002	0.006	11 312 (50.7)	7689 (51.6)	0.018	0.001	< 0.001	< 0.001
Cardiovascular disease	6538 (29.3)	16 766 (29.9)	0.013	0.002	0.003	0.005	6538 (29.3)	4387 (29.4)	0.003	< 0.001	< 0.001	< 0.001
Cancer	4413 (19.8)	10 539 (18.8)	0.025	0.001	0.005	0.008	4413 (19.8)	2827 (19.0)	0.021	0.001	0.001	0.001
Past asthma	2664 (11.9)	15 339 (27.4)	0.396	0.002	0.022	0.011	2664 (11.9)	4134 (27.7)	0.404	0.001	0.002	0.002
Kidney impairment	6737 (30.2)	16 719 (29.8)	0.008	0.007	0.012	0.012	6737 (30.2)	4567 (30.6)	0.01	0.001	0.002	0.001
Immunosuppression	274 (1.2)	664 (1.2)	0.004	< 0.001	0.003	0.004	274 (1.2)	188 (1.3)	0.003	< 0.001	< 0.001	< 0.001
Influenza vaccine	17 955 (80.5)	44 884 (80.1)	0.01	0.001	< 0.001	0.002	17 955 (80.5)	11 389 (76.4)	0.099	< 0.001	< 0.001	< 0.001
Pneumococcal vaccine	3236 (14.5)	5983 (10.7)	0.116	0.001	0.001	< 0.001	3236 (14.5)	1497 (10.0)	0.136	0.001	< 0.001	0.001
COPD exacerbation in past 12 months	6221 (27.9)	22 793 (40.7)	0.272	0.002	0.005	0.001	6221 (27.9)	4638 (31.1)	0.071	0.001	< 0.001	0.001
Former smoking	12 239 (54.9)	33 280 (59.4)	0.092	0.002	0.01	0.008	12 239 (54.9)	8942 (60.0)	0.104	0.001	0.001	0.001
Ethnicity (%)			0.051	0.009	0.015	0.009			0.101	0.002	0.004	0.004
White	19 574 (87.7)	49 366 (88.1)					19 574 (87.7)	12 890 (86.5)				
South Asian	197 (0.9)	742 (1.3)					197 (0.9)	292 (2.0)				
Black	130 (0.6)	351 (0.6)					130 (0.6)	138 (0.9)				
Mixed	49 (0.2)	142 (0.3)					49 (0.2)	44 (0.3)				
Unknown	2357 (10.6)	5428 (9.7)					2357 (10.6)	1541 (10.3)				
BMI (%)			0.07	0.004	0.009	0.009			0.038	0.001	0.002	0.002
Underweight (< 18.5)	969 (4.3)	3138 (5.6)					969 (4.3)	619 (4.2)				
Normal (18.5–24.9)	6922 (31.0)	18 153 (32.4)					6922 (31.0)	4629 (31.1)				
Overweight (25–29.9)	7167 (32.1)	17 339 (30.9)					7167 (32.1)	5020 (33.7)				
Obese (≥ 30)	7249 (32.5)	17 399 (31.1)					7249 (32.5)	4637 (31.1)				

### 3.1 Propensity Score Diagnostics

**FIGURE 1:**
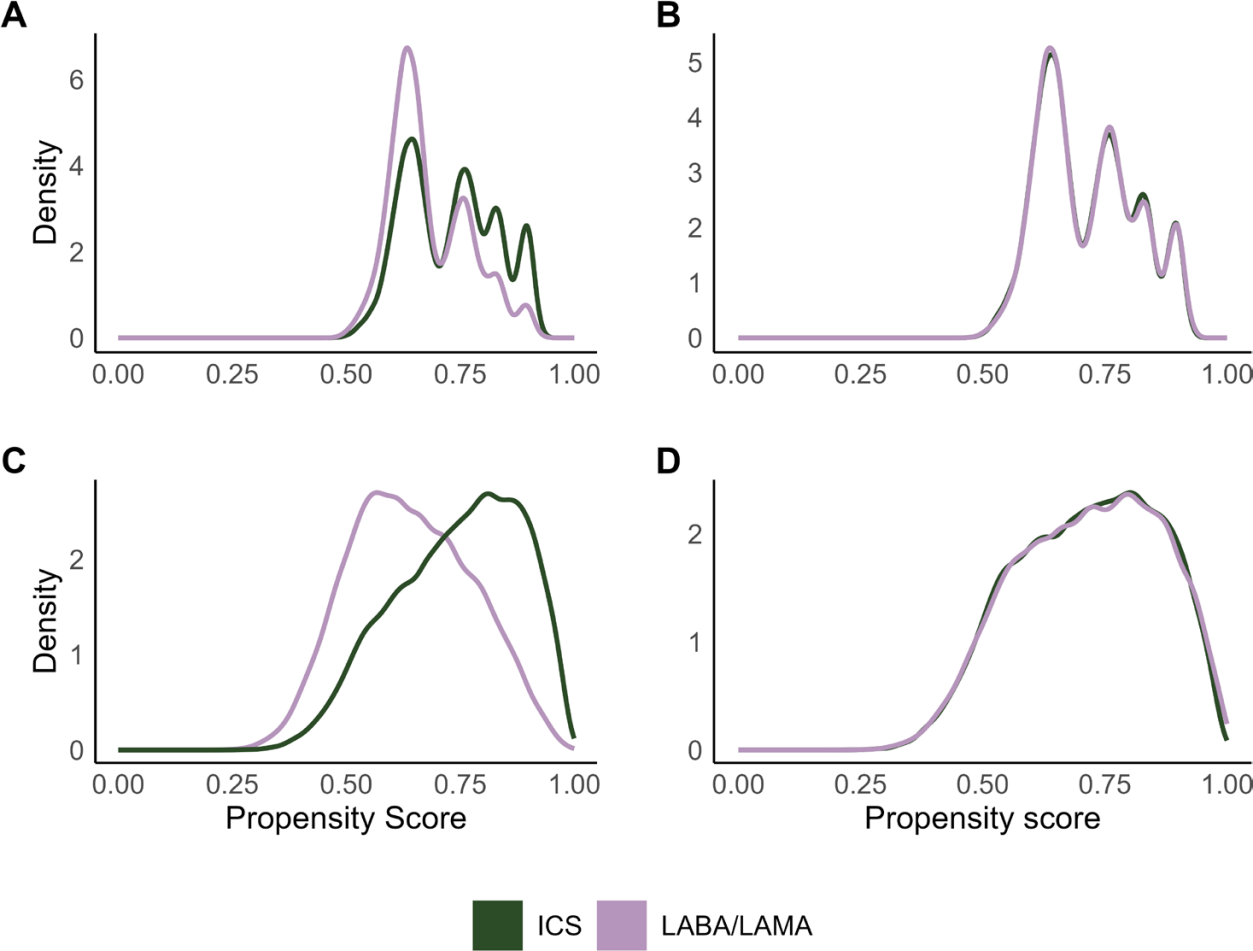
Density distributions of propensity scores for ICS and LABA/LAMA treatment groups before and after weighting for the outcome COVID‐19 hospitalisation, excluding triple therapy users. (A) Unweighted propensity score distributions using predefined covariates, (B) weighted propensity score distribution, (C) high‐dimensional propensity score (HDPS) distribution for 250 covariates for the outcome COVID‐19 hospitalisation and (D) weighted HDPS distribution for 250 covariates.

**FIGURE 2:**
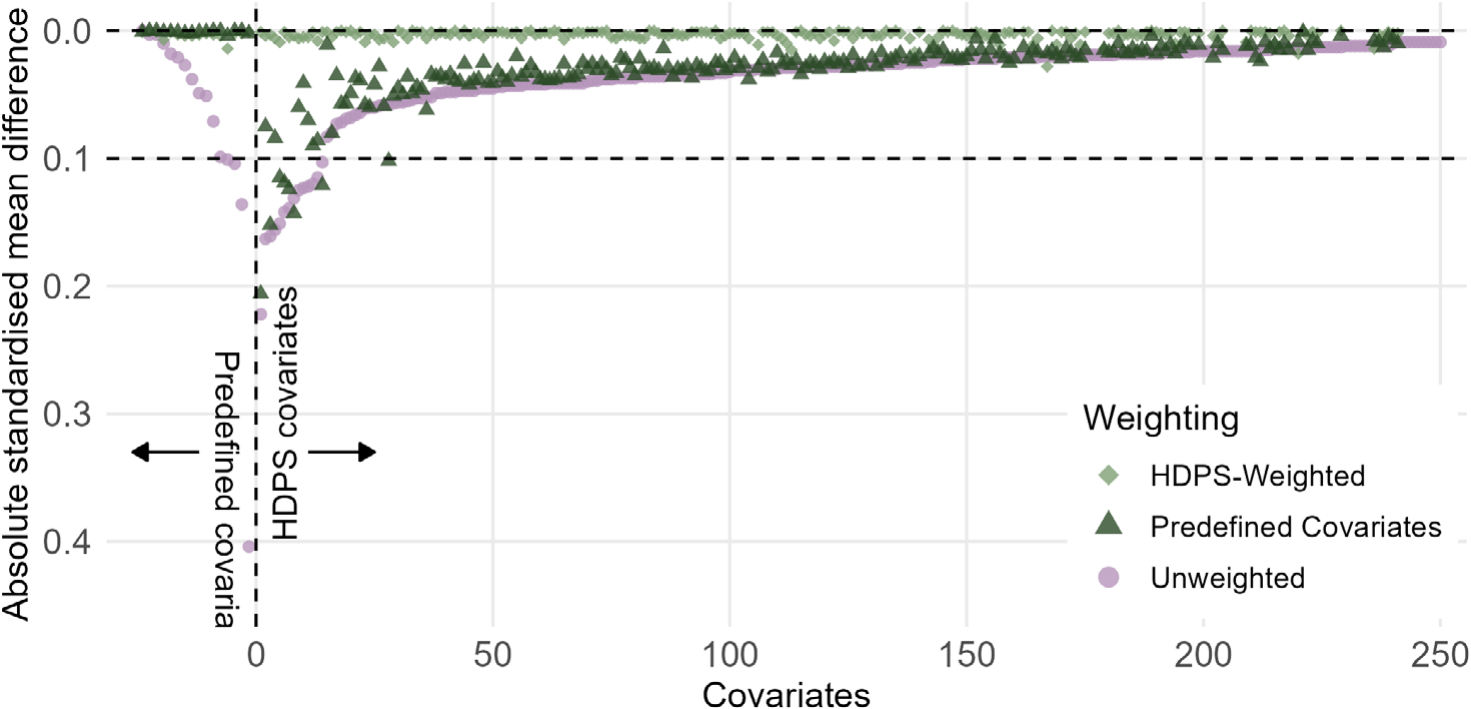
Comparison of absolute standardised differences in the predefined and top 250 high‐dimensional propensity score covariates between unweighted, predefined and HDPS weighted samples for COVID‐19 hospitalisations, excluding triple therapy users.

Figure 1 shows unweighted and weighted PS distributions for the analysis using 250 HDPS covariates for COVID‐19 hospitalisations, excluding triple therapy users. For all analyses, trimming PSs to the region of common support did not exclude many patients (< 1%, Table S10). Prespecified covariates were balanced after weighting (SMD < 0.1) using the conventional PS. Covariate balance was better for the predefined covariates when estimating the PS using only those covariates. However, weighting using HDPS achieved adequate balance (SMD < 0.1) of predefined covariates while achieving better balance for the additional HDPS covariates (Figure 2 and Table 1). For illustration, we present diagnostics for 250 HDPS covariates for COVID‐19 hospitalisations excluding triple therapy users below (Figure 2 and Table 1). For COVID‐19 hospitalisations excluding triple therapy users, the top 250 codes included 145 (58.0%) from hospital data, 82 primary care prescription (32.8%) and 23 primary care observation codes (9.2%) (Figure S9). Diagnostics for other implementations of the HDPS showed similar results and are in the Supporting Information.

### 3.2 Effects of ICS on COVID‐19 Hospitalisations

**FIGURE 3:**
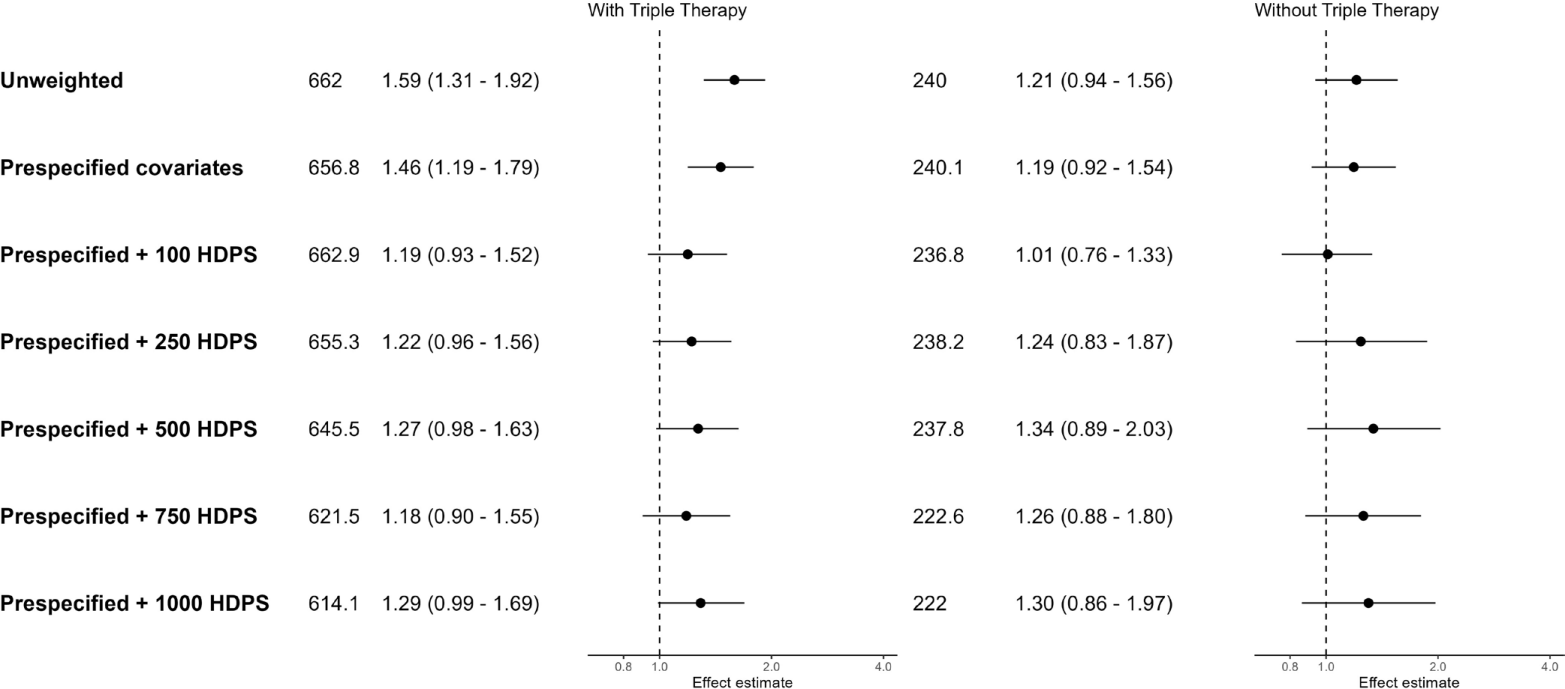
Forest plot of hazard ratios and 95% confidence intervals for COVID‐19 hospitalisations, comparing ICS/LABA (+/−LAMA) users to LABA/LAMA users. Effect estimates > 1 indicate an increased risk in the ICS group.

Conventional PS‐weighted analyses including triple therapy users suggested an increased risk of COVID‐19 hospitalisation among ICS users (HR 1.46 [95% CI: 1.19–1.79]), which was attenuated when excluding triple therapy users (HR 1.19 [95% CI: 0.92–1.54]). All HDPS applications moved effect estimates towards null (Figure 3). Point estimates varied slightly depending on the number of included covariates (HR using 100 HDPS‐covariates including triple therapy users 1.19 [95% CI: 0.93–1.52], HR using 250 HDPS‐covariates including triple therapy users 1.22 [95% CI: 0.96–1.56]). When excluding triple therapy users, results were more sensitive to the number of covariates included in the HDPS: The HDPS calculated using the top 100 covariates gave an almost exact null result (HR 1.01 [95% CI: 0.76–1.33]). Including more covariates shifted the effect estimate away from null (HR using 250 HDPS‐covariates 1.24 [95% CI: 0.83–1.87]), although CIs were wide and remained consistent with null effects.

**FIGURE 4:**
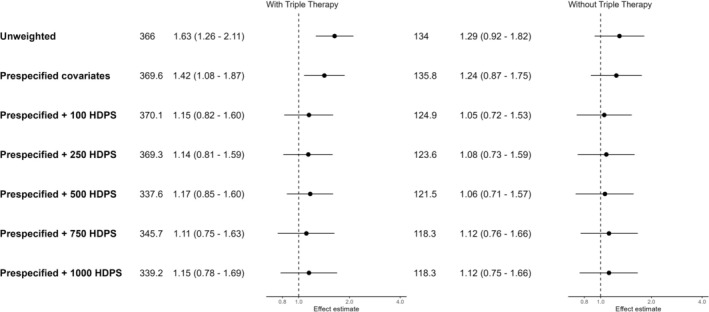
Forest plot of hazard ratios and 95% confidence intervals for COVID‐19 deaths, comparing ICS/LABA (+/−LAMA) users to LABA/LAMA users. Effect estimates > 1 indicate an increased risk in the ICS group.

### 3.3 Effects of ICS on COVID‐19 Death

Conventional PS‐weighted models indicated a harmful association of ICS when including triple therapy users (HR 1.42 [95% CI: 1.08–1.87]) and very weak evidence of a harmful association (HR 1.24 [95% CI: 0.87–1.75]) when excluding triple therapy users. Using HDPS shifted estimates towards the null (HR using 100 HDPS covariates including triple therapy users 1.15 [95% CI: 0.82–1.60], excluding triple therapy users 1.05 [95% CI: 0.72–1.53]) (Figure 4). For both exposure definitions, the CIs for all applications of HDPS crossed the null and were insensitive to the number of covariates. With more covariates, the estimates for the cohort including triple therapy users became very close to those in the cohort excluding triple therapy users (HR using 1000 HDPS covariates including triple therapy 1.15 [95% CI: 0.78–1.69], excluding triple therapy 1.12 [95% CI: 0.75–1.66]).

**FIGURE 5:**
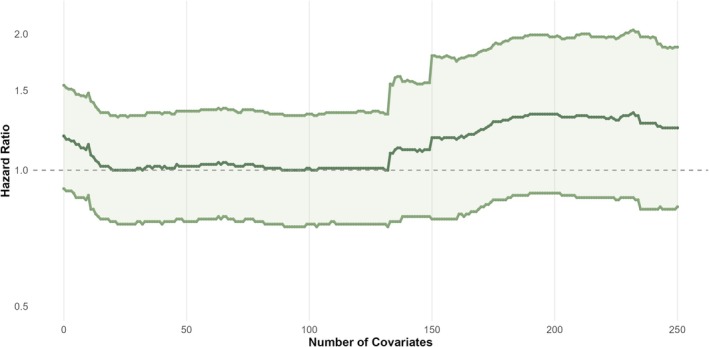
Sensitivity analyses assessing the impact of the number of high‐dimensional propensity score covariates selected on the effect estimate, adding the top 250 Bross‐ranked covariates one by one. Propensity scores were estimated using logistic regression, and treatment effects were estimated using an inverse probability of treatment weighted Cox regression model. The outcome was COVID‐19 hospitalisation, excluding triple therapy users.

### 3.4 Sensitivity Analyses

Adding covariates one by one showed that certain covariates made substantial changes in the effect estimates (Figure 5). The largest changes occurred when individuals with rare combinations of covariates received a large weight and experienced the outcome. These combinations of covariates cannot be disclosed for data privacy reasons.

For both outcomes, point estimates were identical when using Cox and logistic regression models. However, CIs were consistently narrower when using logistic regression (Figures S41 and S42). Using multiple imputation to handle missing ethnicity values did not substantially change results (Table S11). Risk differences were generally small in all analyses, and the comparative pattern of estimates matched that seen in the main analyses (Figures S46–S52).

## 4 Discussion

Using conventional IPT‐weighted models, we observed an increased risk of COVID‐19 hospitalisation and death among ICS users when including triple therapy users, but no evidence of increased risk when excluding triple therapy users. For COVID‐19 hospitalisations, results were sensitive to the number of covariates included in HDPS, with the point estimate moving closer to the null or further away depending on the exposure definition used. For COVID‐19 deaths, HDPS consistently moved the point estimate closer to the null, with no evidence against the null hypothesis. The analyses including and excluding triple therapy users gave very similar results after HDPS weighting. This is the first study to implement HDPS in CPRD Aurum, building upon previous work to implement HDPS in CPRD GOLD [27].

### 4.1 Comparison to Previous Studies

Many studies have applied HDPS in CPRD GOLD, with most using 500 covariates to estimate HDPS [35, 36, 37, 38, 39, 40, 41, 42, 43, 44, 45], although some used as few as 40 [46, 47]. Most present results applying HDPS, although one presented results comparing conventional PS‐weighting to HDPS‐weighting, finding superior confounding control with HDPS [38]. Another study compared HDPS‐decile adjusted results to results adjusted for prespecified covariates only and found similar effect estimates in the two analyses [44].

No pharmacoepidemiologic study applied HDPS to UK EHR data during the COVID‐19 pandemic. However, several studies investigated the same clinical question. A cohort study using the EHR platform OpenSAFELY [9] found an aHR for COVID‐19 death of 1.39 (1.10–1.76) among patients with COPD. Using *e*‐values, the authors found that an unmeasured confounder would need to be associated with either exposure or outcome by a risk ratio of at least 1.43 to explain the association on its own.

A QResearch study [10] found a more modest risk of severe COVID‐19 and COVID‐19 death associated with ICS use (COVID‐19 hospitalisation aHR 1.13 [1.03–1.23], COVID‐19 death aHR 1.15 [1.01–1.31]). Compared with our study, this study included more prespecified confounders. Our results after HDPS are very similar to the fully adjusted results from this study.

### 4.2 Interpretation

For both cohorts and outcomes, the highest‐ranked empirically identified covariates included codes relating to nicotine dependence and lung disease (e.g., J44 [Other chronic obstructive pulmonary disease], J42 [Unspecified chronic bronchitis], J18 [pneumonia, organism unspecified], E94 [bronchial tests], J45 [asthma], E97 [respiratory education], J96 [respiratory failure, not otherwise specified], Tables S3–S6). This suggests that variables related to respiratory disease were potentially important confounders despite our efforts to mitigate confounding by indication through adjustment for prior exacerbations, past asthma diagnosis, and exclusion of patients with current asthma or other chronic respiratory disease. The identification of these additional variables by HDPS suggests that it may have provided better adjustment for this than conventional PS‐weighting, and that our predefined covariates and exclusion criteria could have been strengthened by incorporating a broader set of respiratory‐related codes. Compared to other approaches such as *e*‐values or QBA, HDPS offers the advantage of not having to specify a single confounder and its strength of association with the exposure and outcome, and may adjust for multiple hard‐to‐measure concepts, provided suitable proxies are captured in the data. HDPS can also be used in conjunction with QBA or *e*‐values, which may aid in the quantification of unmeasured confounding.

For hospitalisations, results were sensitive to the number of HDPS covariates included, illustrating the importance of varying the number of covariates in sensitivity analyses. A sensitivity analysis that included covariates one by one showed that even low‐ranked covariates can result in substantial changes in the effect estimates if patients with unusual combinations of covariates have the outcome, especially if the outcome is rare. It is possible that hospitalisation is a paradoxical outcome, with a selection process occurring regarding which patients are admitted to hospital with COVID‐19. We assumed patients in the ICS group were sicker and would have more severe COVID‐19 compared to those using LABA/LAMA, and therefore better control for confounding by disease severity would move effect estimates towards the null. However, it is possible that for COVID‐19 hospitalisations, confounding operated in both directions, with sicker or frail patients less likely to be hospitalised if they were deemed unlikely to survive [48]. The extent of this selection process may have varied over time depending on changes in hospital pressures or triaging processes. Similar issues were identified when using intensive care unit admission as an outcome [48]. ONS data on place of death indicates that during our study period around 28% of COVID‐19 deaths occurred outside hospitals [49], suggesting that not everyone with severe COVID‐19 was admitted to hospital and there may have been selection into hospitals. Therefore, COVID‐19 hospitalisation may not have been a suitable outcome to represent severe COVID‐19 if those with the most severe COVID‐19 died without hospitalisation.

For deaths, point estimates for the two treatment cohorts were similar. Inclusion of the 100 highest‐ranked covariates in the HDPS shifted the effect estimate towards null, while inclusion of additional covariates did not substantially change effect estimates. With inclusion of more covariates, effect estimates in the cohorts including and excluding triple therapy users became more similar. The cohort including triple therapy had more precision due to larger sample size. In the United Kingdom, LABA/LAMA and ICS/LABA are commonly used treatment combinations for COPD. Treatment should be stepped up to triple therapy if COPD remains uncontrolled. We therefore expect patients using triple therapy to have more severe COPD and be sicker than patients on dual therapy. As a result, excluding triple therapy users from the ICS/LABA group should provide a better comparison to the LABA/LAMA group. Taken together, this indicates that HDPS may offer better confounding control than conventional PS‐weighting in this case study.

### 4.3 Strengths and Limitations

Strengths of this study include the size and representativeness of CPRD Aurum and comprehensive capture of hospitalisations and deaths. The number of covariates included in our models remained below the commonly cited 1:10 covariate‐to‐event ratio threshold, reducing the risk of model overfitting [50]. We focused on a time period at the beginning of the pandemic, before vaccines were available and before different SARS‐CoV‐2 variants arose. This early period also reflects a time when dexamethasone was not yet the standard of care for hospitalised COVID‐19 patients. In a previous analysis [11], we established that outcome misclassification likely did not substantially impact the effect estimates in this study.

Due to short follow‐up, we could not use a new‐user design. Hence, the patients included in this study are likely heterogeneous in terms of disease stage and treatment history, and we assumed any effect of ICS on the outcomes was independent of treatment history and length. The time‐fixed exposure could have introduced some misclassification, but based on evidence of stockpiling behaviour in the early pandemic [19], it may have been difficult to accurately determine treatment discontinuation during this time. Additionally, we did not have information on in‐hospital prescriptions.

We observed minor deviations from proportional hazards, but graphical inspection indicated this was likely due to low numbers of outcomes and the characteristics of people with severe COVID‐19 changing throughout the study period. The HRs presented should be interpreted as an average over the time period.

The Bross formula used to prioristise variables for HDPS is suited to binary covariates, exposures and outcomes only. Continuous confounders such as laboratory or diagnostic test results do not naturally get considered in the HDPS. Therefore, if these factors are important confounders, they will not directly be accounted for. Additionally, by treating outcomes as binary, the algorithm disregards information about follow‐up time. However, censoring was low in this cohort, with logistic and Cox regression giving almost identical results. Factors that are not recorded in the used data sources may still confound the effect estimates. In this cohort, codes relating to forced expiratory volume in 1 s measurements or Medical Research Council Dyspnoea Scale may have additionally controlled for confounding by COPD severity [51]. Therefore, HDPS may not completely eliminate residual confounding.

Including many variables in the HDPS carries the risk of conditioning on IVs, mediators or colliders. To minimise overadjustment, all covariates were measured before the index date, and no time‐varying covariates were included. The Bross formula downweights variables that empirically act as IVs and we additionally implemented cutoffs to exclude covariates behaving like IVs [52]. Despite such precautions, the inclusion of inappropriate variables may introduce some bias. However, prior empirical and simulation studies have shown that the HDPS generally improves confounding control even at the cost of small increases in bias from irrelevant variables, particularly when those variables are data‐driven and prioritised based on their potential for confounding [3, 53]. Overall, the net effect typically favours improved confounder adjustment rather than harm from overadjustment.

## 5 Conclusions

The results provide weak evidence of an association between ICS and increased risk of COVID‐19 hospitalisation, but no effect on COVID‐19 death. The analysis of deaths suggests that HDPS may have achieved better confounding control than conventional PS weighting in this case study. In cases where the confounding structure is unclear, such as for the outcome COVID‐19 hospitalisations, using a data‐driven approach such as HDPS can lead to inconsistent results that may be sensitive to the inclusion of even low‐ranked covariates, highlighting the importance of sensitivity analyses.

### 5.1 Plain Language Summary

A key challenge when researching the effects of medications using EHRs is accounting for the fact that people who receive different medications often differ in important ways. Such differences, called confounding, are typically accounted for using statistical methods which require researchers to pre‐specify all important confounders. A newer method, called HDPS, uses a data‐driven approach to select what confounders to account for instead. These methods have not yet been applied to studies of ICS and COVID‐19 outcomes, an area where studies have found conflicting findings. We used EHRs from the United Kingdom to compare the risk of COVID‐19 hospitalisation and death among patients with chronic obstructive pulmonary disease taking two different treatments (ICS/LABA and LABA/LAMA) using both conventional and HDPS methods. Our findings showed that HDPS can reduce important differences between patients (confounding), but that the results can be sensitive to the number of covariates included. This demonstrates the value of HDPS and the need for researchers to run their analysis using several different assumptions.

## Author Contributions

M.B., J.T., A.S., C.T.R., I.J.D. contributed to the study design. M.B. conducted the data management and analysis and drafted the manuscript.

All authors contributed to the reviewing and editing of the manuscript. All authors were involved in the design and conceptual development and reviewed and approved the final manuscript.

## Funding

John Tazare was funded by the Wellcome Trust (224485/Z/21/Z).

## Ethics Statement

The study was approved by the London School of Hygiene and Tropical Medicine Research Ethics Committee (Reference: 27896) and the Independent Scientific Advisory Committee of the United Kingdom Medicines and Healthcare Products Regulatory Agency (Approval Number: 22_001876).

## Conflicts of Interest

M.B. is funded by a GSK PhD studentship to investigate the application of quantitative bias analysis in observational studies of COVID‐19. I.J.D. has unrestricted grants from and shares in GSK. AS is employed by LSHTM on a fellowship funded by GSK. The other authors declare no conflicts of interest.

## Supporting information


**Data S1:** pds70248‐sup‐0001‐Supinfo.pdf.
