# Supplementary material for: Applying High‐Dimensional Propensity Scores in a Study of Inhaled Corticosteroids and COVID‐19 Outcomes
Source: Pharmacoepidemiol Drug Saf. 2025 Nov 24;34(12):e70248. doi: 10.1002/pds.70248 (PMC12644305; doi:10.1002/pds.70248)
Supplement: Supplementary file 1 — Data S1: pds70248‐sup‐0001‐Supinfo.pdf. [file PDS-34-e70248-s001.pdf]

Supplementary material: Applying high-dimensional propensity scores in a study of inhaled corticosteroids and COVID-19 outcomes

Marleen Bokern[a*], John Tazare[a], Christopher T. Rentsch[a], Jennifer K. Quint[b], Ian Douglas[a], Anna Schultze[a]

*Corresponding author; email: marleen.bokern@lshtm.ac.uk

[a] London School of Hygiene and Tropical Medicine, Keppel Street, London WC1E 7HT, UK

[b] Faculty of Medicine, National Heart & Lung Institute, Imperial College London, London, UK

1. *Supplementary tables* ... 4

Supplementary Table 1 100 most frequent unmatched SNOMED-CT codes. ... 4
Supplementary Table 2 Top 100 unmapped product codes ... 6
Supplementary Table 3 Bias Information for COVID-19 hospitalisations, with triple therapy users. ... 8
Supplementary Table 4 Bias Information for COVID-19 hospitalisations, without triple therapy users ... 9
Supplementary Table 5 Bias Information for COVID-19 deaths, with triple therapy users ... 10
Supplementary Table 6 Bias Information for COVID-19 deaths, without triple therapy users ... 11
Supplementary Table 7 Summary of decisions related to the implementation of HDPS ... 12
Supplementary Table 9 Baseline characteristics (No triple therapy users) ... 16
Supplementary Table 10 Summary of weights before and after trimming the propensity scores to the region of common support ... 18
Supplementary Table 11 Results of Cox regression adjusted using prespecified covariates, with missing ethnicity values handled via multiple imputation ... 19

2. *Supplementary figures* ... 20

Supplementary Figure 1 Study diagram ... 20
Supplementary Figure 2 Directed Acyclic Graph (DAG) depicting the assumed causal relationships underlying the analysis of inhaled corticosteroid (ICS) use and COVID-19 outcomes. ... 20

2.1. Concept plots for outcome COVID-19 hospitalisation, including triple therapy users ... 21

Supplementary Figure 3 Summary of high-level concepts captured in the top 100 ranked high-dimensional propensity score covariates by data dimension for COVID-19 hospitalisations, including triple therapy users ... 22
Supplementary Figure 4 Summary of high-level concepts captured in the top 250 ranked high-dimensional propensity score covariates by data dimension for COVID-19 hospitalisations, including triple therapy users ... 23
Supplementary Figure 5 Summary of high-level concepts captured in the top 500 ranked high-dimensional propensity score covariates by data dimension for COVID-19 hospitalisations, including triple therapy users ... 24
Supplementary Figure 6 Summary of high-level concepts captured in the top 750 ranked high-dimensional propensity score covariates by data dimension for COVID-19 hospitalisations, including triple therapy users ... 25
Supplementary Figure 7 Summary of high-level concepts captured in the top 1000 ranked high-dimensional propensity score covariates by data dimension for COVID-19 hospitalisations, including triple therapy users ... 26

2.2. Concept plots for outcome COVID-19 hospitalisation, excluding triple therapy users 27
Supplementary Figure 8 Summary of high-level concepts captured in the top 100 ranked high-dimensional propensity score covariates by data dimension for COVID-19 hospitalisations, excluding triple therapy users 27
Supplementary Figure 9 Summary of high-level concepts captured in the top 250 ranked high-dimensional propensity score covariates by data dimension for COVID-19 hospitalisations, excluding triple therapy users 28
Supplementary Figure 10 Summary of high-level concepts captured in the top 500 ranked high-dimensional propensity score covariates by data dimension for COVID-19 hospitalisations, excluding triple therapy users 29
Supplementary Figure 11 Summary of high-level concepts captured in the top 750 ranked high-dimensional propensity score covariates by data dimension for COVID-19 hospitalisations, excluding triple therapy users 30
Supplementary Figure 12 Summary of high-level concepts captured in the top 1000 ranked high-dimensional propensity score covariates by data dimension for COVID-19 hospitalisations, excluding triple therapy users 31
2.3. Concept plots for outcome COVID-19 death, including triple therapy users 32
Supplementary Figure 13 Summary of high-level concepts captured in the top 100 ranked high-dimensional propensity score covariates by data dimension for COVID-19 death, including triple therapy users 32
Supplementary Figure 14 Summary of high-level concepts captured in the top 250 ranked high-dimensional propensity score covariates by data dimension for COVID-19 death, including triple therapy users 33
Supplementary Figure 15 Summary of high-level concepts captured in the top 500 ranked high-dimensional propensity score covariates by data dimension for COVID-19 death, including triple therapy users 34
Supplementary Figure 16 Summary of high-level concepts captured in the top 750 ranked high-dimensional propensity score covariates by data dimension for COVID-19 death, including triple therapy users 35
Supplementary Figure 17 Summary of high-level concepts captured in the top 1000 ranked high-dimensional propensity score covariates by data dimension for COVID-19 death, including triple therapy users 36
2.4. Concept plots for outcome COVID-19 death, excluding triple therapy users 37
Supplementary Figure 18 Summary of high-level concepts captured in the top 100 ranked high-dimensional propensity score covariates by data dimension for COVID-19 death, excluding triple therapy users 37
Supplementary Figure 19 Summary of high-level concepts captured in the top 250 ranked high-dimensional propensity score covariates by data dimension for COVID-19 death, excluding triple therapy users 38
Supplementary Figure 20 Summary of high-level concepts captured in the top 500 ranked high-dimensional propensity score covariates by data dimension for COVID-19 death, including triple therapy users 39
Supplementary Figure 21 Summary of high-level concepts captured in the top 750 ranked high-dimensional propensity score covariates by data dimension for COVID-19 death, excluding triple therapy users 40
Supplementary Figure 22 Summary of high-level concepts captured in the top 1000 ranked high-dimensional propensity score covariates by data dimension for COVID-19 death, excluding triple therapy users 41

2.5. **Diagnostic plots for HDPS..42**
Supplementary Figure 23 Diagnostic plots for high-dimensional propensity score weighted analysis for COVID-19 hospitalisations, including triple therapy users, including the top 100 ranked covariates. 43
Supplementary Figure 24 Diagnostic plots for high-dimensional propensity score weighted analysis for COVID-19 hospitalisations, including triple therapy users, including the top 500 ranked covariates. 44
Supplementary Figure 25 Diagnostic plots for high-dimensional propensity score weighted analysis for COVID-19 hospitalisations, including triple therapy users, including the top 750 ranked covariates. 45
Supplementary Figure 26 Diagnostic plots for high-dimensional propensity score weighted analysis for COVID-19 hospitalisations, including triple therapy users, including the top 1000 ranked covariates. 46
Supplementary Figure 27 Diagnostic plots for high-dimensional propensity score weighted analysis for COVID-19 hospitalisations, excluding triple therapy users, including the top 100 ranked covariates.........47
Supplementary Figure 28 Diagnostic plots for high-dimensional propensity score weighted analysis for COVID-19 hospitalisations, excluding triple therapy users, including the top 250 ranked covariates.........48
Supplementary Figure 29 Diagnostic plots for high-dimensional propensity score weighted analysis for COVID-19 hospitalisations, excluding triple therapy users, including the top 500 ranked covariates.........49
Supplementary Figure 30 Diagnostic plots for high-dimensional propensity score weighted analysis for COVID-19 hospitalisations, excluding triple therapy users, including the top 750 ranked covariates.........50
Supplementary Figure 31 Diagnostic plots for high-dimensional propensity score weighted analysis for COVID-19 hospitalisations, excluding triple therapy users, including the top 1000 ranked covariates.......51
Supplementary Figure 32 Diagnostic plots for high-dimensional propensity score weighted analysis for COVID-19 deaths, including triple therapy users, including the top 100 ranked covariates. 52
Supplementary Figure 33 Diagnostic plots for high-dimensional propensity score weighted analysis for COVID-19 deaths, including triple therapy users, including the top 250 ranked covariates. 53
Supplementary Figure 34 Diagnostic plots for high-dimensional propensity score weighted analysis for COVID-19 deaths, including triple therapy users, including the top 500 ranked covariates. 54
Supplementary Figure 35 Diagnostic plots for high-dimensional propensity score weighted analysis for COVID-19 deaths, including triple therapy users, including the top 750 ranked covariates. 55
Supplementary Figure 36 Diagnostic plots for high-dimensional propensity score weighted analysis for COVID-19 deaths, including triple therapy users, including the top 1000 ranked covariates. 56
Supplementary Figure 37 Diagnostic plots for high-dimensional propensity score weighted analysis for COVID-19 deaths, excluding triple therapy users, including the top 100 ranked covariates.......................57
Supplementary Figure 38 Diagnostic plots for high-dimensional propensity score weighted analysis for COVID-19 deaths, excluding triple therapy users, including the top 250 ranked covariates.......................58
Supplementary Figure 39 Diagnostic plots for high-dimensional propensity score weighted analysis for COVID-19 deaths, excluding triple therapy users, including the top 500 ranked covariates.......................59
Supplementary Figure 40 Diagnostic plots for high-dimensional propensity score weighted analysis for COVID-19 deaths, excluding triple therapy users, including the top 750 ranked covariates.......................60
Supplementary Figure 41 Diagnostic plots for high-dimensional propensity score weighted analysis for COVID-19 deaths, excluding triple therapy users, including the top 1000 ranked covariates.....................61

2.6. **Cox proportional hazards models...62**
Supplementary Figure 42 Kaplan-Meier curves for COVID-19 hospitalisation, including triple therapy users, weighted using prespecified covariates 62
Supplementary Figure 43 Kaplan-Meier curves for COVID-19 death, including triple therapy users, weighted using prespecified covariates...63

2.7. **Logistic regression models 64**
Supplementary Figure 44 Forest plot of odds ratios and 95% confidence intervals for COVID-19 hospitalisations, comparing ICS/LABA (+/- LAMA) users to LABA/LAMA users. 64

Supplementary Figure 45 Forest plot of odds ratios and 95% confidence intervals for COVID-19 deaths, comparing ICS/LABA (+/- LAMA) users to LABA/LAMA users. 64

2.8. Risk differences 65

Supplementary Figure 46 Risk differences for the outcome COVID-19 hospitalisation 65
Supplementary Figure 47 Risk differences for the outcome COVID-19 death....................65
Supplementary Figure 48 Unadjusted (left) and adjusted (right) survival curves for COVID-19 hospitalisation, including triple therapy users. 66
Supplementary Figure 49 Unadjusted (left) and adjusted (right) survival curves for COVID-19 hospitalisation, excluding triple therapy users. 66
Supplementary Figure 50 Unadjusted (left) and adjusted (right) survival curves for COVID-19 death, including triple therapy users. 67
Supplementary Figure 51 Unadjusted (left) and adjusted (right) survival curves for COVID-19 death, excluding triple therapy users....................67

1. Supplementary tables

Supplementary Table 1 100 most frequent unmatched SNOMED-CT codes.

Rank	SnomedCTConceptId	N	Term
1	1572871000006100	2646163	Awaiting clinical code migration to EMIS Web
2	279991000000102	2551983	SMS text message sent to patient
3	279991000000102	2551983	SMS (short message service) text message sent to patient
4	279991000000102	2551983	Short message service text message sent to patient
5	498521000006103	1525015	Attachment
6	72313002	668205	Systolic arterial pressure
7	72313002	668205	Systolic blood pressure
8	1091811000000100	667919	Diastolic arterial pressure
9	163020007	660161	O/E - blood pressure reading
10	163020007	660161	O/E - blood pressure
11	163020007	660161	O/E - BP reading
12	163020007	660161	O/E-blood pressure reading NOS
13	163020007	660161	On examination - blood pressure reading
14	78564009	433362	Pulse rate
15	78564009	433362	PR - Pulse rate
16	1000731000000100	415607	Serum creatinine NOS
17	1000731000000100	415607	Serum creatinine level
18	1000661000000100	388221	Serum sodium level
19	1000651000000100	386693	Serum potassium level
20	27113001	356678	Body weight
21	27113001	356678	O/E - weight NOS
22	27113001	356678	Weight
23	60621009	345560	Body mass index
24	60621009	345560	Weight: body mass
25	60621009	345560	BMI - Body mass index
26	1022431000000100	331357	Haemoglobin estimation NOS
27	1022431000000100	331357	Hb estimation
28	1022431000000100	331357	Haemoglobin estimation
29	1022541000000100	330596	Total white cell count NOS
30	1022541000000100	330596	Total white blood count
31	1022541000000100	330596	Total white blood count
32	1022541000000100	330596	White blood count
33	1022541000000100	330596	White cell count
34	1022541000000100	330596	Total white cell count
35	1022651000000100	327523	Platelet count
36	1022651000000100	327523	Platelet count NOS
37	1022551000000100	325184	Neutrophil count
38	1022551000000100	325184	Granulocyte count
39	1022551000000100	325184	Granulocyte count
40	1022491000000100	323940	Mean cell volume
41	1022491000000100	323940	MCV - Mean corpuscular volume
42	1022491000000100	323940	Mean cell volume
43	1022581000000100	321991	Lymphocyte count
44	1022561000000100	321775	Eosinophil count
45	1022561000000100	321775	Eosinophil count NOS
46	1022591000000100	321339	Monocyte count
47	1022591000000100	321339	Monocyte count NOS
48	1022471000000100	321091	Mean cell haemoglobin
49	1022471000000100	321091	MCH - Mean corpuscular haemoglobin
50	1022471000000100	321091	Mean cell haemoglobin
51	1022291000000100	318114	Packed cell volume

**52**	1022291000000100	318114	Haematocrit - PCV
**53**	1022291000000100	318114	Haematocrit
**54**	1022291000000100	318114	Packed cell volume - PCV
**55**	1022291000000100	318114	Haematocrit - packed cell volume
**56**	1022291000000100	318114	Packed cell volume
**57**	1000821000000100	317583	Serum albumin level
**58**	1022451000000100	316277	RBC count NOS
**59**	1022451000000100	316277	Red blood cell count
**60**	1022451000000100	316277	RBC (red blood cell) count
**61**	1022451000000100	316277	Erythrocyte count
**62**	1022571000000100	313054	Basophil count
**63**	1000951000000100	309980	Serum urea level
**64**	1000621000000100	287786	Serum alkaline phosphatase level
**65**	25611000000107	271414	Referral letter
**66**	1022441000000100	270864	FBC - full blood count
**67**	1018251000000100	270030	ALT/SGPT serum level
**68**	1018251000000100	270030	Serum alanine aminotransferase level
**69**	14734007	262161	Administrative procedure
**70**	14734007	262161	Administrative procedures
**71**	428481002	249980	Patient mobile telephone number
**72**	1022481000000100	244162	MCHC - Mean corpuscular haemoglobin concentration
**73**	431314004	241071	Peripheral oxygen saturation
**74**	431314004	241071	Pulse oximetry monitoring
**75**	431314004	241071	SpO2 - oxygen saturation at periphery
**76**	431314004	241071	Pulse oximetry
**77**	431314004	241071	SpO2 - saturation of peripheral oxygen
**78**	997531000000108	228728	Liver function test
**79**	394703002	228234	Chronic obstructive pulmonary disease annual review
**80**	999791000000106	227487	Haemoglobin A1c level - International Federation of Clinical Chemistry and Laboratory Medicine standardised
**81**	313334002	222174	Blood sample taken
**82**	313334002	222174	Nursing care blood sample taken
**83**	1000971000000100	211145	Urea and electrolytes level
**84**	248333004	208537	Standing height
**85**	248333004	208537	O/E - height NOS
**86**	997591000000109	208053	Serum total bilirubin level
**87**	1020291000000100	204903	GFR (glomerular filtration rate) calculated by abbreviated Modification of Diet in Renal Disease Study Group calculation
**88**	2051000000104	201713	Letter sent to patient
**89**	993501000000105	201207	Red blood cell distribution width
**90**	993501000000105	201207	RBC (red blood cell) distribution width
**91**	1000811000000100	194578	Serum total protein
**92**	1011481000000100	192330	eGFR (estimated glomerular filtration rate) using creatinine Chronic Kidney Disease Epidemiology Collaboration equation per 1.73 square metres
**93**	713636003	175318	Frailty Index score
**94**	1005681000000100	174486	Serum high density lipoprotein cholesterol level
**95**	1005681000000100	174486	Serum HDL (high density lipoprotein) cholesterol level
**96**	270426007	172311	Did not attend - no reason
**97**	270426007	172311	DNA - Did not attend - no reason
**98**	415974002	167484	Tympanic temperature
**99**	1022791000000100	166360	Serum TSH (thyroid stimulating hormone) level
**100**	1005671000000100	163080	Serum cholesterol NOS

Supplementary Table 2 Top 100 unmapped product codes

	ProdCodeId	Term.from.EMIS	DrugIssues
**1**	1572871000006117	Awaiting clinical code migration to EMIS Web	30000000
**2**	294711000000118	Transfer-degraded medication entry	6000000
**3**	619841000033115	Fybogel Granules 3.5 grams/sachet	2000000
**4**	643541000033114	Glyceryl Trinitrate Spray 400 micrograms/dose	2000000
**5**	1274141000033118	Senna Oral Solution, Sugar Free 7.5 mg/5 ml	2000000
**6**	2295041000033110	Doublebase Gel (Pump Dispenser)	2000000
**7**	2750641000033112	Cetraben Emollient Cream	2000000
**8**	783241000033112	Ispaghula Husk Sachets (orange) 3.5 g/sachet	1000000
**9**	1023241000033115	Oxygen Cylinder 1360 litres	1000000
**10**	1413741000033113	Temazepam Capsules 10 mg	1000000
**11**	1426941000033114	Terfenadine 60mg tablets	1000000
**12**	1843241000033118	Olive oil ear drops	1000000
**13**	2147541000033115	Rosiglitazone 4mg tablets	1000000
**14**	3232941000033115	Liquid Paraffin And Isopropyl Myristate Gel (Pump Dispenser) 15 % + 15 %	1000000
**15**	457641000033117	Dioralyte Oral powder	900000
**16**	783141000033117	Ispaghula Husk Sachets 3.5 g/sachet	900000
**17**	1803941000033119	Rofecoxib 12.5mg tablets	900000
**18**	1514841000033110	Vitamin Capsules Bpc Capsules	800000
**19**	4036141000033112	Sharpsafe Container 1 litre	800000
**20**	941541000033112	Multivitamin Capsules	700000
**21**	1431841000033111	Thick And Easy Powder	700000
**22**	1433641000033111	Thioridazine 10mg tablets	700000
**23**	1433841000033112	Thioridazine 25mg tablets	700000
**24**	1804041000033117	Rofecoxib 25mg tablets	700000
**25**	2221841000033111	Advantage Ii Test strips	700000
**26**	3996241000033117	Olanzapine 20mg tablets	700000
**27**	7687241000033110	Sitagliptin 50mg tablets	700000
**28**	468841000033112	Diltiazem Hydrochloride Tablets 60 mg	600000
**29**	621541000033110	Gamolenic acid 40mg capsules	600000
**30**	1470941000033111	Triludan Tablets 60 mg	600000
**31**	2571841000033116	Cavilon Durable Barrier cream 3392E	600000
**32**	2720841000033114	Ispaghula Husk Sugar and Gluten Free Effervescent granules (orange) 3.5 grams/sachet	600000
**33**	3159241000033117	Dermol Cream 500 gram bottle	600000
**34**	3196841000033116	Fortisip Bottle Liquid Feed (Mixed Flavours) Bottle 200 ml	600000
**35**	133841000033111	Beconase Nasal spray 50 micrograms/dose	500000
**36**	188241000033114	Calcipotriol 50micrograms/g cream	500000
**37**	444941000033119	Disposable Insulin syringe with needle 0.5 ml	500000
**38**	590341000033112	Flucloxacillin Oral suspension 125 mg/5 ml	500000
**39**	647341000033113	Glucotrend Test strips	500000
**40**	930641000033116	Monomax Sr 60 M/R capsules 60 mg	500000
**41**	974041000033118	Nitrolingual Spray 400 micrograms/dose	500000
**42**	1043841000033118	Pasteur Merieux Inactivated Influenza Vaccine 0.5 ml	500000
**43**	1413941000033111	Temazepam Capsules 20 mg	500000
**44**	2147641000033119	Rosiglitazone 8mg tablets	500000
**45**	4438341000033116	Ensure Plus Liquid feed (mixed flavours) Milkshake Style	500000
**46**	196441000033112	Calpol Paediatric Suspension 120 mg/5 ml	400000
**47**	287341000033117	Clarityn Tablets 10 mg	400000
**48**	586341000033110	Fluticasone Propionate Inhaler 250 micrograms/puff	400000
**49**	596541000033119	Fluzone Vaccine 0.5 ml	400000
**50**	652141000033110	Graduated Compression Hosiery below knee class 2	400000
**51**	726341000033119	Human Mixtard 30 Penfill cartridges (3 ml)	400000
**52**	792241000033116	Juvela gluten free loaf sliced (Hero UK Ltd)	400000
**53**	906041000033116	Mfv-Ject Prefilled syringe	400000
**54**	1252341000033111	Salbutamol Rotacaps 400 micrograms	400000

**55**	1358941000033119	Sodium Valproate Sugar-free liquid 200 mg/5 ml	400000
**56**	1804941000033116	Ensure Plus Liquid Feed (Mixed Flavours) Tetrapak 220 ml	400000
**57**	2207041000033119	Glyceryl Trinitrate Cfc-free pump spray 400 micrograms/dose (180 dose)	400000
**58**	3232341000033119	White Soft Paraffin And Liquid Paraffin Light Cream (Pump Dispenser) 13.2 % + 10.5 %	400000
**59**	5733041000033111	Hypodermic insulin needles for pre-filled / reusable pen injectors screw on 4mm/32gauge	400000
**60**	6381541000033111	Vita-Pos Eye ointment (preservative-free)	400000
**61**	288341000033118	Clostet Vaccine	300000
**62**	421741000033116	Depo-Provera Injection 150 mg/1 ml	300000
**63**	497541000033112	Efamast 40 Capsules 40 mg	300000
**64**	525441000033115	Epogam Capsules 40 mg	300000
**65**	644241000033114	Glycerol Suppositories	300000
**66**	651841000033113	Graduated Compression Hosiery thigh length class 2	300000
**67**	773641000033116	Ipratropium Bromide Nebuliser solution 250 micrograms/ml	300000
**68**	791141000033115	Juvela gluten free fibre loaf sliced (Hero UK Ltd)	300000
**69**	791741000033116	Juvela gluten free mix (Hero UK Ltd)	300000
**70**	943141000033116	Mucaine Suspension	300000
**71**	970441000033115	Nicotine Inhalation cartridge with mouthpiece (refill) 10 mg/cartridge	300000
**72**	1044341000033113	Peppermint Oil Capsules 0.2 ml	300000
**73**	1064641000033111	Penicillin Vk Tablets 250 mg	300000
**74**	1264041000033110	Scanpor Adhesive tape 2.5 cm x 5 m	300000
**75**	1420541000033118	Tetavax Injection	300000
**76**	1420641000033117	Tetavax Injection	300000
**77**	1433941000033116	Thioridazine 50mg tablets	300000
**78**	1477641000033115	Tubigrip Elasticated Support Bandage Stockinette 8.75 cm x 1 m (e)	300000
**79**	1618941000033118	Instillagel Gel (11 Ml Syringe)	300000
**80**	1619041000033110	Instillagel Gel (6 Ml Syringe)	300000
**81**	1728741000033117	Elasticated Viscose Stockinette 10.75 cm x 5 m (yellow line)	300000
**82**	1728941000033119	Elasticated Viscose Stockinette 7.5 cm x 5 m (blue line)	300000
**83**	1754341000033113	Tubifast 2-Way Stretch Stockinette 7.5 cm x 5 m (blue line)	300000
**84**	2206941000033115	Magnesium Hydroxide Mixture BP	300000
**85**	2207241000033110	Nutriprem 2 Powder 900 grams	300000
**86**	2271241000033111	Carmellose Sodium Eye-drops (unit dose) 1 %	300000
**87**	2527941000033118	Penfine Needles for insulin pens 31g, 8 mm	300000
**88**	3160241000033116	Sodium Alginate And Potassium Bicarbonate Oral Suspension Sugar Free Peppermint, 500 mg + 100 mg/5 ml	300000
**89**	3169041000033119	Thick And Easy Powder 225 gram tin	300000
**90**	3335541000033112	Senna 15mg tablets	300000
**91**	3346841000033117	Losartan 100mg / Hydrochlorothiazide 25mg tablets	300000
**92**	3925841000033118	Juvela gluten free fresh white loaf sliced (Hero UK Ltd)	300000
**93**	4387641000033111	Pregabalin 225mg capsules	300000
**94**	4898841000033112	Tadalafil 5mg tablets	300000
**95**	5072541000033112	Fortisip Bottle (Flavour Not Specified)	300000
**96**	5234741000033116	Ensure Plus milkshake style liquid (Flavour Not Specified)	300000
**97**	5734741000033112	Fortisip Compact liquid (Flavour Not Specified)	300000
**98**	16141000033110	Adsorbed Tetanus Vaccine Bp Injection	200000
**99**	51641000033119	Amphotericin B 10mg lozenges sugar free	200000
**100**	87341000033112	Asacol E/c tablets 400 mg	200000

Supplementary Table 3 Bias Information for COVID-19 hospitalisations, with triple therapy users.

	variable		e1c1	e0c1	e1c0	e0c0	d1c1	d0c1	d1c0	d0c0	rrCE	rrCD	absLogBias
1	d2_030700_once	Mucolytics	11152	2437	44877	19870	199	13390	463	64284	1.822	2.048	0.081
2	d3_J44_freq	Other chronic obstructive pulmonary disease	6276	1838	49753	20469	231	7883	431	69791	1.359	4.638	0.080
3	d3_J44_spor	Other chronic obstructive pulmonary disease	10472	3248	45557	19059	295	13425	367	64249	1.284	3.786	0.079
4	d2_030700_spor	Mucolytics	6438	1213	49591	21094	131	7520	531	70154	2.113	2.279	0.070
5	d3_Z86_spor	Personal history of certain other diseases	6277	1949	49752	20358	229	7997	433	69677	1.282	4.508	0.064
6	d3_J44_once	Other chronic obstructive pulmonary disease	17904	6016	38125	16291	370	23550	292	54124	1.185	2.883	0.060
7	d3_J18_once	Pneumonia, organism unspecified	2965	819	53064	21488	133	3651	529	74023	1.441	4.953	0.054
8	d3_Z86_freq	Personal history of certain other diseases	3703	1159	52326	21148	173	4689	489	72985	1.272	5.346	0.049
9	d2_050101_spor	Penicillins	15021	3909	41008	18398	222	18708	440	58966	1.530	1.583	0.048
10	d3_J18_spor	Pneumonia, organism unspecified	2036	548	53993	21759	106	2478	556	75196	1.479	5.589	0.047
11	d2_060302_spor	Glucocorticoid therapy	18726	3976	37303	18331	231	22471	431	55203	1.875	1.313	0.045
12	d1_F17_once	Mental and behavioural disorders due to use of tobacco	18384	8620	37645	13687	114	26890	548	50784	0.849	0.395	0.045
13	d3_Z86_once	Personal history of certain other diseases	10076	3442	45953	18865	275	13243	387	64431	1.165	3.407	0.044
14	d2_050101_freq	Penicillins	8735	1900	47294	20407	137	10498	525	67176	1.830	1.661	0.043
15	d2_050401_once	Antimalarials	20076	5445	35953	16862	269	25252	393	52422	1.468	1.417	0.042
16	d3_J18_freq	Pneumonia, organism unspecified	1037	261	54992	22046	77	1221	585	76453	1.582	7.812	0.042
17	d2_030700_freq	Mucolytics	3016	550	53013	21757	70	3496	592	74178	2.183	2.479	0.041
18	d2_020202_once	Loop diuretics	9778	3315	46251	18992	254	12839	408	64835	1.174	3.102	0.041
19	d2_060302_freq	Glucocorticoid therapy	8738	1547	47291	20760	122	10163	540	67511	2.249	1.495	0.041
20	d2_060302_once	Glucocorticoid therapy	28916	7628	27113	14679	344	36200	318	41474	1.509	1.237	0.037
21	d2_020202_spor	Loop diuretics	4940	1619	51089	20688	168	6391	494	71283	1.215	3.722	0.035
22	d2_030101_spor	Adrenoceptor agonists	30352	8636	25677	13671	365	38623	297	39051	1.399	1.240	0.033
23	d3_Z50_once	Care involving use of rehabilitation procedures	2603	796	53426	21511	113	3286	549	74388	1.302	4.538	0.033
24	d3_Z50_spor	Care involving use of rehabilitation procedures	1746	508	54283	21799	91	2163	571	75511	1.368	5.379	0.033
25	d2_090604_once	Vitamin D	12476	4135	43553	18172	236	16375	426	61299	1.201	2.059	0.032
26	d2_090604_spor	Vitamin D	6584	2077	49445	20230	156	8505	506	69169	1.262	2.480	0.031
27	d1_F17_spor	Mental and behavioural disorders due to use of tobacco	11329	5467	44700	16840	62	16734	600	60940	0.825	0.379	0.031
28	d3_R29_once	Other symptoms and signs involving the nervous and musculoskeletal systems	1497	443	54532	21864	85	1855	577	75819	1.345	5.801	0.030
29	d3_R29_spor	Other symptoms and signs involving the nervous and musculoskeletal systems	989	274	55040	22033	68	1195	594	76479	1.437	6.986	0.029
30	d2_050401_spor	Antimalarials	11722	2631	44307	19676	153	14200	509	63474	1.774	1.340	0.029

* e1c1 = number of exposed (ICS) with the covariate, e0c1 = number unexposed with the covariate, e1c0 = number exposed without the covariate, e0c0 = number exposed without the covariate, d1c1 = number with outcome (COVID-19 hospitalisation) with the covariate, d0c1 = number without the outcome with the covariate, d1c0 = number with the outcome without the covariate, d0c0 = number without the outcome without the covariate, rrCE = relative risk between covariate and exposure, rrCD = relative risk between covariate and outcome, absLogBias is calculated using the Bross formula. In the variable column, d1 refers to primary care clinical observations, d2 to primary care prescriptions, and d3 to hospital data.

Supplementary Table 4 Bias Information for COVID-19 hospitalisations, without triple therapy users

	variable		e1c1	e0c1	e1c0	e0c0	d1c1	d0c1	d1c0	d0c0	rrCE	rrCD	absLogBias
1	d1_F17_once	Mental and behavioural disorders due to use of tobacco	4609	8620	10296	13687	38	13191	202	23781	0.800	0.341	0.066
2	d1_F17_spor	Mental and behavioural disorders due to use of tobacco	2706	5467	12199	16840	15	8158	225	28814	0.741	0.237	0.058
3	d1_Z71_once	Persons encountering health services for other counselling and medical advice, not elsewhere classified	7168	12102	7737	10205	79	19191	161	17781	0.886	0.457	0.046
4	d1_Z71_spor	Persons encountering health services for other counselling and medical advice, not elsewhere classified	3417	6334	11488	15973	27	9724	213	27248	0.807	0.357	0.042
5	d3_R29_spor	Other symptoms and signs involving the nervous and musculoskeletal systems	264	274	14641	22033	23	515	217	36457	1.442	7.225	0.031
6	d3_R29_once	Other symptoms and signs involving the nervous and musculoskeletal systems	405	443	14500	21864	28	820	212	36152	1.368	5.664	0.031
7	d3_N39_once	Other disorders of urinary system	342	365	14563	21942	24	683	216	36289	1.402	5.737	0.029
8	d1_F17_freq	Mental and behavioural disorders due to use of tobacco	1386	2930	13519	19377	10	4306	230	32666	0.708	0.331	0.028
9	d1_J42_once	Unspecified chronic bronchitis	1413	1703	13492	20604	47	3069	193	33903	1.242	2.665	0.027
10	d3_J96_once	Respiratory failure, not elsewhere classified	205	477	14700	21830	19	663	221	36309	0.643	4.605	0.026
11	d2_030102_once	Antimuscarinic bronchodilators	583	140	14322	22167	8	715	232	36257	6.232	1.740	0.024
12	d3_W19_once	Unspecified fall	172	168	14733	22139	15	325	225	36647	1.532	7.230	0.024
13	d1_J44_once	Other chronic obstructive pulmonary disease	4481	8080	10424	14227	61	12500	179	24472	0.830	0.669	0.023
14	d2_010602_freq	Stimulant laxatives	276	299	14629	22008	20	555	220	36417	1.381	5.792	0.023
15	d2_050101_freq	Penicillins	3030	3909	11875	18398	74	6865	166	30107	1.160	1.945	0.022
16	d1_J42_freq	Unspecified chronic bronchitis	391	425	14514	21882	21	795	219	36177	1.377	4.277	0.022
17	d2_010602_spor	Stimulant laxatives	499	590	14406	21717	28	1061	212	35911	1.266	4.381	0.022
18	d3_F03_once	Unspecified dementia	146	111	14759	22196	9	248	231	36724	1.969	5.602	0.021
19	d1_Z75_once	Problems related to medical facilities and other health care	9	2	14896	22305	3	8	237	36964	6.735	42.809	0.021
20	d2_090604_freq	Vitamin D	972	1261	13933	21046	46	2187	194	34785	1.154	3.714	0.020
21	d3_N39_spor	Other disorders of urinary system	182	188	14723	22119	15	355	225	36617	1.449	6.638	0.020
22	d3_J45_once	Asthma	547	296	14358	22011	10	833	230	36139	2.766	1.876	0.020
23	d2_021200_once	Lipid-regulating drugs	7650	12214	7255	10093	163	19701	77	17271	0.937	1.849	0.020
24	d2_090604_spor	Vitamin D	1572	2077	13333	20230	58	3591	182	33381	1.133	2.931	0.020
25	d3_K59_once	Other functional intestinal disorders	300	344	14605	21963	21	623	219	36349	1.305	5.445	0.019
26	d1_E94_once	Bronchial tests	1641	3355	13264	18952	19	4977	221	31995	0.732	0.554	0.019
27	d2_010602_once	Stimulant laxatives	919	1213	13986	21094	46	2086	194	34886	1.134	3.901	0.018
28	d2_010604_once	Osmotic laxatives	1804	2425	13101	19882	62	4167	178	32805	1.113	2.717	0.018
29	d1_W19_once	Unspecified fall	444	515	14461	21792	22	937	218	36035	1.290	3.815	0.018
30	d3_I50_spor	Heart failure	349	627	14556	21680	29	947	211	36025	0.833	5.103	0.017

* e1c1 = number of exposed (ICS) with the covariate, e0c1 = number of unexposed with the covariate, e1c0 = number exposed without the covariate, e0c0 = number exposed without the covariate, d1c1 = number with outcome (COVID-19 hospitalisation) with the covariate, d0c1 = number without the outcome with the covariate, d1c0 = number with the outcome without the covariate, d0c0 = number without the outcome without the covariate, rrCE = relative risk between covariate and exposure, rrCD = relative risk between covariate and outcome, absLogBias is calculated using the Bross formula. In the variable column, d1 refers to primary care clinical observations, d2 to primary care prescriptions, and d3 to hospital data.

Supplementary Table 5 Bias Information for COVID-19 deaths, with triple therapy users

	variable		e1c1	e0c1	e1c0	e0c0	d1c1	d0c1	d1c0	d0c0	rrCE	rrCD	absLogBias
1	d3_J44_freq	Other chronic obstructive pulmonary disease	6276	1838	49753	20469	126	7988	240	69982	1.359	4.544	0.078
2	d3_J44_spor	Other chronic obstructive pulmonary disease	10472	3248	45557	19059	159	13561	207	64409	1.284	3.618	0.075
3	d3_J44_once	Other chronic obstructive pulmonary disease	17904	6016	38125	16291	204	23716	162	54254	1.185	2.865	0.060
4	d3_J18_once	Pneumonia, organism unspecified	2965	819	53064	21488	78	3706	288	74264	1.441	5.336	0.059
5	d3_Z86_spor	Personal history of certain other diseases	6277	1949	49752	20358	116	8110	250	69860	1.282	3.955	0.056
6	d3_J18_spor	Pneumonia, organism unspecified	2036	548	53993	21759	65	2519	301	75451	1.479	6.331	0.054
7	d2_030101_spor	Adrenoceptor agonists	30352	8636	25677	13671	213	38775	153	39195	1.399	1.405	0.053
8	d3_Z86_freq	Personal history of certain other diseases	3703	1159	52326	21148	96	4766	270	73204	1.272	5.373	0.049
9	d3_J18_freq	Pneumonia, organism unspecified	1037	261	54992	22046	48	1250	318	76720	1.582	8.959	0.048
10	d2_030700_spor	Mucolytics	6438	1213	49591	21094	61	7590	305	70380	2.113	1.848	0.048
11	d2_030700_once	Mucolytics	11152	2437	44877	19870	91	13498	275	64472	1.822	1.577	0.048
12	d2_030700_freq	Mucolytics	3016	550	53013	21757	42	3524	324	74446	2.183	2.718	0.047
13	d2_090604_once	Vitamin D	12476	4135	43553	18172	151	16460	215	61510	1.201	2.610	0.045
14	d2_090604_spor	Vitamin D	6584	2077	49445	20230	106	8555	260	69415	1.262	3.280	0.045
15	d2_020202_once	Loop diuretics	9778	3315	46251	18992	147	12946	219	65024	1.174	3.345	0.044
16	d2_050101_spor	Penicillins	15021	3909	41008	18398	119	18811	247	59159	1.530	1.512	0.043
17	d1_F17_once	Mental and behavioural disorders due to use of tobacco	18384	8620	37645	13687	67	26937	299	51033	0.849	0.426	0.042
18	d2_090604_freq	Vitamin D	4052	1261	51977	21046	84	5229	282	72741	1.279	4.094	0.041
19	d2_020202_spor	Loop diuretics	4940	1619	51089	20688	103	6456	263	71514	1.215	4.286	0.041
20	d3_Z86_once	Personal history of certain other diseases	10076	3442	45953	18865	144	13374	222	64596	1.165	3.110	0.040
21	d2_030101_freq	Adrenoceptor agonists	15018	3715	41011	18592	113	18620	253	59350	1.609	1.421	0.039
22	d3_R29_spor	Other symptoms and signs involving the nervous and musculoskeletal systems	989	274	55040	22033	47	1216	319	76754	1.437	8.991	0.038
23	d3_R29_once	Other symptoms and signs involving the nervous and musculoskeletal systems	1497	443	54532	21864	57	1883	309	76087	1.345	7.264	0.038
24	d1_E94_once	Bronchial tests	5620	3355	50409	18952	16	8959	350	69011	0.667	0.353	0.035
25	d3_Z50_once	Care involving use of rehabilitation procedures	2603	796	53426	21511	64	3335	302	74635	1.302	4.672	0.034
26	d2_010604_once	Osmotic laxatives	7191	2425	48838	19882	110	9506	256	68464	1.181	3.071	0.033
27	d3_Z50_spor	Care involving use of rehabilitation procedures	1746	508	54283	21799	50	2204	316	75766	1.368	5.341	0.033
28	d2_020202_freq	Loop diuretics	3239	1043	52790	21264	74	4208	292	73762	1.236	4.383	0.032
29	d3_E87_once	Other disorders of fluid, electrolyte and acid-base balance	1988	607	54041	21700	56	2539	310	75431	1.304	5.273	0.031
30	d3_I10_freq	Essential (primary) hypertension	3525	1168	52504	21139	82	4611	284	73359	1.202	4.531	0.031

* e1c1 = number exposed (ICS) with the covariate, e0c1 = number of unexposed with the covariate, e1c0 = number exposed without the covariate, e0c0 = number exposed without the covariate, d1c1 = number with outcome (COVID-19 death) with the covariate, d0c1 = number without the outcome with the covariate, d1c0 = number with the outcome without the covariate, d0c0 = number without the outcome without the covariate, rrCE = relative risk between covariate and exposure, rrCD = relative risk between covariate and outcome, absLogBias is calculated using the Bross formula. In the variable column, d1 refers to primary care clinical observations, d2 to primary care prescriptions, and d3 to hospital data.

Supplementary Table 6 Bias Information for COVID-19 deaths, without triple therapy users

	variable		e1c1	e0c1	e1c0	e0c0	d1c1	d0c1	d1c0	d0c0	rrCE	rrCD	absLogBias
1	d1_F17_once	Mental and behavioural disorders due to use of tobacco	4609	8620	10296	13687	20	13209	114	23869	0.800	0.318	0.069
2	d1_Z71_once	Persons encountering health services for other counselling and medical advice, not elsewhere classified	7168	12102	7737	10205	37	19233	97	17845	0.886	0.355	0.059
3	d1_F17_spor	Mental and behavioural disorders due to use of tobacco	2706	5467	12199	16840	9	8164	125	28914	0.741	0.256	0.056
4	d3_R29_once	Other symptoms and signs involving the nervous and musculoskeletal systems	405	443	14500	21864	21	827	113	36251	1.368	7.969	0.044
5	d2_041100_once	Drugs for dementia	238	258	14667	22049	19	477	115	36601	1.381	12.230	0.043
6	d3_R29_spor	Other symptoms and signs involving the nervous and musculoskeletal systems	264	274	14641	22033	17	521	117	36557	1.442	9.905	0.043
7	d2_041100_spor	Drugs for dementia	135	128	14770	22179	13	250	121	36828	1.578	15.094	0.042
8	d3_F03_once	Unspecified dementia	146	111	14759	22196	9	248	125	36830	1.969	10.353	0.042
9	d1_Z71_spor	Persons encountering health services for other counselling and medical advice, not elsewhere classified	3417	6334	11488	15973	16	9735	118	27343	0.807	0.382	0.040
10	d3_J45_once	Asthma	547	296	14358	22011	8	835	126	36243	2.766	2.739	0.039
11	d2_091316_once	Thickener	75	54	14830	22253	7	122	127	36956	2.079	15.845	0.037
12	d2_090604_freq	Vitamin D	972	1261	13933	21046	38	2195	96	34883	1.154	6.201	0.034
13	d2_090604_spor	Vitamin D	1572	2077	13333	20230	46	3603	88	33475	1.133	4.808	0.034
14	d1_E94_once	Bronchial tests	1641	3355	13264	18952	5	4991	129	32087	0.732	0.250	0.034
15	d3_N39_once	Other disorders of urinary system	342	365	14563	21942	15	692	119	36386	1.402	6.508	0.033
16	d3_G30_once	Alzheimer disease	78	68	14827	22239	8	138	126	36940	1.717	16.119	0.031
17	d1_J42_once	Unspecified chronic bronchitis	1413	1703	13492	20604	28	3088	106	33990	1.242	2.890	0.030
18	d3_F00_once	Dementia in Alzheimer disease	59	49	14846	22258	7	101	127	36977	1.802	18.936	0.030
19	d2_050101_freq	Penicillins	3030	3909	11875	18398	46	6893	88	30185	1.160	2.281	0.029
20	d3_F03_spor	Unspecified dementia	89	67	14816	22240	6	150	128	36928	1.988	11.135	0.029
21	d2_010604_spor	Osmotic laxatives	1130	1443	13775	20864	30	2543	104	34535	1.172	3.883	0.027
22	d1_F17_freq	Mental and behavioural disorders due to use of tobacco	1386	2930	13519	19377	6	4310	128	32768	0.708	0.357	0.027
23	d2_010602_spor	Stimulant laxatives	499	590	14406	21717	18	1071	116	36007	1.266	5.147	0.026
24	d3_W19_once	Unspecified fall	172	168	14733	22139	9	331	125	36747	1.532	7.808	0.026
25	d2_010604_once	Osmotic laxatives	1804	2425	13101	19882	43	4186	91	32892	1.113	3.685	0.025
26	d2_010602_freq	Stimulant laxatives	276	299	14629	22008	12	563	122	36515	1.381	6.267	0.025
27	d2_212200_spor	Emollients	1046	1337	13859	20970	27	2356	107	34722	1.171	3.688	0.023
28	d1_E97_once	Respiratory education	2422	5044	12483	17263	19	7447	115	29631	0.719	0.658	0.023
29	d2_090604_once	Vitamin D	2979	4135	11926	18172	58	7056	76	30022	1.078	3.229	0.023
30	d1_J42_freq	Unspecified chronic bronchitis	391	425	14514	21882	12	804	122	36274	1.377	4.387	0.023

* e1c1 = number exposed (ICS) with the covariate, e0c1 = number of unexposed with the covariate, e1c0 = number exposed without the covariate, e0c0 = number exposed without the covariate, d1c1 = number with outcome (COVID-19 death) with the covariate, d0c1 = number without the outcome with the covariate, d1c0 = number with the outcome without the covariate, d0c0 = number without the outcome without the covariate, rrCE = relative risk between covariate and exposure, rrCD = relative risk between covariate and outcome, absLogBias is calculated using the Bross formula. In the variable column, d1 refers to primary care clinical observations, d2 to primary care prescriptions, and d3 to hospital data.

Supplementary Table 7 Summary of decisions related to the implementation of HDPS

1	Specify data dimensions	Dimensions identified and which aspect of the healthcare system they characterise	The data was separated into three dimensions reflecting different care facets:<br>1. Primary care observations from Clinical Practice Research Datalink (CPRD) Aurum<br>2. Primary care prescriptions from CPRD Aurum<br>3. Hospital admission diagnoses from Hospital Episode Statistics (HES) Admitted Patient Care
2	Describe parameters for generating pre-exposure features	Describe how features are generated	Clinical observations in CPRD Aurum (SNOMED-CT codes) were mapped to ICD-10 codes (truncated to three characters). Prescriptions (dm+d codes) were mapped to British National Formulary (BNF) paragraphs. Unmatched dm+d codes with over 1,000,000 recordings were mapped manually.<br>Diagnoses in HES APC are coded using ICD-10, which were truncated to three characters. Occurrence of codes was assessed in the 12 months before the index date. For the primary care clinical observation dimension, we considered codes that were ever recorded in the data.
		Number of codes selected per dimension in prevalence filter	No prevalence filter was applied.
3	Describe feature recurrence assessment	Whether and how recurrence was considered	For each patient, variables indicated if a code was recorded at least once (≥1), sporadically (≥median), or frequently (≥75%-ile) in the year before the index date, relative to the number of occurrences among those with ≥1 occurrence. For the primary care observation dimension, ever-present codes were considered to reflect chronic conditions not necessarily recorded at every consultation.
		Whether and how proximity to exposure start was considered	Pre-exposure features were based on the occurrence of codes in the 12 months prior to the index date (exposure start)
4	Specify covariate prioritisation method	Ranking based on:<br>- Exposure-outcome prediction based (Bross)<br>- ML-supported exposure-outcome prediction<br>- Exposure prediction only	Codes were ranked using the Bross formula.
5	Specify total number of covariates to select	Number of HDPS covariates selected	100, 250, 500, 750, 1000
		Justification for number of HDPS covariates selected, e.g. use of simulation-based approaches	No number was specified as a primary analysis. Instead, sensitivity to the number of covariates was explored. A post-hoc analysis added covariates ranked 1st-250th one by one to assess the sensitivity of results to inclusion of individual covariates. The top 30 ranked covariates for each cohort and outcome are available in supplementary tables 3-6. Diagnostics for 250 HDPS covariates were presented for illustration

		Routine reporting of the investigator identified covariates	Prespecified covariates are listed in Methods – Prespecified Covariates. Standardised mean differences (SMDs) before and after weighting using the conventional propensity scores and HDPS are reported in Table 1, Supplementary Tables 7 and 8.
6	Specify software	Describe which software package was used to implement the HDPS procedure	R. Code lists and data management and analysis code are available on GitHub.
7	Describe the results of diagnostics and sensitivity analyses	Describe diagnostic tools used and highlight key insights gained	• Propensity score (PS) overlap was assessed graphically and by summarising PSs by treatment group. (Figure 1, supplementary figures 23-41) PSs were trimmed to the region of common support, which excluded very few patients (<1%).<br>• A summary of weights before and after trimming is in Supplementary Table 9<br>• SMDs were calculated for covariate before and after PS-weighting (conventional and HDPS). (Table 1, Figure 2, Supplementary Table 7 and 8, Supplementary figures 23-41)<br>• Conventional PS weighting balanced prespecified covariates (SMD < 0.1)<br>• HDPS weighting achieved adequate balance (SMD < 0.1) for predefined covariates and better balance for additional HDPS covariates.<br>• Summary of concepts captured by top HDPS covariates in Supplementary Figures 2-21.
		Describe the results of sensitivity analyses and discuss the possible implications for interpreting the findings from the primary analysis	• Results for COVID-19 hospitalisation varied based on the number of HDPS covariates included (e.g., excluding triple therapy, HR with 100 HDPS covariates was 1.01, with 250 HDPS covariates was 1.24).<br>• HDPS results can be sensitive to the number of covariates included, emphasizing the need for sensitivity analyses.<br>• For COVID-19 hospitalisations, point estimates moved closer to or further from the null depending on the exposure definition and number of covariates.<br>• For COVID-19 deaths, HDPS consistently moved point estimates closer to the null.<br>• Adding covariates one-by-one showed that even low-ranked, covariates could substantially change effect estimates, especially if patients with unusual covariate combinations experienced a rare outcome.<br>• In situations with unclear confounding structures (like COVID-19 hospitalisations), HDPS can yield inconsistent results sensitive to covariate inclusion, highlighting the importance of sensitivity analyses for interpretation

Supplementary Table 8 Baseline characteristics (Including triple therapy users)

	Unweighted				SMDs including HDPS for hospitalisations					SMDs after HDPS for deaths				
	LABA/LAMA (n = 22318)	ICS/LABA (n = 56049)	Unweighted SMD	SMD (weighted predefined covariates)	SMD (100 HDPS covariates)	SMD (250 HDPS covariates)	SMD (500 HDPS covariates)	SMD (750 HDPS covariates)	SMD (1000 HDPS covariates)	SMD (100 HDPS covariates)	SMD (250 HDPS covariates)	SMD (500 HDPS covariates)	SMD (750 HDPS covariates)	SMD (1000 HDPS covariates)
**Gender = Female**	10074 (45.1)	26228 (46.8)	0.033	0.005	0.003	0.002	0.001	0.001	0.001	0.002	0.001	0.004	0.004	0.002
**age (mean (SD))**	70.82 (10.23)	71.32 (10.47)	0.048	0.002	0.008	0.01	0.011	0.009	0.006	0.008	0.007	0.011	0.01	0.009
**IMD**			0.044	0.005	0.009	0.008	0.006	0.006	0.007	0.009	0.008	0.007	0.008	0.007
**1**	3047 (13.7)	7178 (12.8)												
**2**	3813 (17.1)	9212 (16.4)												
**3**	4077 (18.3)	9970 (17.8)												
**4**	5012 (22.5)	12719 (22.7)												
**5**	6357 (28.5)	16940 (30.2)												
**Missing**	12 (0.1)	30 (0.1)												
**Diabetes**	5517 (24.7)	14078 (25.1)	0.009	0.003	0.008	0.01	0.013	0.012	0.013	0.008	0.011	0.012	0.011	0.012
**Hypertension**	11318 (50.7)	28598 (51.0)	0.006	0.003	0.006	0.002	0.004	0.003	0.001	0.006	0.006	0.003	0.002	0.002
**Cardiovascular disease**	6539 (29.3)	16768 (29.9)	0.014	0.002	0.004	0.003	0.004	0.003	0.002	0.005	0.005	0.008	0.007	0.005
**Cancer**	4415 (19.8)	10542 (18.8)	0.025	0.001	0.004	0.005	0.006	0.006	0.003	0.005	0.008	0.01	0.011	0.01
**Past asthma**	2665 (11.9)	15336 (27.4)	0.396	0.002	0.013	0.022	0.022	0.02	0.02	0.013	0.012	0.014	0.012	0.015
**Chronic kidney disease**	6737 (30.2)	16722 (29.8)	0.008	0.007	0.012	0.012	0.013	0.011	0.009	0.013	0.012	0.013	0.012	0.012
**Immunosuppre ssion**	277 (1.2)	665 (1.2)	0.005	<0.001	<0.001	0.002	0.001	<0.001	0.001	0.001	0.004	0.003	0.005	0.003
**Influenza vaccine**	17961 (80.5)	44898 (80.1)	0.009	0.001	<0.001	<0.001	<0.001	0.002	0.005	0.001	0.002	0.003	0.001	0.003
**Pneumococcal vaccine**	3236 (14.5)	5985 (10.7)	0.115	0.001	<0.001	0.001	<0.001	<0.001	0.001	0.001	<0.001	<0.001	0.001	0.001
**COPD exacerbation past 12 months)**	6222 (27.9)	22799 (40.7)	0.272	0.002	0.003	0.005	0.006	0.008	0.009	0.002	<0.001	0.007	0.006	0.011
**Former smoking**	12246 (54.9)	33287 (59.4)	0.091	0.002	0.01	0.01	0.009	0.008	0.007	0.009	0.008	0.006	0.008	0.008
**Ethnicity (%)**			0.05	0.009	0.009	0.017	0.016	0.014	0.014	0.011	0.01	0.011	0.013	0.011

**White**	19584 (87.7)	49390 (88.1)												
**South Asian**	197 (0.9)	732 (1.3)												
**Black**	130 (0.6)	351 (0.6)												
**Mixed**	49 (0.2)	142 (0.3)												
**Unknown**	2358 (10.6)	5434 (9.7)												
**BMI category(%)**			0.07	0.004	0.007	0.008	0.008	0.006	0.007	0.007	0.009	0.007	0.006	0.006
**Underweight (<18.5)**	970 (4.3)	3140 (5.6)												
**Normal (18.5-24.9)**	6926 (31.0)	18155 (32.4)												
**Overweight (25-29.9)**	7171 (32.1)	17349 (31.0)												
**Obese (>=30)**	7251 (32.5)	17405 (31.1)												

Supplementary Table 9 Baseline characteristics (No triple therapy users)

	Unweighted				SMDs after HDPS for hospitalisations					SMDs after HDPS for deaths				
	LABA/LAMA (n = 22308)	ICS/LABA (n = 14905)	SMD unweighted	SMD (predefined covariates)	SMD (100 HDPS covariates)	SMD (250 HDPS covariates)	SMD (500 HDPS covariates)	SMD (750 HDPS covariates)	SMD (1000 HDPS covariates)	SMD (100 HDPS covariates)	SMD (250 HDPS covariates)	SMD (500 HDPS covariates)	SMD (750 HDPS covariates)	SMD (1000 HDPS covariates)
Gender = Female	10068 (45.1)	7089 (47.6)	0.049	0.001	0.001	0.003	0.005	0.006	0.006	<0.001	0.002	0.003	0.006	0.005
age (mean (SD))	70.83 (10.23)	71.37 (11.29)	0.051	0.002	0.002	0.002	<0.001	0.002	0.001	0.002	0.003	0.005	0.004	0.004
IMD			0.028	0.016	0.017	0.016	0.017	0.017	0.018	0.022	0.019	0.019	0.018	0.018
1	3048 (13.7)	2021 (13.6)												
2	3813 (17.1)	2562 (17.2)												
3	4077 (18.3)	2759 (18.5)												
4	5012 (22.5)	3473 (23.3)												
5	6357 (28.5)	4089 (27.4)												
Missing	1 (0.0)	1 (0.0)												
Diabetes	5515 (24.7)	3685 (24.7)	<0.001	0.001	0.002	0.001	0.001	0.001	0.001	0.002	<0.001	0.001	0.002	0.003
Hypertension	11313 (50.7)	7688 (51.6)	0.017	0.001	0.001	0.001	<0.001	0.002	0.002	<0.001	0.001	0.001	0.003	0.004
Cardiovascular disease	6538 (29.3)	4387 (29.4)	0.003	<0.001	0.001	0.001	0.003	0.004	0.002	0.001	0.001	0.001	0.001	0.001
Cancer	4414 (19.8)	2827 (19.0)	0.021	<0.001	0.002	0.003	0.006	0.007	0.006	0.001	0.004	0.004	0.005	0.005
Past asthma	2665 (11.9)	4134 (27.7)	0.404	<0.001	<0.001	0.001	0.002	0.005	0.006	<0.001	0.001	0.001	0.002	0.004
Kidney disease	6737 (30.2)	4566 (30.6)	0.009	0.001	0.004	0.006	0.01	0.01	0.006	0.002	0.006	0.005	0.007	0.007
Immunosuppression	274 (1.2)	188 (1.3)	0.003	<0.001	0.001	<0.001	0.003	0.004	0.004	<0.001	0.006	0.005	0.007	0.007
Influenza vaccine	17956 (80.5)	11390 (76.4)	0.099	<0.001	0.002	0.004	0.002	0.006	0.005	0.002	0.004	0.003	0.004	0.004
Pneumococcal vaccine	3236 (14.5)	1497 (10.0)	0.136	<0.001	0.002	0.002	0.002	0.002	0.001	0.003	0.001	<0.001	0.002	0.002
COPD exacerbation past 12 months)	6221 (27.9)	4637 (31.1)	0.071	0.001	0.004	0.003	0.003	0.007	0.006	0.002	0.005	0.005	0.007	0.008
Former smoking	12240 (54.9)	8941 (60.0)	0.104	0.001	0.003	0.002	0.002	0.005	0.002	0.002	0.002	0.005	0.006	0.006
Ethnicity (%)			0.101	0.002	0.012	0.014	0.013	0.013	0.015	0.004	0.004	0.005	0.005	0.005
White	19575 (87.7)	12891 (86.5)												
South Asian	197 (0.9)	291 (2.0)												

Black	130 (0.6)	138 (0.9)											
Mixed	49 (0.2)	44 (0.3)											
Unknown	2357 (10.6)	1541 (10.3)											
**BMI category (%)**			0.038	0.001	0.004	0.004	0.005	0.006	0.005	0.003	0.003	0.001	0.003	0.002
Underweight (<18.5)	969 (4.3)	619 (4.2)											
Normal (18.5-24.9)	6923 (31.0)	4628 (31.0)											
Overweight (25-29.9)	7167 (32.1)	5021 (33.7)											
Obese (>=30)	7249 (32.5)	4637 (31.1)											

Supplementary Table 10 Summary of weights before and after trimming the propensity scores to the region of common support

Cohort	Outcome	Number of HDPS covariates	Median weight pre-trimming (ICS)	Weight range pre-trimming (ICS)	Median weight pre-trimming (LABA/LAMA)	Weight range pre-trimming (LABA/LAMA)	Median weight after trimming (ICS)	Weight range after trimming (ICS)	Median weight after trimming (LABA/LAMA)	Weight range after trimming (LABA/LAMA)	N before trimming	N after trimming	P (trimmed)
**No triple**	Death	0	1.05	0.48 – 3.14	0.93	0.63 – 3.54	1.05	0.48 - 3.14	0.93	0.70 - 3.54	37225	37213	3.22E-04
**With triple**	Death	0	0.98	0.75 – 1.54	0.83	0.53 – 4.37	0.98	0.77 - 1.54	0.83	0.55 - 4.37	78378	78367	1.40E-04
**No triple**	Hospitalisation	0	1.05	0.48 – 3.14	0.93	0.63 – 3.54	1.05	0.48- 3.14	0.93	0.70 - 3.54	37225	37213	3.22E-04
**With triple**	Hospitalisation	0	0.98	0.75 – 1.54	0.83	0.53 – 4.37	0.98	0.77 - 1.54	0.83	0.55 - 4.37	78378	78367	1.40E-04
**No triple**	Death	100	0.98	0.40 - 3.79	0.93	0.60 – 7.90	0.98	0.44 - 3.79	0.93	0.67 - 7.90	37212	37153	0.00159
**With triple**	Death	100	0.95	0.72 -2.49	0.80	0.39 – 12.28	0.95	0.73 - 2.49	0.80	0.41 - 12.28	78336	78270	8.43E-04
**No triple**	Hospitalisation	100	0.96	0.40 – 4.56	0.91	0.60 – 13.58	0.96	0.42 - 4.56	0.91	0.66 - 13.58	37212	37180	8.60E-04
**With triple**	Hospitalisation	100	0.95	0.72 – 2.44	0.80	0.38 – 12.90	0.95	0.73 - 2.44	0.80	0.42 - 12.90	78336	78286	6.38E-04
**No triple**	Death	250	0.94	0.40 – 20.77	0.92	0.60 – 7.54	0.95	0.44 - 20.77	0.92	0.61 - 7.54	37212	37143	0.00185
**With triple**	Death	250	0.95	0.72 – 2.82	0.80	0.28 – 14.74	0.95	0.73 - 2.82	0.80	0.39 - 14.74	78336	78268	8.68E-04
**No triple**	Hospitalisation	250	0.94	0.40 – 15.48	0.91	0.60 – 15.96	0.94	0.42 - 15.48	0.91	0.62 - 15.96	37212	37115	0.00261
**With triple**	Hospitalisation	250	0.94	0.72 – 3.10	0.78	0.31 – 28.00	0.94	0.72 - 3.10	0.78	0.38 - 28.00	78336	78261	9.57E-04
**No triple**	Death	500	0.93	0.40 – 11.14	0.91	0.60 – 8.82	0.93	0.43 - 11.14	0.91	0.62 - 8.82	37212	37102	0.00296
**With triple**	Death	500	0.94	0.72 – 5.82	0.78	0.28 – 21.28	0.94	0.73 - 5.82	0.78	0.33 - 21.28	78336	78113	0.00285
**No triple**	Hospitalisation	500	0.91	0.40 – 16.84	0.90	0.60 – 16.73	0.91	0.42 - 16.84	0.90	0.62 - 16.73	37212	37083	0.00347
**With triple**	Hospitalisation	500	0.94	0.72 – 6.81	0.77	0.28 – 26.97	0.94	0.72 - 6.81	0.77	0.32 - 26.97	78336	78203	0.00170
**No triple**	Death	750	0.91	0.40 – 16.42	0.91	0.60 – 19.89	0.91	0.41 - 16.42	0.91	0.61 - 19.89	37212	37123	0.00239
**With triple**	Death	750	0.93	0.72 – 4.97	0.77	0.28 – 21.77	0.93	0.72 - 4.97	0.77	0.33 - 21.77	78336	78050	0.00365
**No triple**	Hospitalisation	750	0.90	0.40 – 11.31	0.90	0.60 – 28.56	0.90	0.41 - 11.31	0.90	0.62 - 28.56	37212	37059	0.00411
**With triple**	Hospitalisation	750	0.93	0.72 – 6.45	0.76	0.28 – 32.89	0.93	0.72 - 6.45	0.76	0.32 - 32.89	78336	78057	0.00356
**No triple**	Death	1000	0.91	0.40 – 16.22	0.90	0.60 – 26.53	0.91	0.41 - 16.22	0.90	0.61 - 26.53	37212	36958	0.00683
**With triple**	Death	1000	0.93	0.72 – 7.00	0.77	0.28 – 23.51	0.93	0.72 - 7.00	0.77	0.32 - 23.51	78336	77997	0.00433
**No triple**	Hospitalisation	1000	0.89	0.40 – 16.45	0.90	0.60 – 22.40	0.90	0.41 - 16.45	0.90	0.62 - 22.40	37212	37037	0.00470
**With triple**	Hospitalisation	1000	0.93	0.72 – 13.54	0.76	0.28 – 28.83	0.93	0.72 - 13.54	0.76	0.30 - 28.83	78336	77976	0.00460

Supplementary Table 11 Results of Cox regression adjusted using prespecified covariates, with missing ethnicity values handled via multiple imputation.

Outcome	Cohort	HR	Lower 95% CI limit	Upper 95% CI limit
**COVID Hospitalisation**	With triple therapy	1.467682	1.19731	1.799109
**COVID Death**	With triple therapy	1.422373	1.078	1.87676
**COVID Hospitalisation**	No triple therapy	1.204541	0.928609	1.562465
**COVID Death**	No triple therapy	1.243308	0.877607	1.7614

2. Supplementary figures

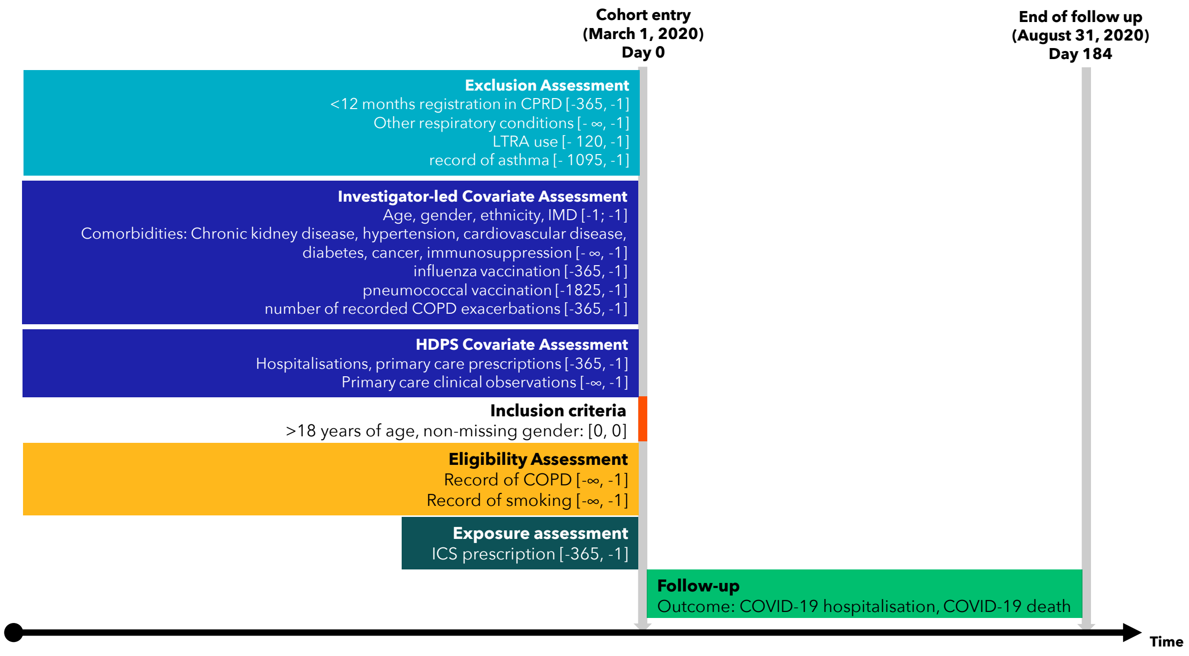

Supplementary Figure 1 Study diagram

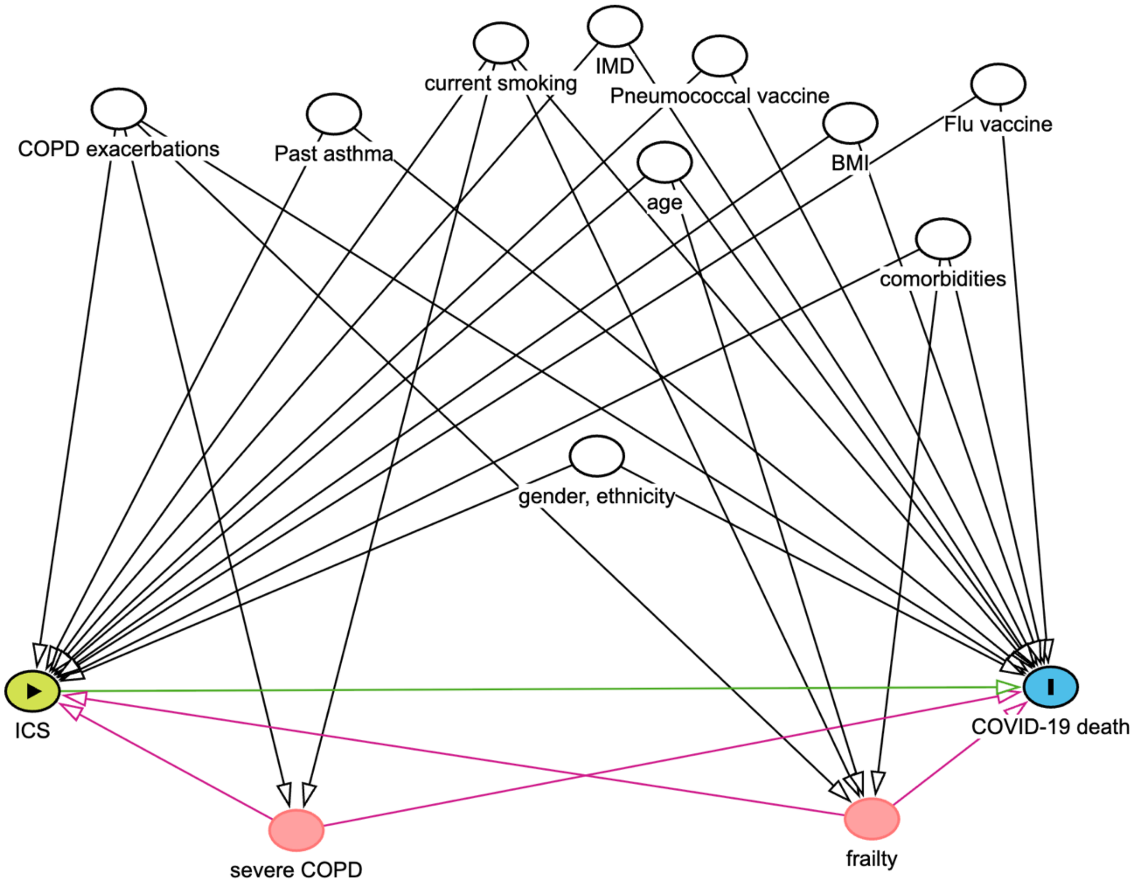

Supplementary Figure 2 Directed Acyclic Graph (DAG) depicting the assumed causal relationships underlying the analysis of inhaled corticosteroid (ICS) use and COVID-19 outcomes.

2.1. Concept plots for outcome COVID-19 hospitalisation, including triple therapy users

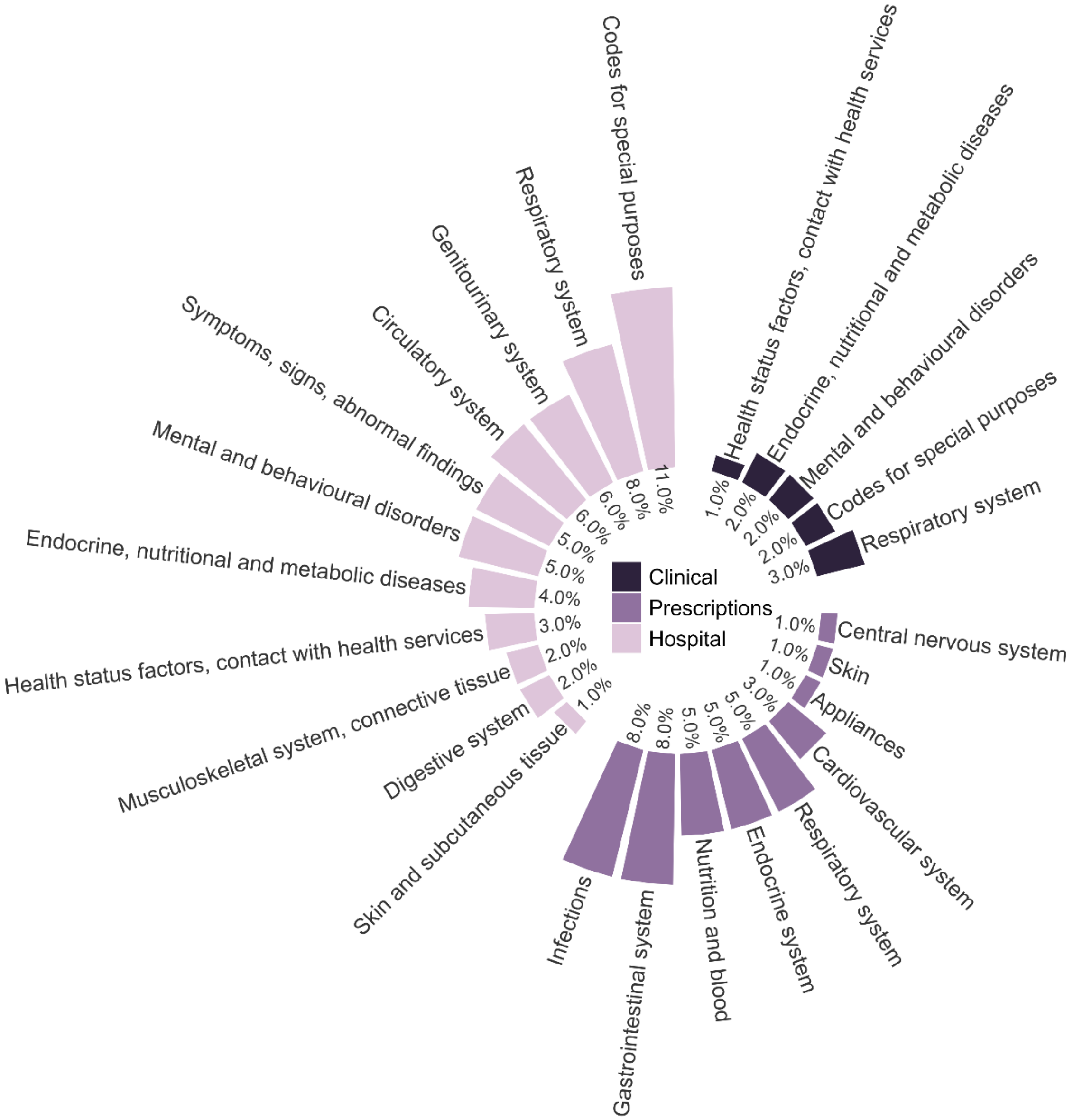

Supplementary Figure 3 Summary of high-level concepts captured in the top 100 ranked high-dimensional propensity score covariates by data dimension for COVID-19 hospitalisations, including triple therapy users

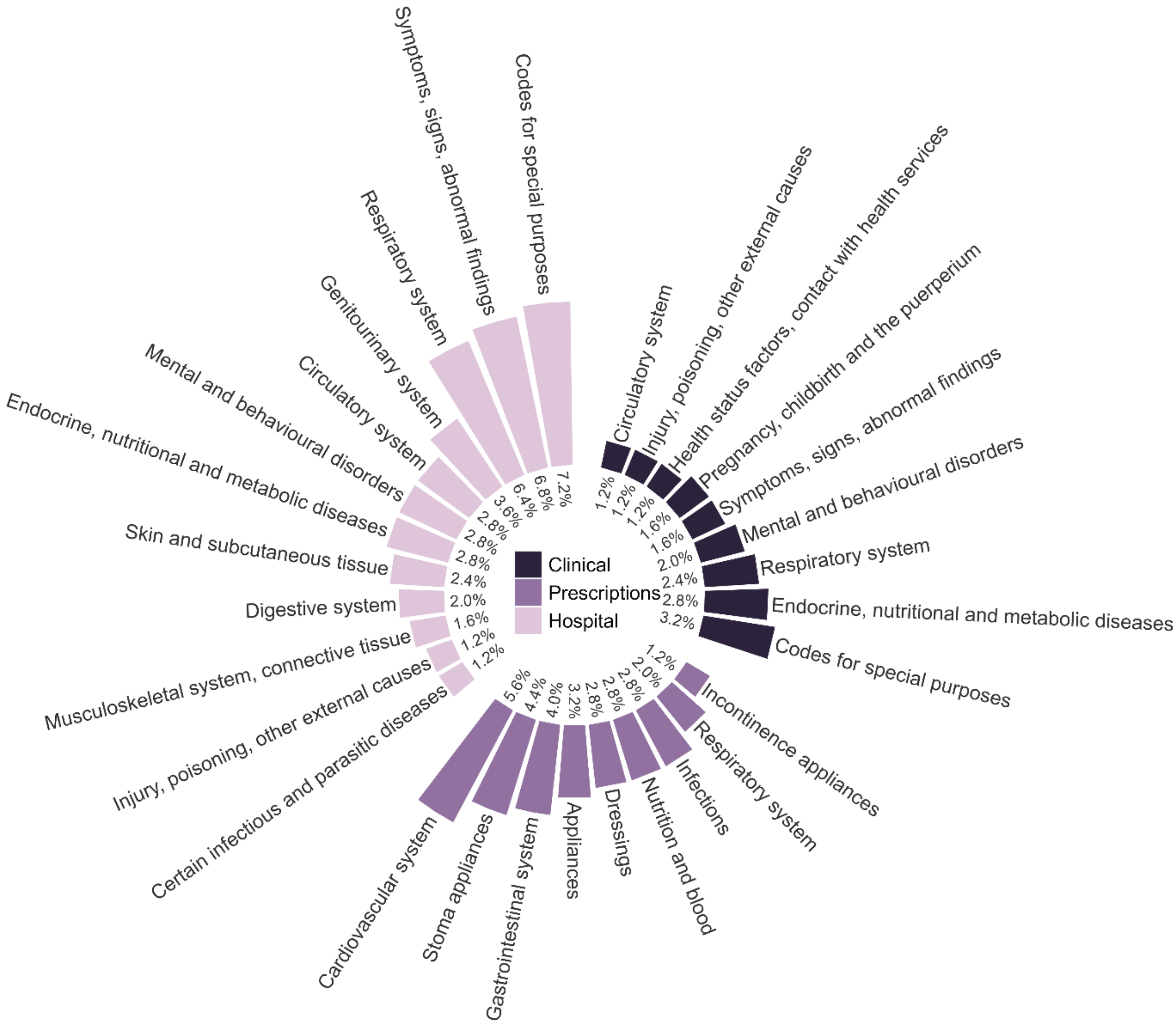

Supplementary Figure 4 Summary of high-level concepts captured in the top 250 ranked high-dimensional propensity score covariates by data dimension for COVID-19 hospitalisations, including triple therapy users

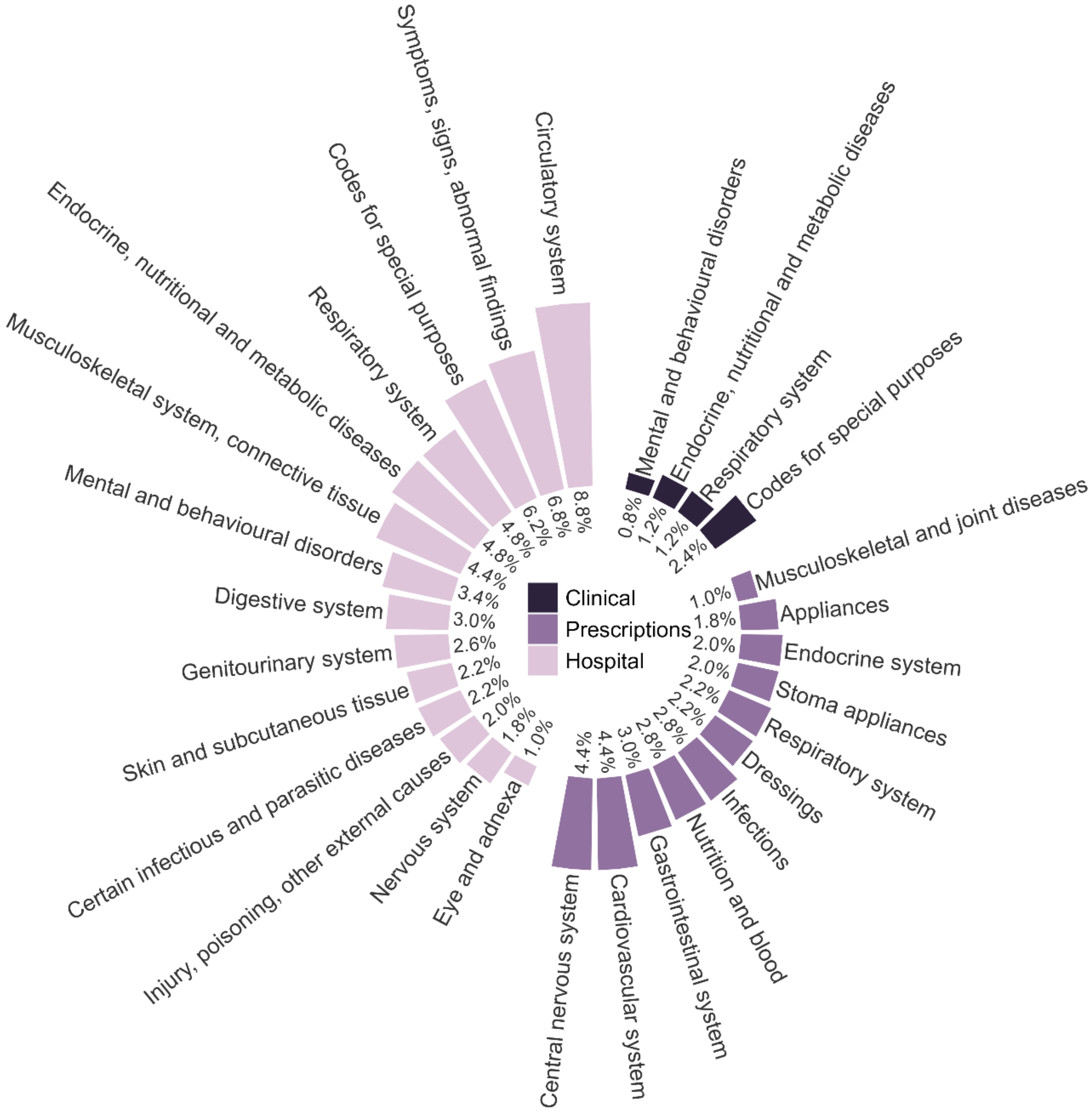

Supplementary Figure 5 Summary of high-level concepts captured in the top 500 ranked high-dimensional propensity score covariates by data dimension for COVID-19 hospitalisations, including triple therapy users

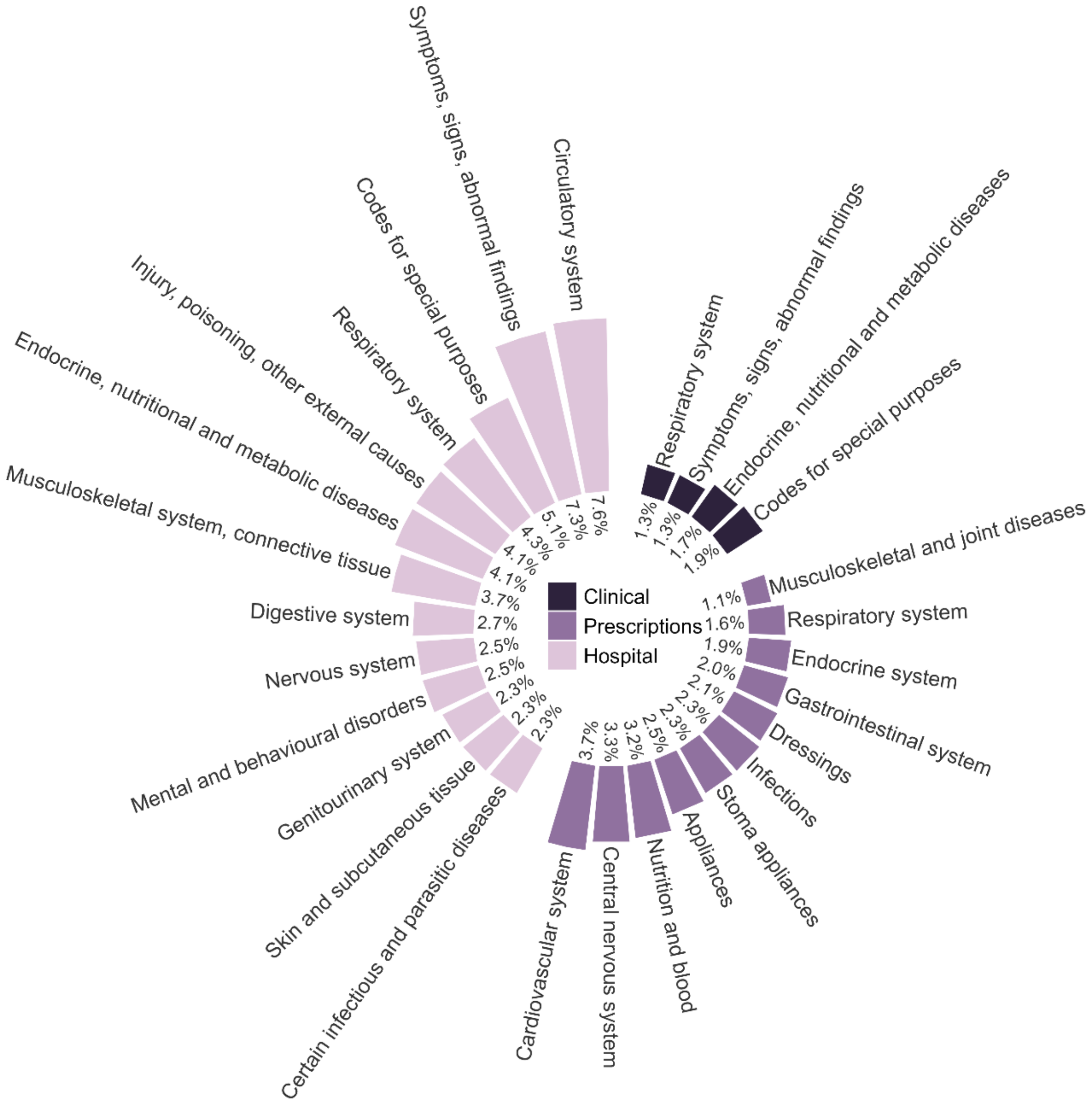

Supplementary Figure 6 Summary of high-level concepts captured in the top 750 ranked high-dimensional propensity score covariates by data dimension for COVID-19 hospitalisations, including triple therapy users

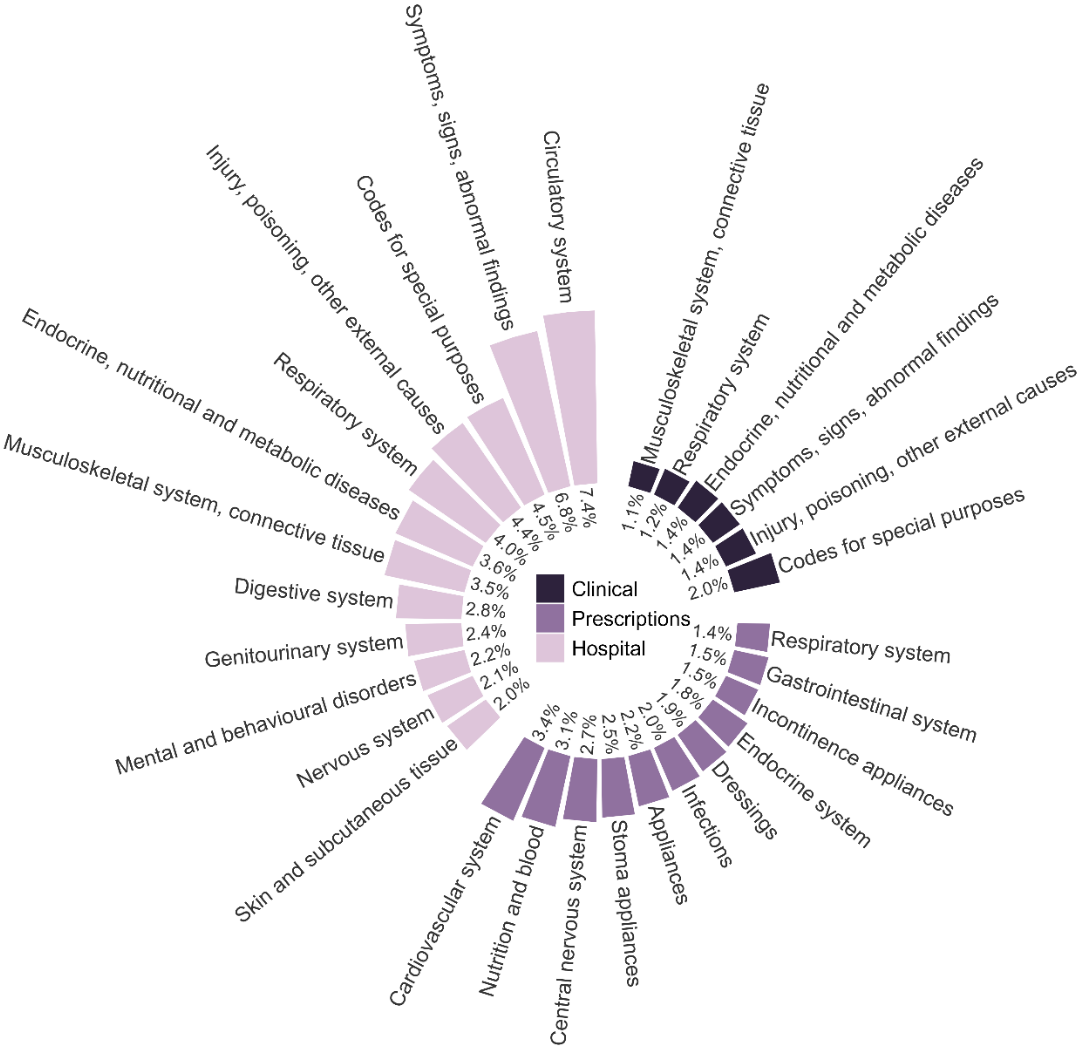

Supplementary Figure 7 Summary of high-level concepts captured in the top 1000 ranked high-dimensional propensity score covariates by data dimension for COVID-19 hospitalisations, including triple therapy users

2.2. Concept plots for outcome COVID-19 hospitalisation, excluding triple therapy users

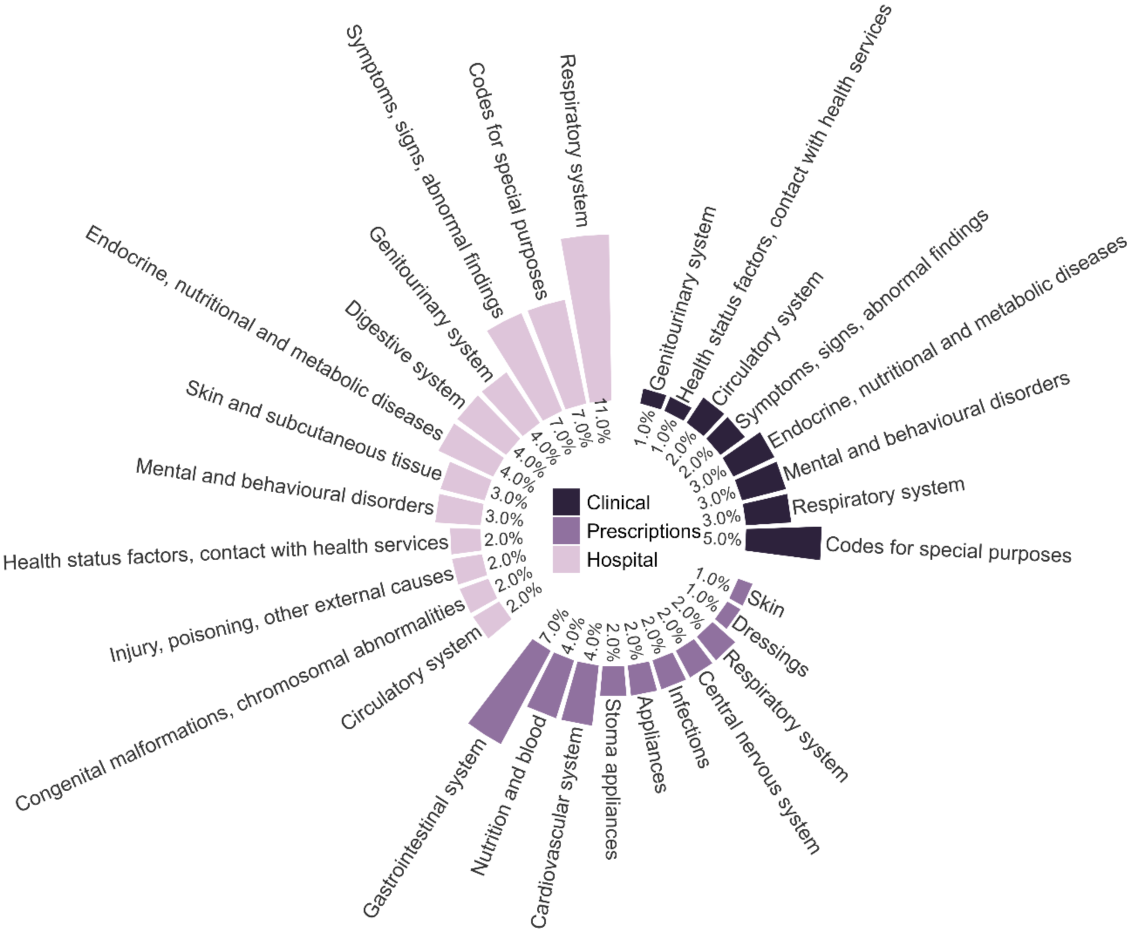

Supplementary Figure 8 Summary of high-level concepts captured in the top 100 ranked high-dimensional propensity score covariates by data dimension for COVID-19 hospitalisations, excluding triple therapy users

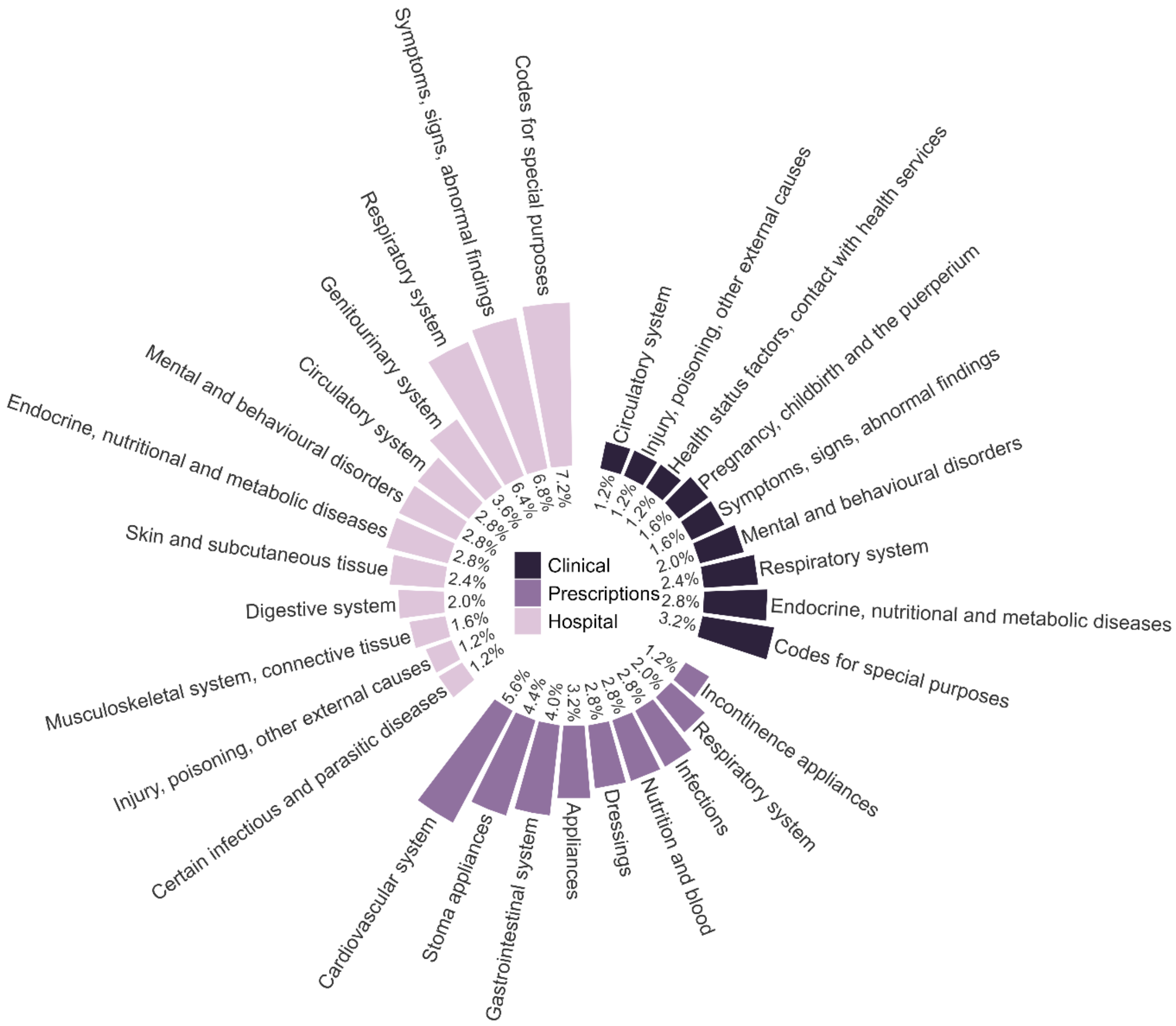

Supplementary Figure 9 Summary of high-level concepts captured in the top 250 ranked high-dimensional propensity score covariates by data dimension for COVID-19 hospitalisations, excluding triple therapy users

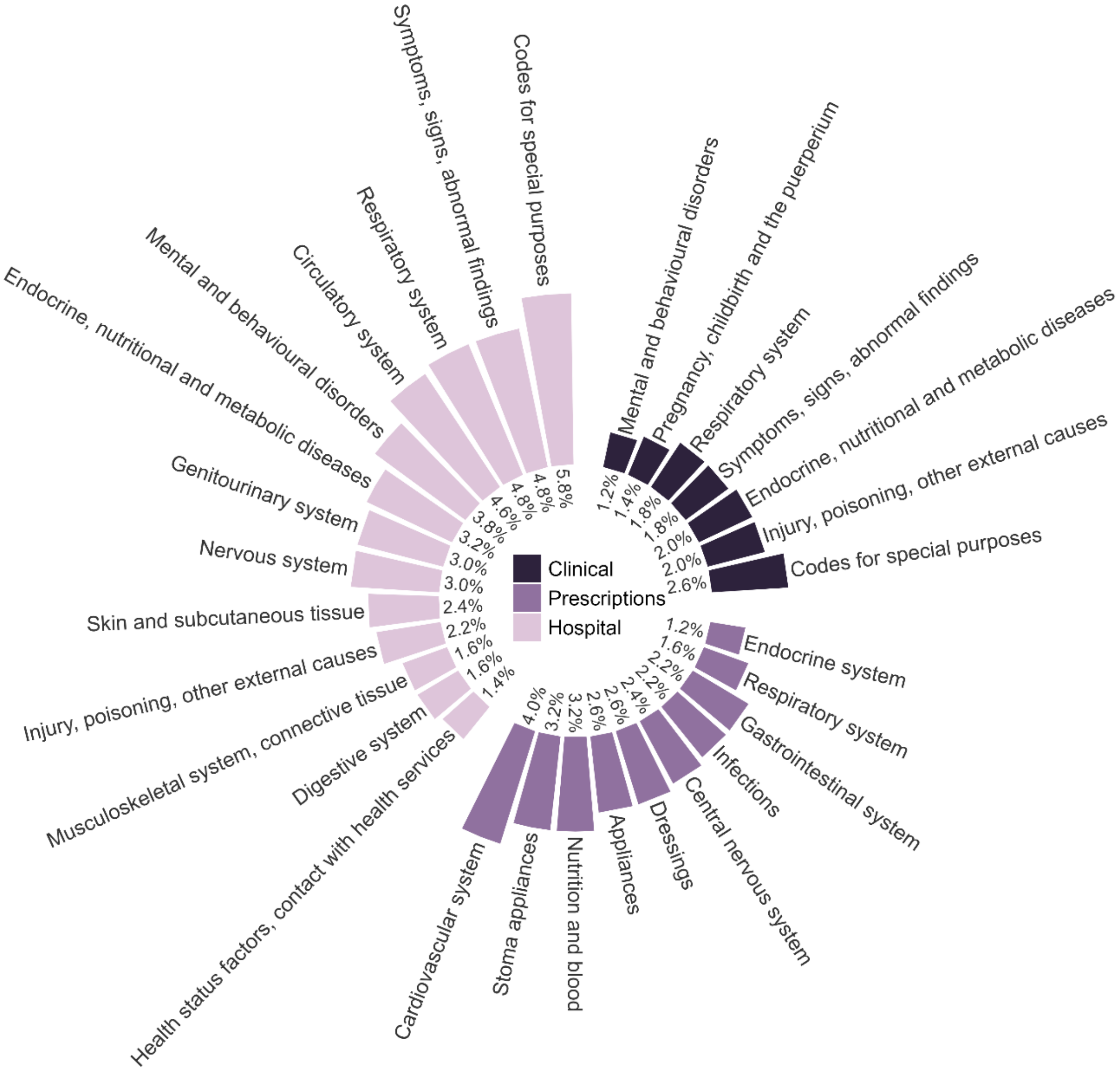

Supplementary Figure 10 Summary of high-level concepts captured in the top 500 ranked high-dimensional propensity score covariates by data dimension for COVID-19 hospitalisations, excluding triple therapy users

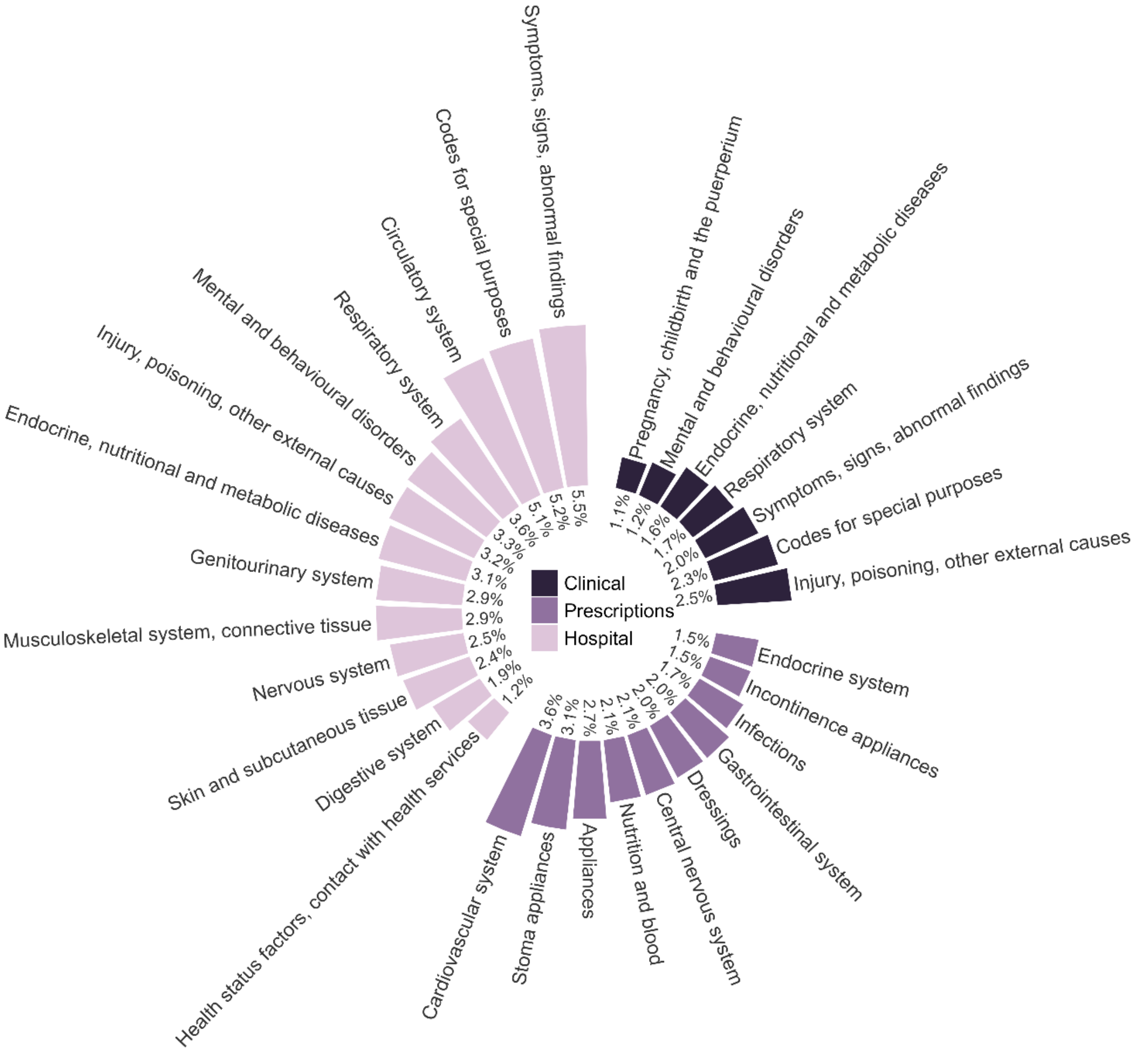

Supplementary Figure 11 Summary of high-level concepts captured in the top 750 ranked high-dimensional propensity score covariates by data dimension for COVID-19 hospitalisations, excluding triple therapy users

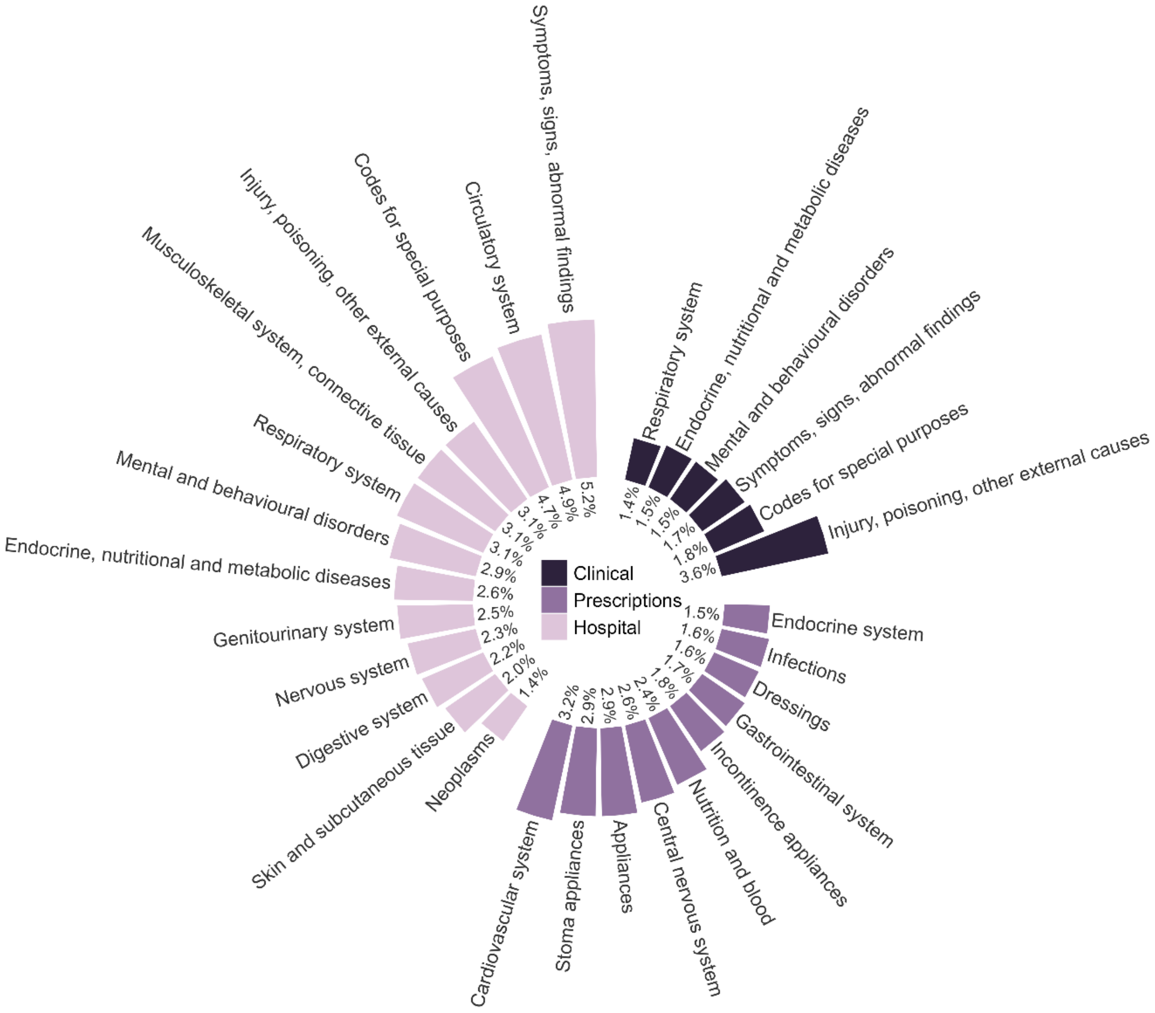

Supplementary Figure 12 Summary of high-level concepts captured in the top 1000 ranked high-dimensional propensity score covariates by data dimension for COVID-19 hospitalisations, excluding triple therapy users

2.3. Concept plots for outcome COVID-19 death, including triple therapy users

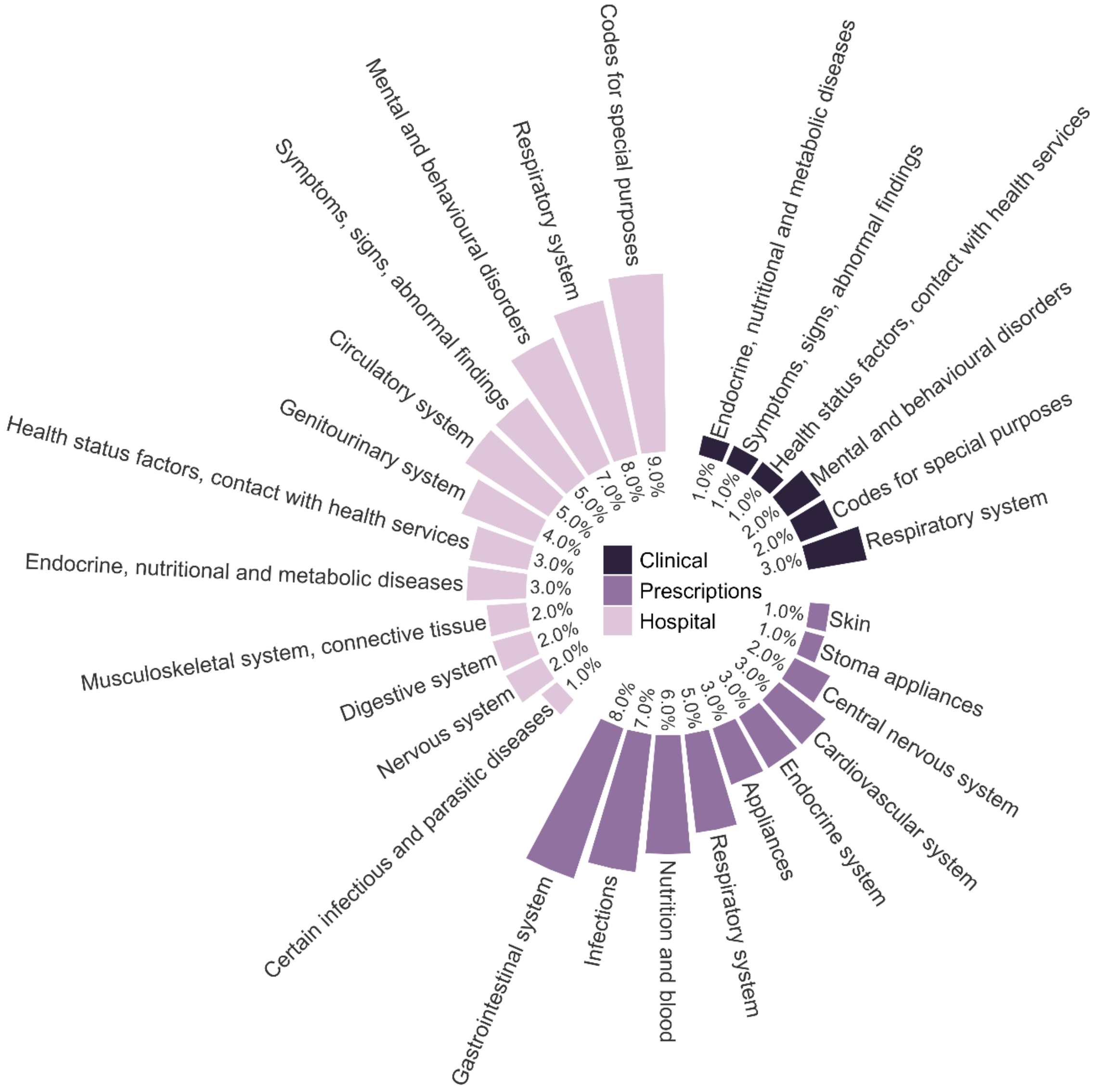

Supplementary Figure 13 Summary of high-level concepts captured in the top 100 ranked high-dimensional propensity score covariates by data dimension for COVID-19 death, including triple therapy users

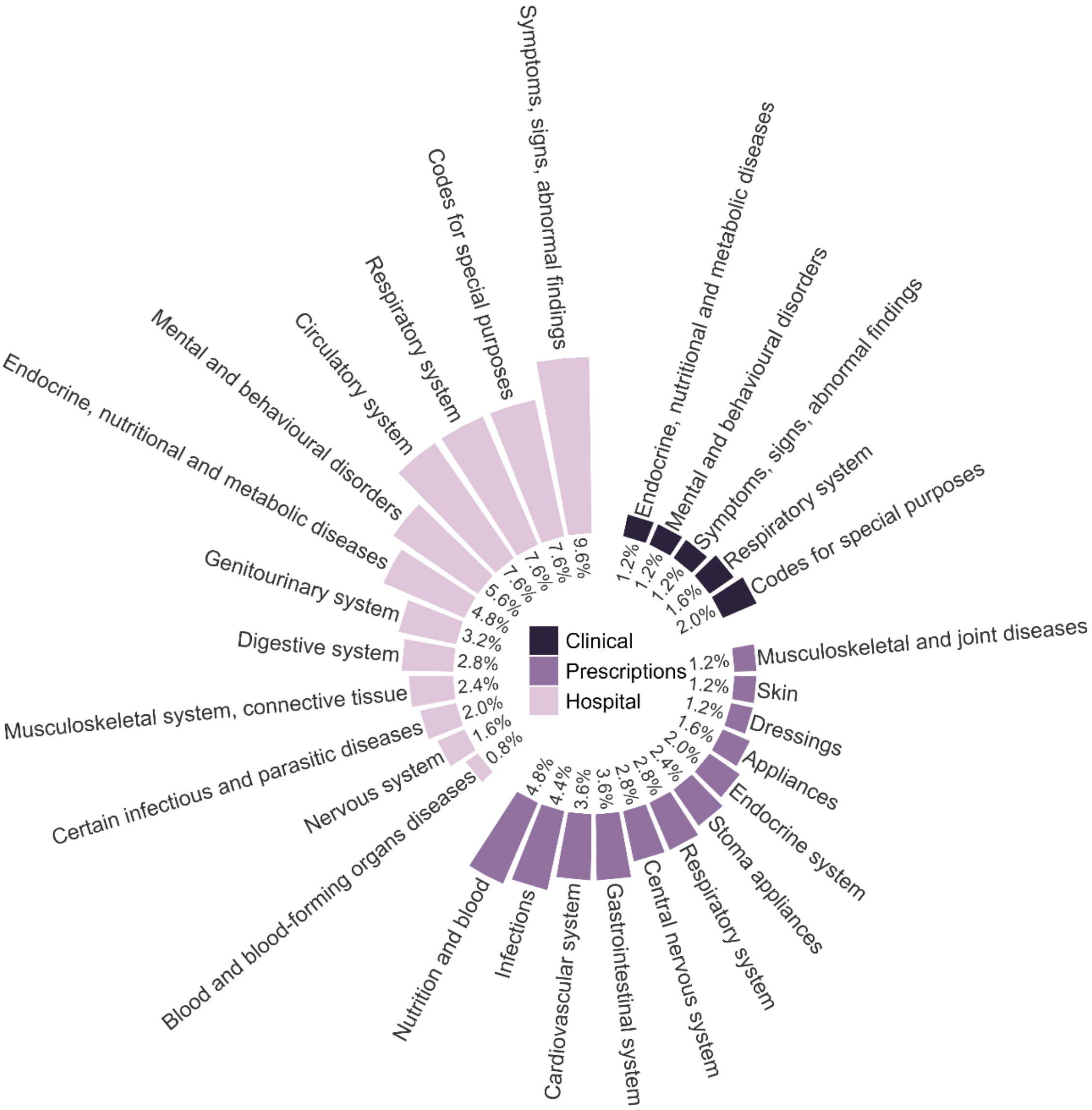

Supplementary Figure 14 Summary of high-level concepts captured in the top 250 ranked high-dimensional propensity score covariates by data dimension for COVID-19 death, including triple therapy users

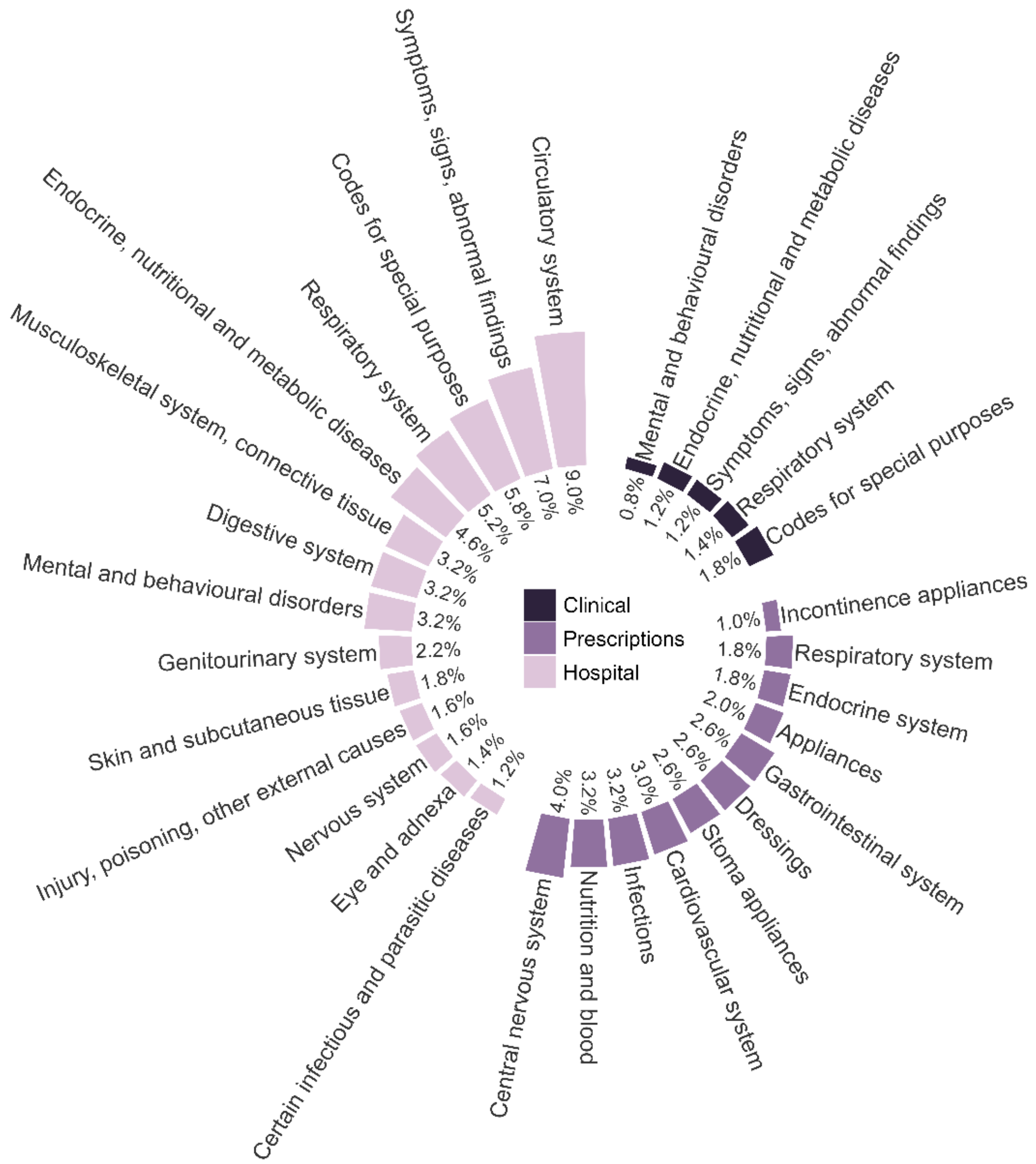

Supplementary Figure 15 Summary of high-level concepts captured in the top 500 ranked high-dimensional propensity score covariates by data dimension for COVID-19 death, including triple therapy users

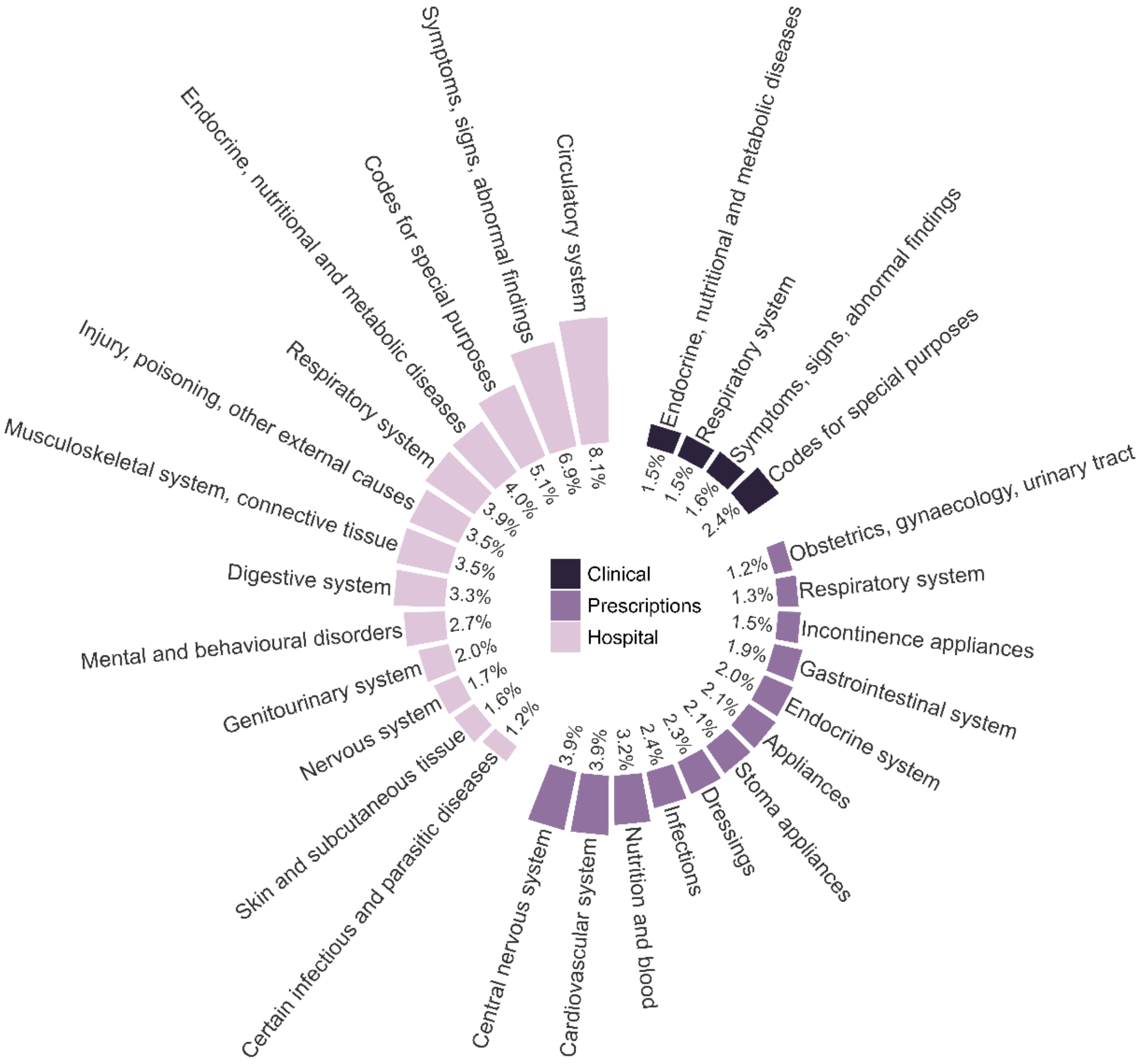

Supplementary Figure 16 Summary of high-level concepts captured in the top 750 ranked high-dimensional propensity score covariates by data dimension for COVID-19 death, including triple therapy users

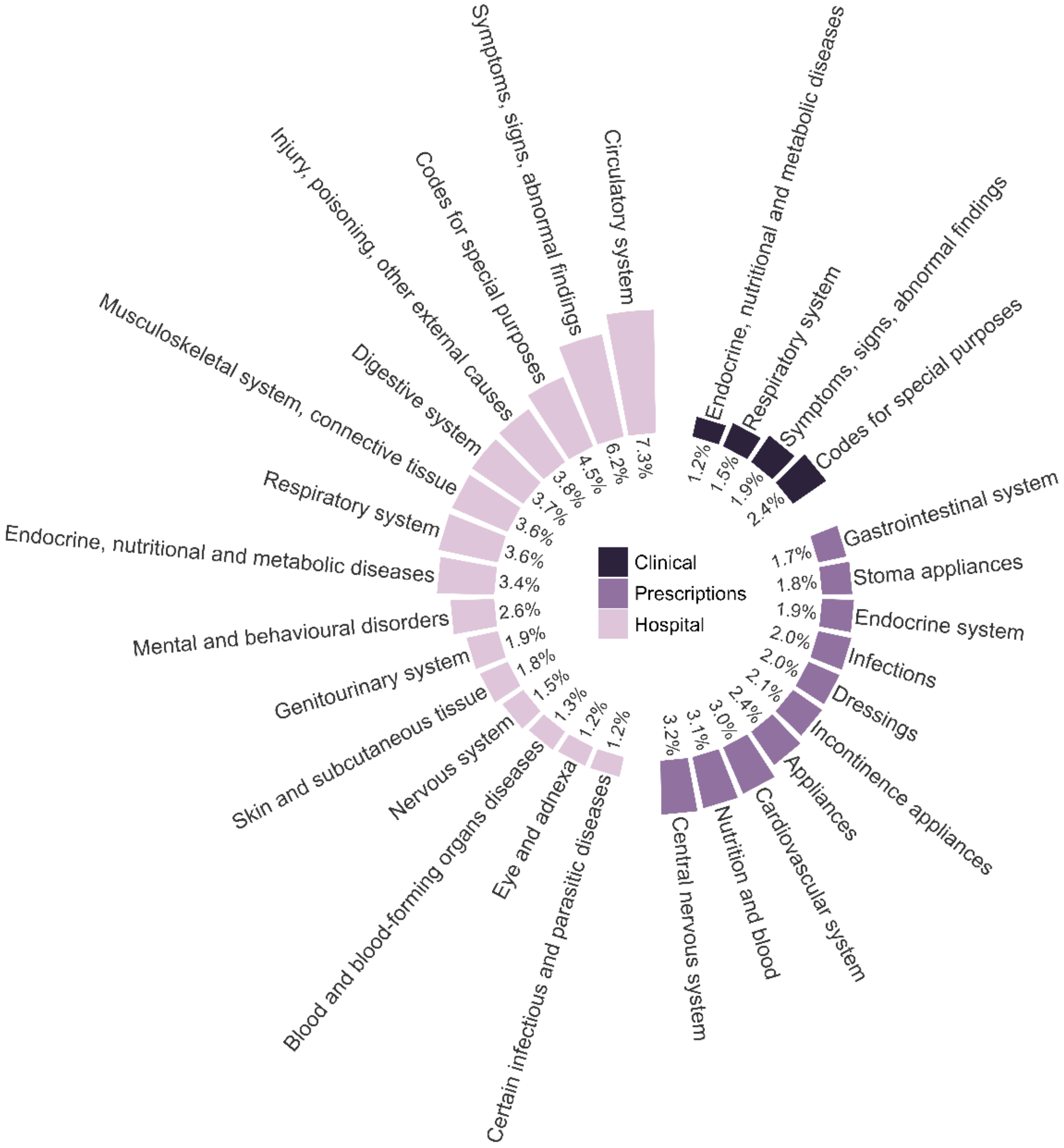

Supplementary Figure 17 Summary of high-level concepts captured in the top 1000 ranked high-dimensional propensity score covariates by data dimension for COVID-19 death, including triple therapy users

2.4. Concept plots for outcome COVID-19 death, excluding triple therapy users

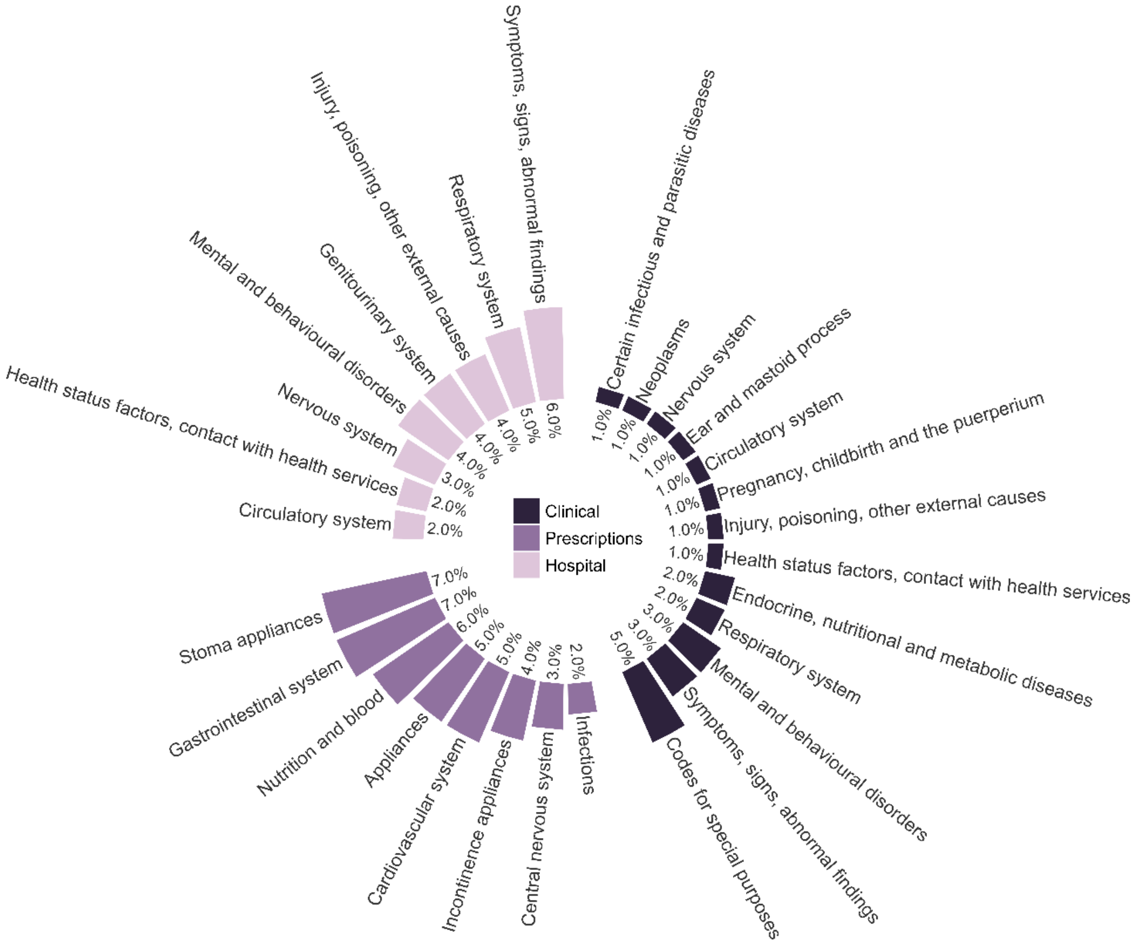

Supplementary Figure 18 Summary of high-level concepts captured in the top 100 ranked high-dimensional propensity score covariates by data dimension for COVID-19 death, excluding triple therapy users

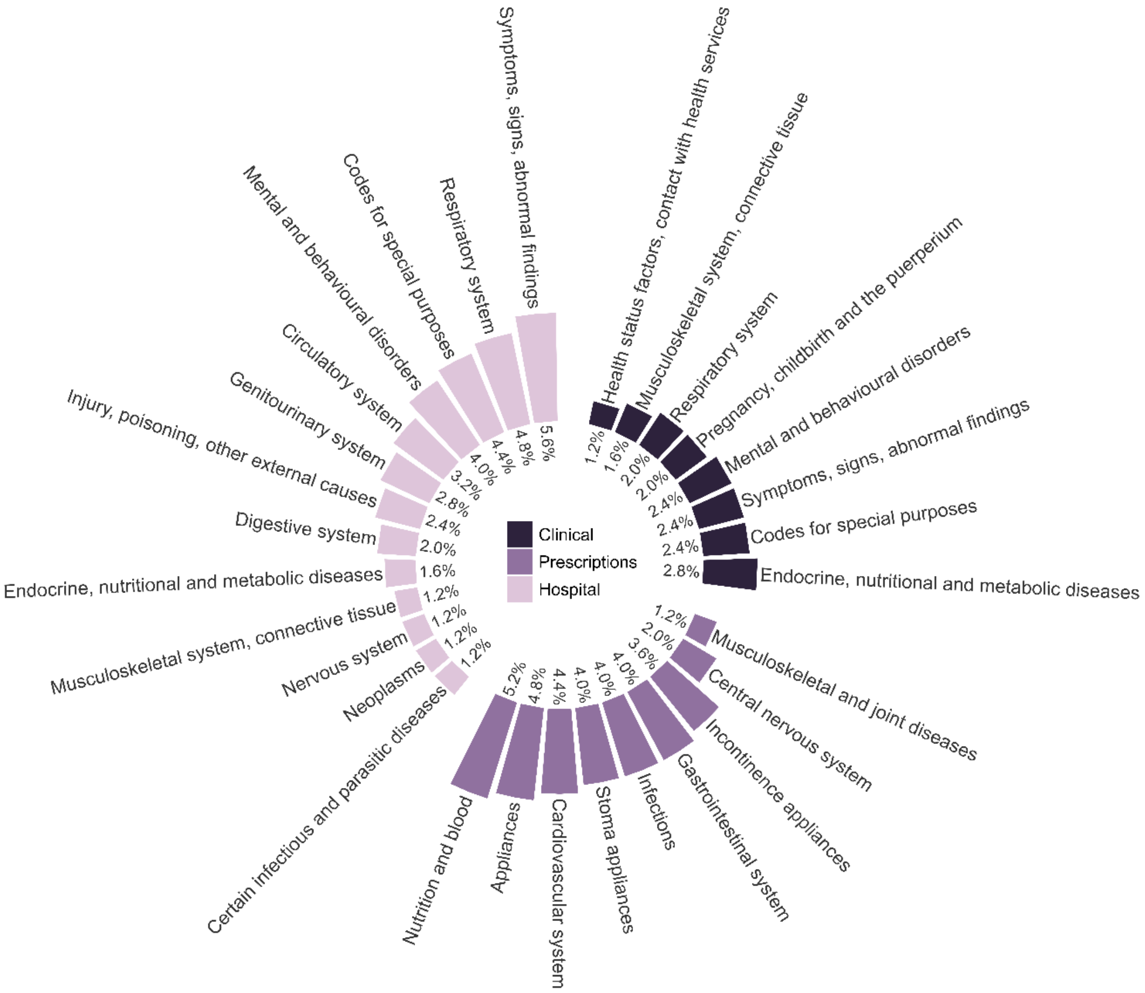

Supplementary Figure 19 Summary of high-level concepts captured in the top 250 ranked high-dimensional propensity score covariates by data dimension for COVID-19 death, excluding triple therapy users

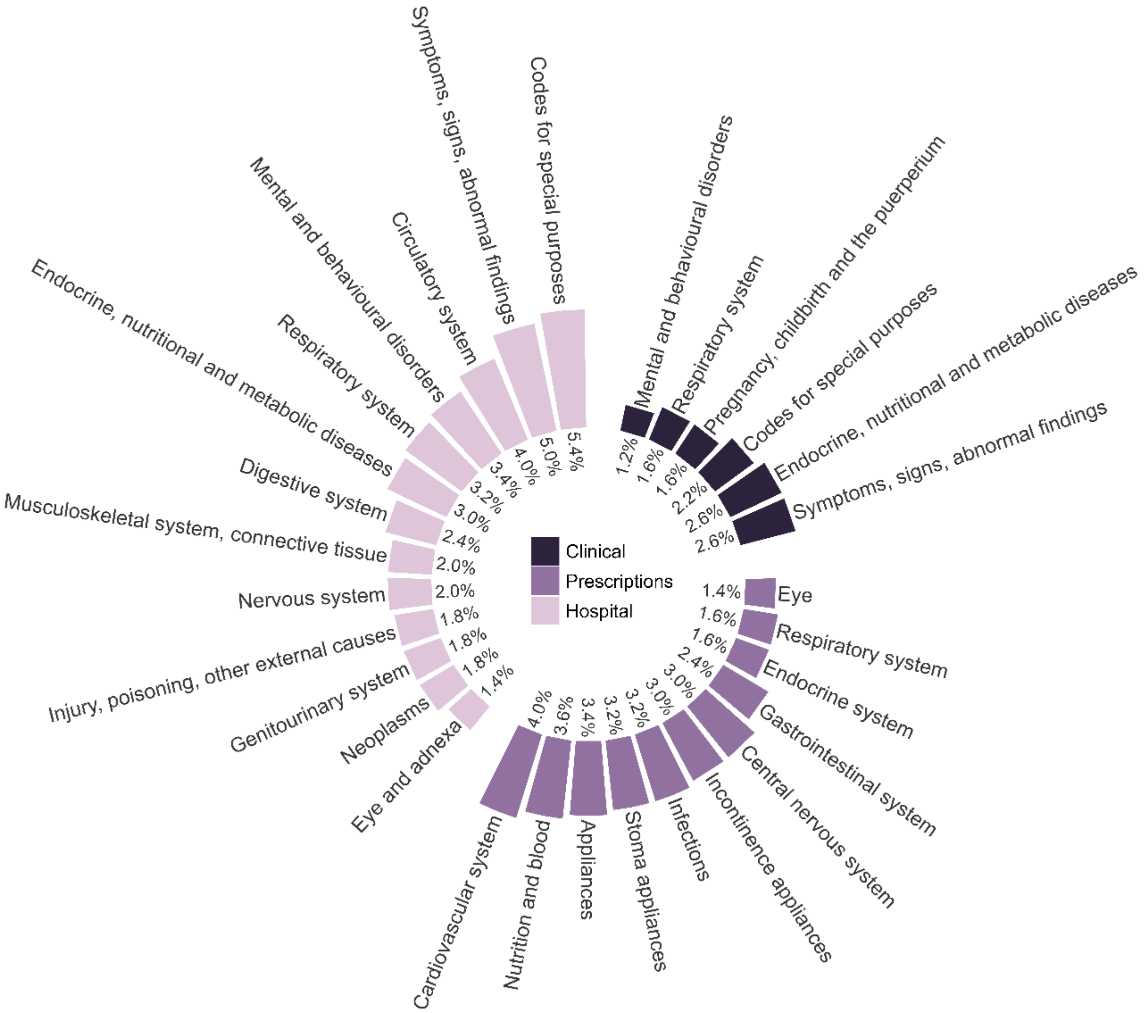

Supplementary Figure 20 Summary of high-level concepts captured in the top 500 ranked high-dimensional propensity score covariates by data dimension for COVID-19 death, including triple therapy users

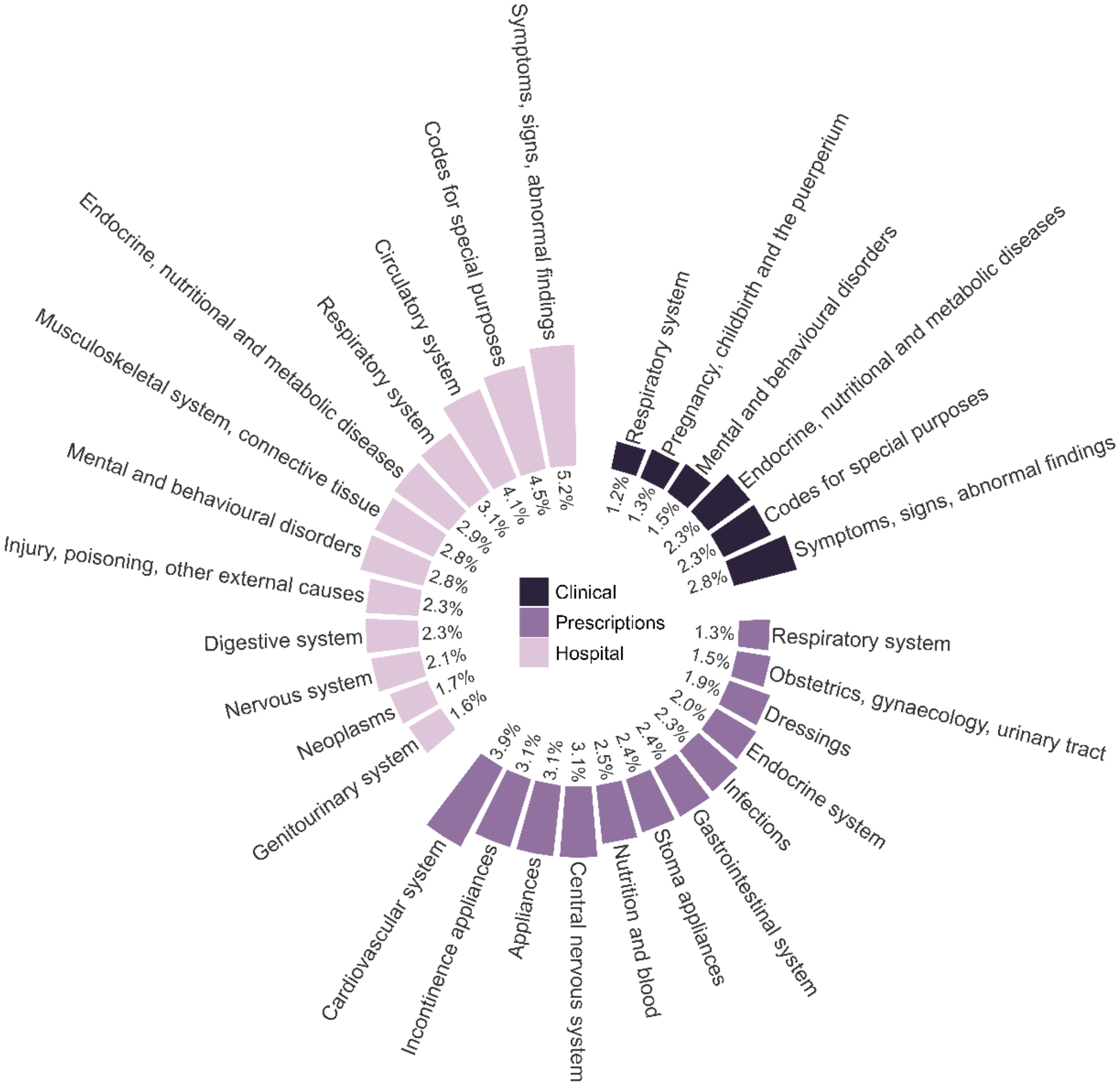

Supplementary Figure 21 Summary of high-level concepts captured in the top 750 ranked high-dimensional propensity score covariates by data dimension for COVID-19 death, excluding triple therapy users

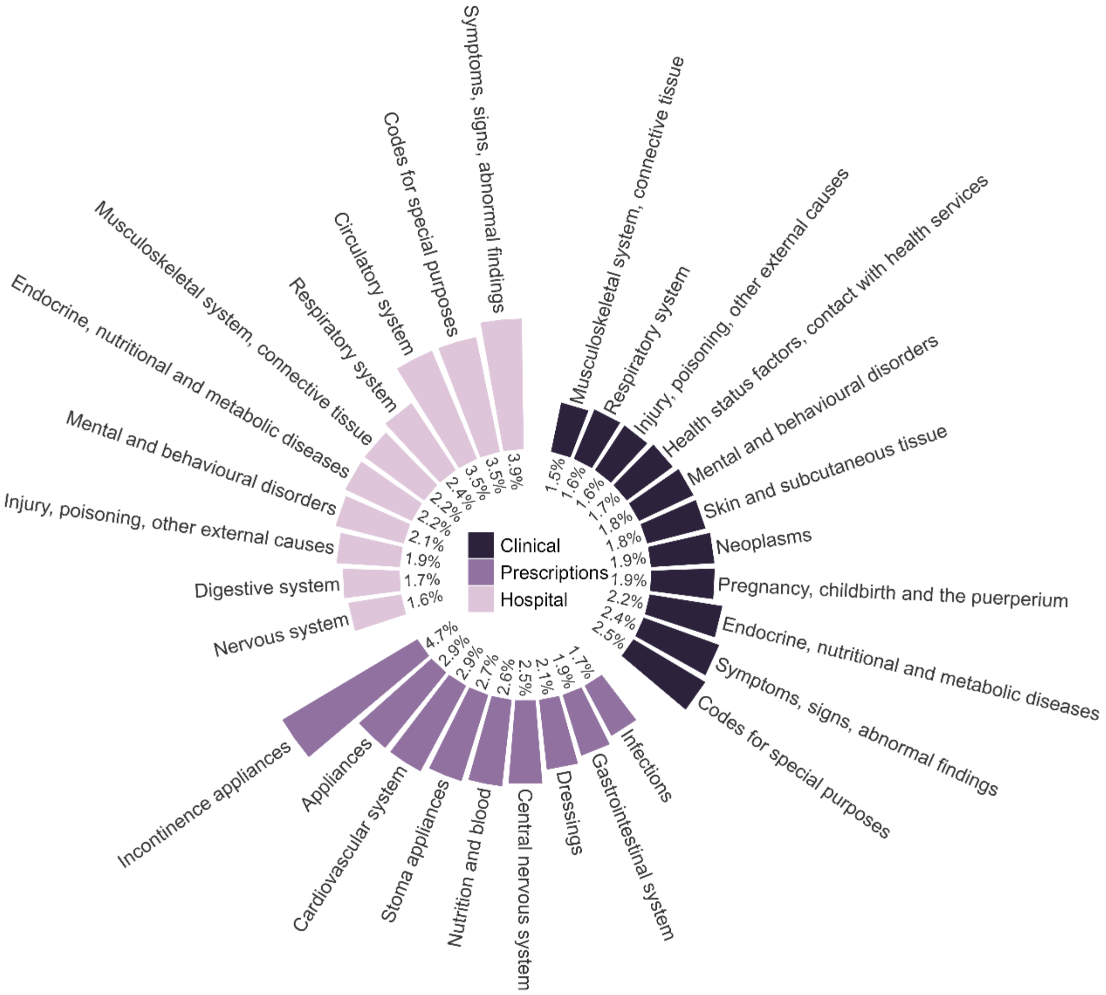

Supplementary Figure 22 Summary of high-level concepts captured in the top 1000 ranked high-dimensional propensity score covariates by data dimension for COVID-19 death, excluding triple therapy users

2.5. Diagnostic plots for HDPS

2.5.1. COVID-19 hospitalisation, including triple therapy users

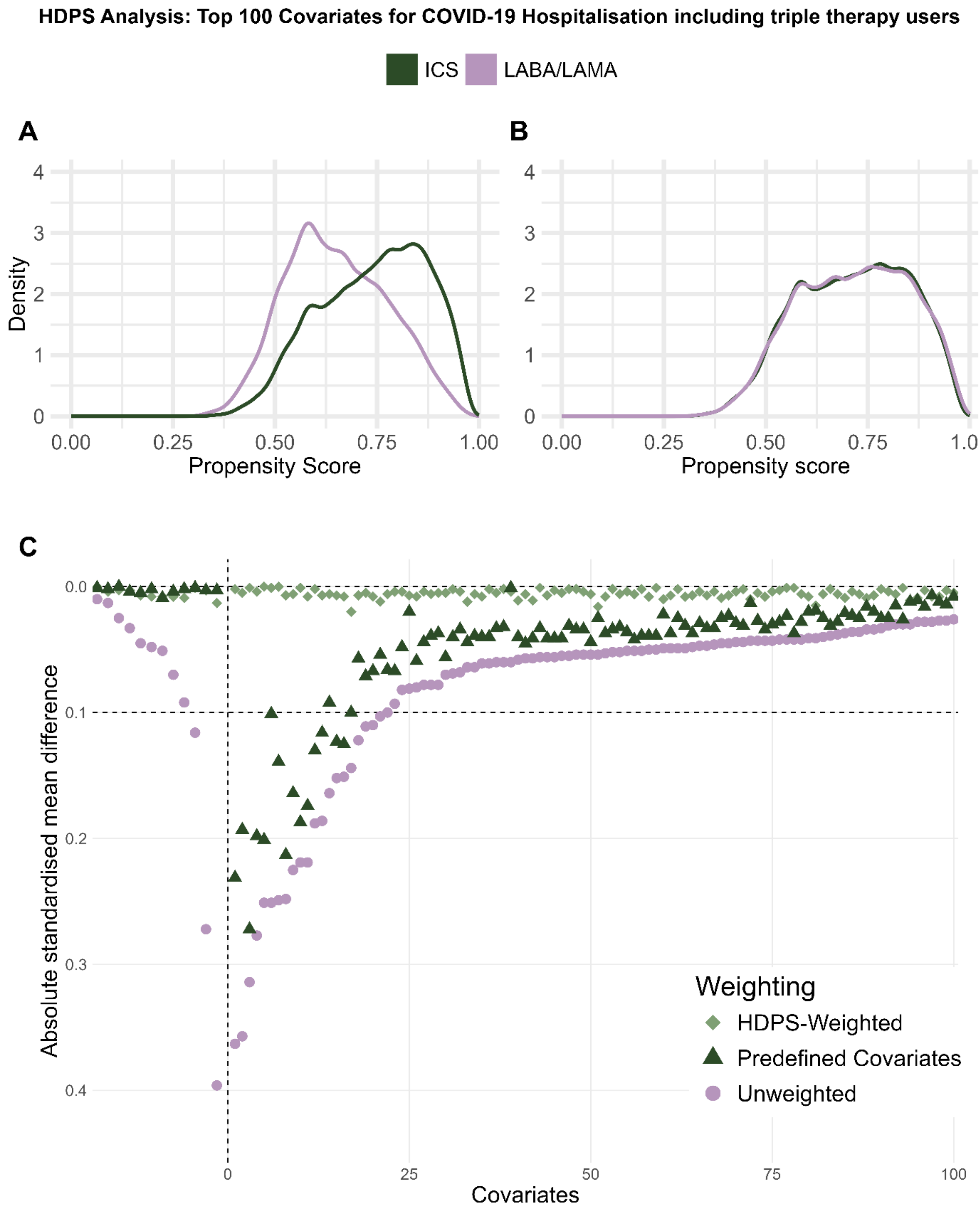

Supplementary Figure 23 Diagnostic plots for high-dimensional propensity score weighted analysis for COVID-19 hospitalisations, including triple therapy users, including the top 100 ranked covariates. A) high-dimensional propensity score (HDPS) distribution, B) weighted HDPS distribution, C) Comparison of absolute standardised differences in the pre-defined and high-dimensional propensity score covariates between unweighted, predefined and HDPS weighted cohort. Points to the left of the x-axis represent predefined covariates, points to the right represent HDPS-identified covariates.

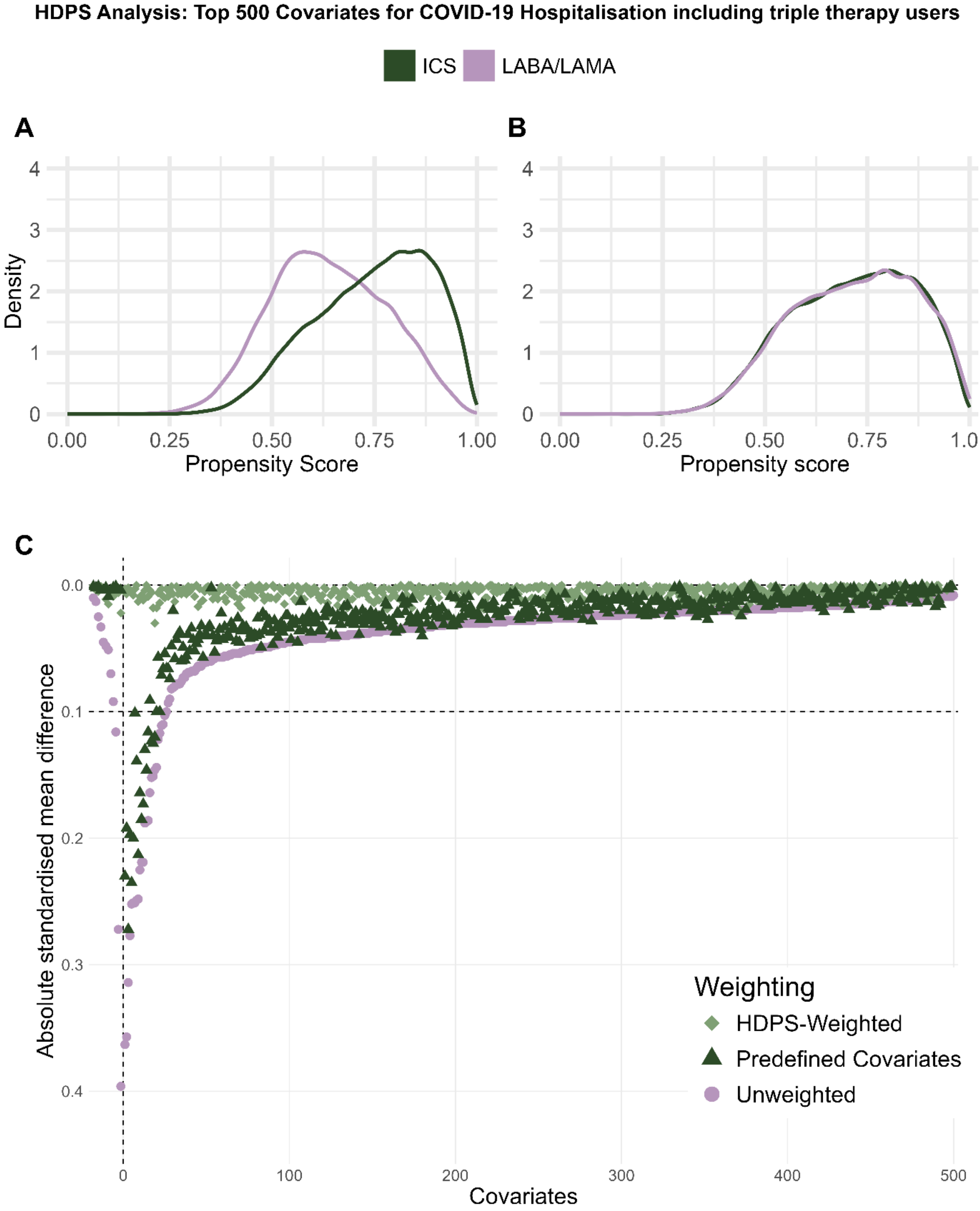

Supplementary Figure 24 Diagnostic plots for high-dimensional propensity score weighted analysis for COVID-19 hospitalisations, including triple therapy users, including the top 500 ranked covariates. A) high-dimensional propensity score (HDPS) distribution, B) weighted HDPS distribution, C) Comparison of absolute standardised differences in the pre-defined and high-dimensional propensity score covariates between unweighted, predefined and HDPS weighted cohort. Points to the left of the x-axis represent predefined covariates, points to the right represent HDPS-identified covariates.

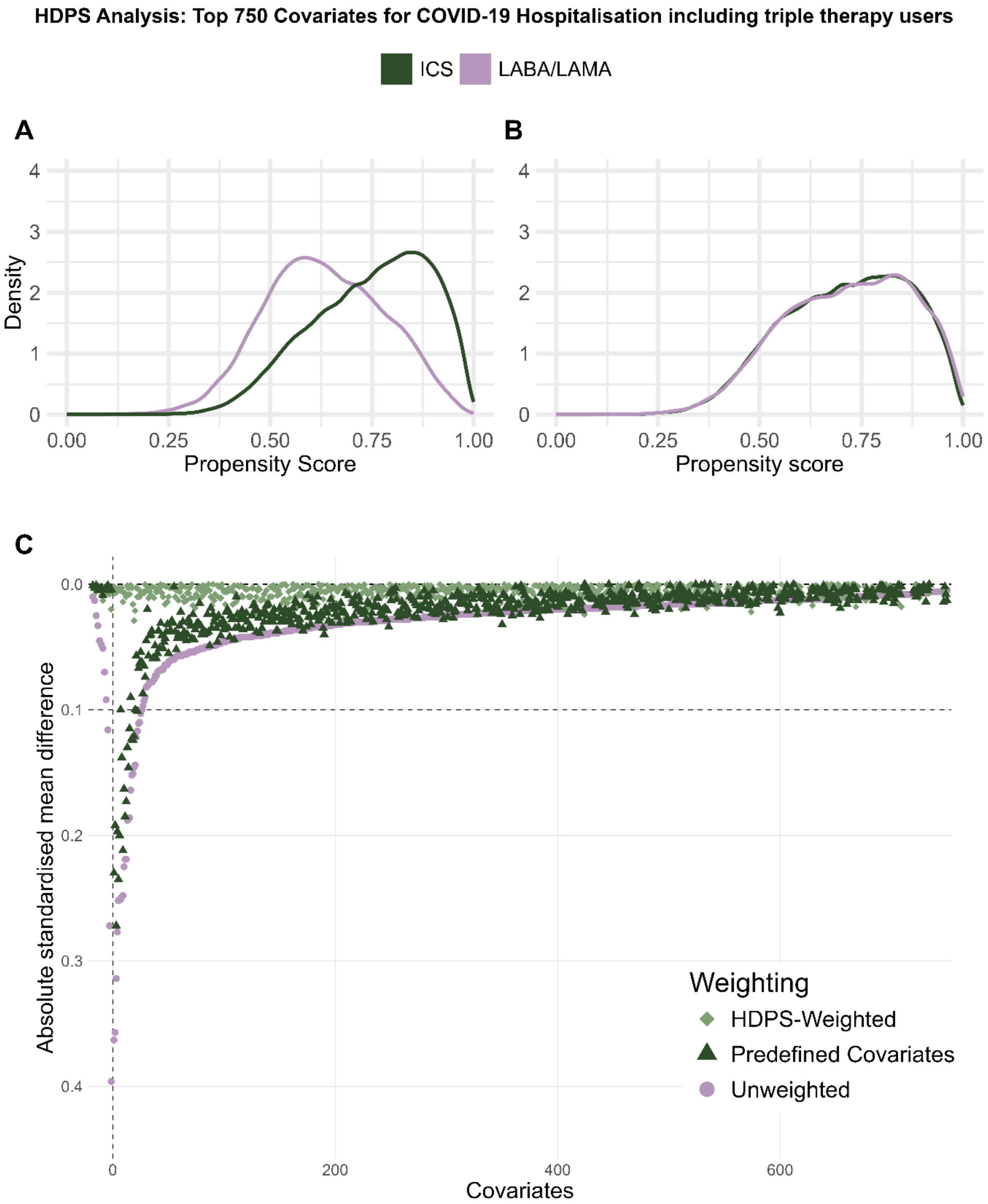

Supplementary Figure 25 Diagnostic plots for high-dimensional propensity score weighted analysis for COVID-19 hospitalisations, including triple therapy users, including the top 750 ranked covariates. A) high-dimensional propensity score (HDPS) distribution, B) weighted HDPS distribution, C) Comparison of absolute standardised differences in the pre-defined and high-dimensional propensity score covariates between unweighted, predefined and HDPS weighted cohort. Points to the left of the x-axis represent predefined covariates, points to the right represent HDPS-identified covariates.

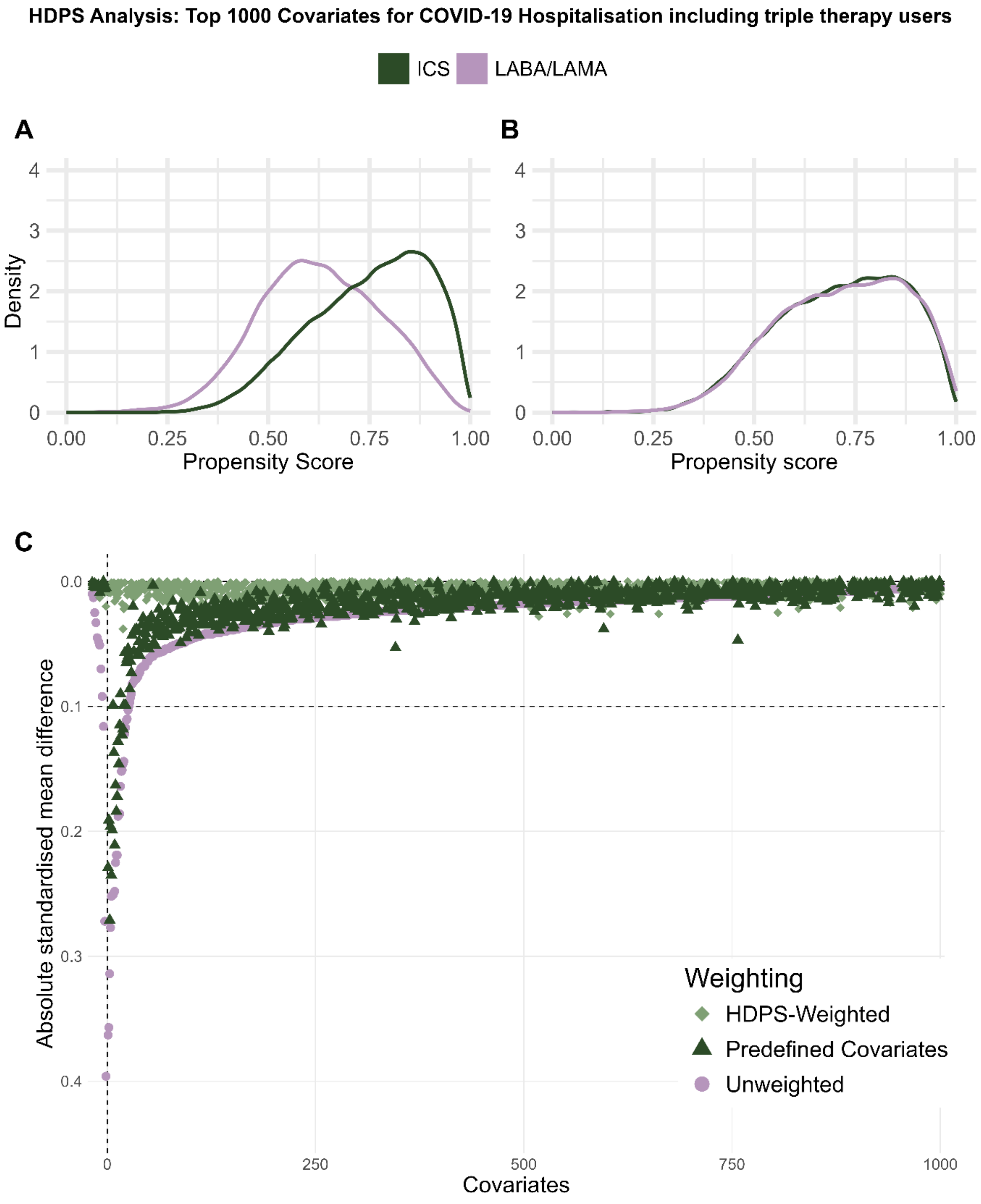

Supplementary Figure 26 Diagnostic plots for high-dimensional propensity score weighted analysis for COVID-19 hospitalisations, including triple therapy users, including the top 1000 ranked covariates. A) high-dimensional propensity score (HDPS) distribution, B) weighted HDPS distribution, C) Comparison of absolute standardised differences in the pre-defined and high-dimensional propensity score covariates between unweighted, predefined and HDPS weighted cohort. Points to the left of the x-axis represent predefined covariates, points to the right represent HDPS-identified covariates.

2.5.2. COVID-19 hospitalisation, excluding triple therapy users

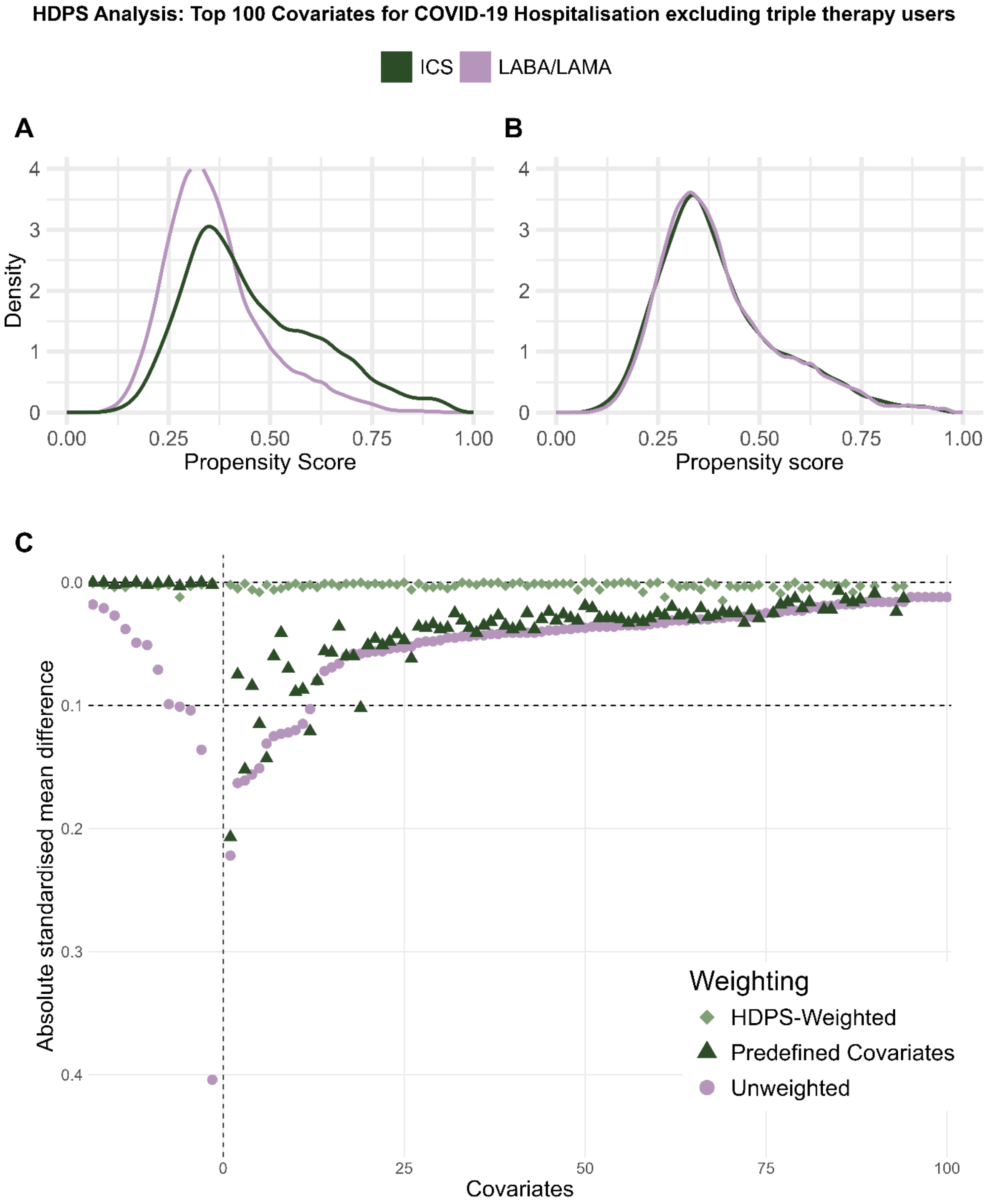

Supplementary Figure 27 Diagnostic plots for high-dimensional propensity score weighted analysis for COVID-19 hospitalisations, excluding triple therapy users, including the top 100 ranked covariates. A) high-dimensional propensity score (HDPS) distribution, B) weighted HDPS distribution, C) Comparison of absolute standardised differences in the pre-defined and high-dimensional propensity score covariates between unweighted, predefined and HDPS weighted cohort. Points to the left of the x-axis represent predefined covariates, points to the right represent HDPS-identified covariates.

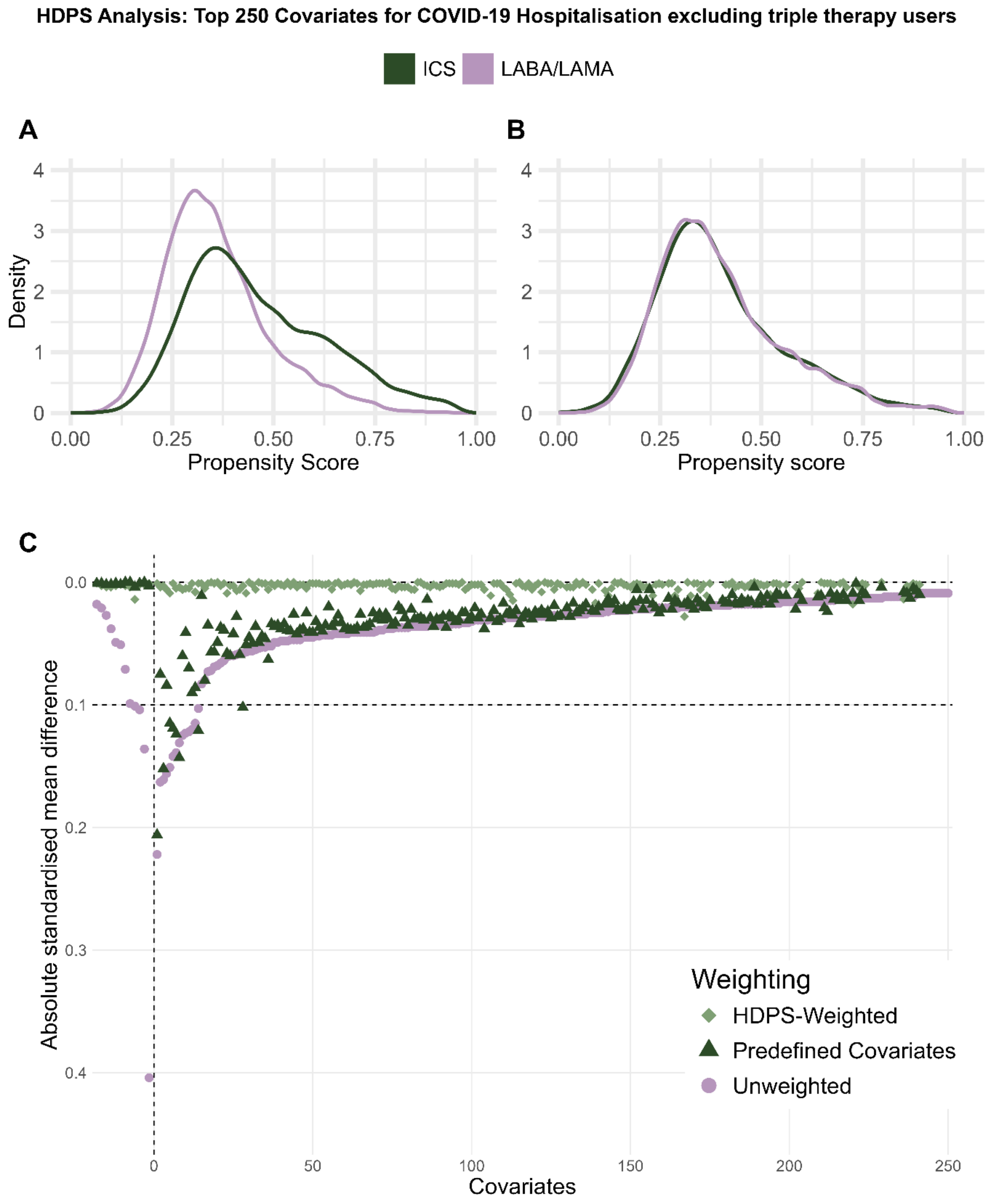

Supplementary Figure 28 Diagnostic plots for high-dimensional propensity score weighted analysis for COVID-19 hospitalisations, excluding triple therapy users, including the top 250 ranked covariates. A) high-dimensional propensity score (HDPS) distribution, B) weighted HDPS distribution, C) Comparison of absolute standardised differences in the pre-defined and high-dimensional propensity score covariates between unweighted, predefined and HDPS weighted cohort. Points to the left of the x-axis represent predefined covariates, points to the right represent HDPS-identified covariates.

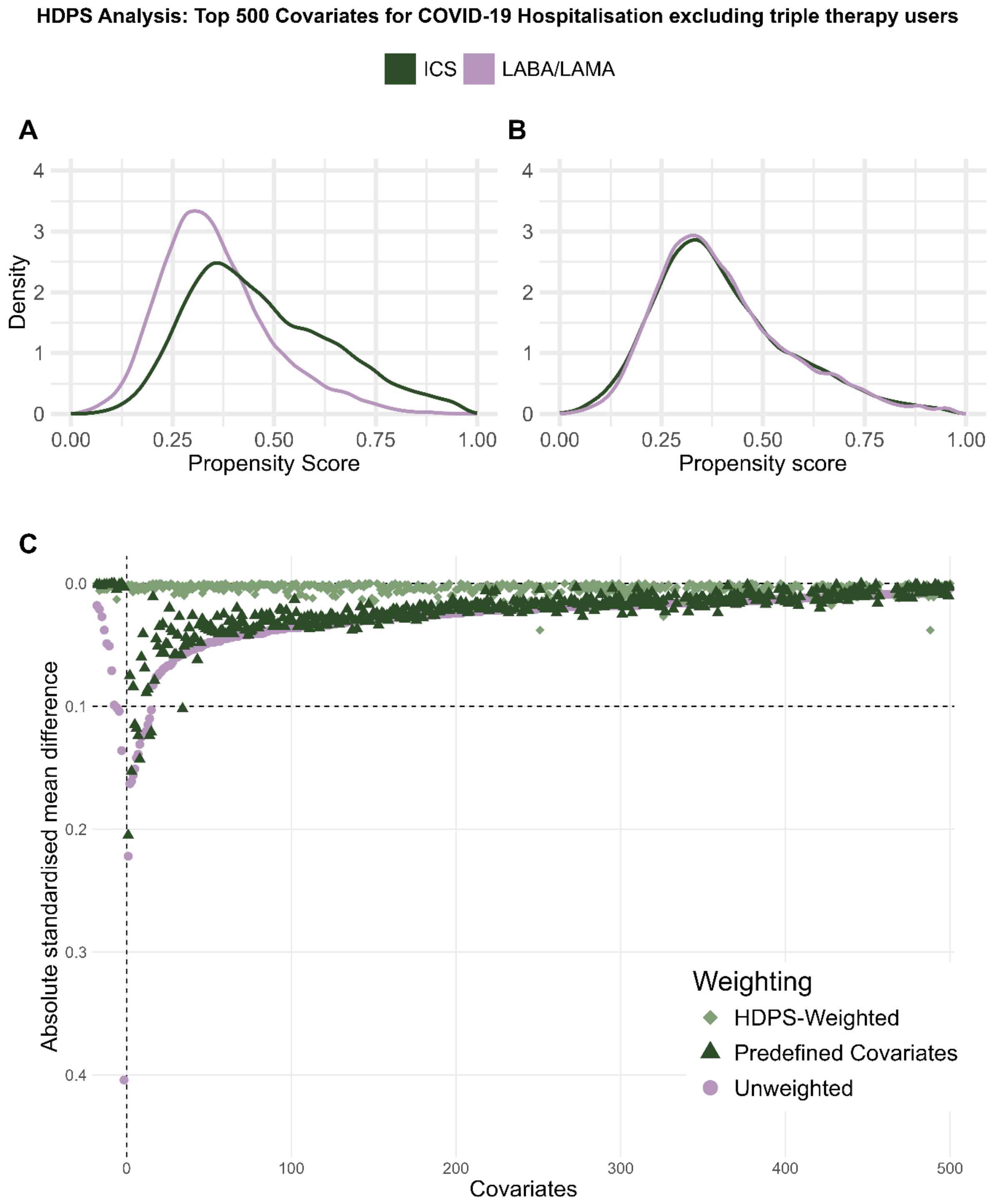

Supplementary Figure 29 Diagnostic plots for high-dimensional propensity score weighted analysis for COVID-19 hospitalisations, excluding triple therapy users, including the top 500 ranked covariates. A) high-dimensional propensity score (HDPS) distribution, B) weighted HDPS distribution, C) Comparison of absolute standardised differences in the pre-defined and high-dimensional propensity score covariates between unweighted, predefined and HDPS weighted cohort. Points to the left of the x-axis represent predefined covariates, points to the right represent HDPS-identified covariates.

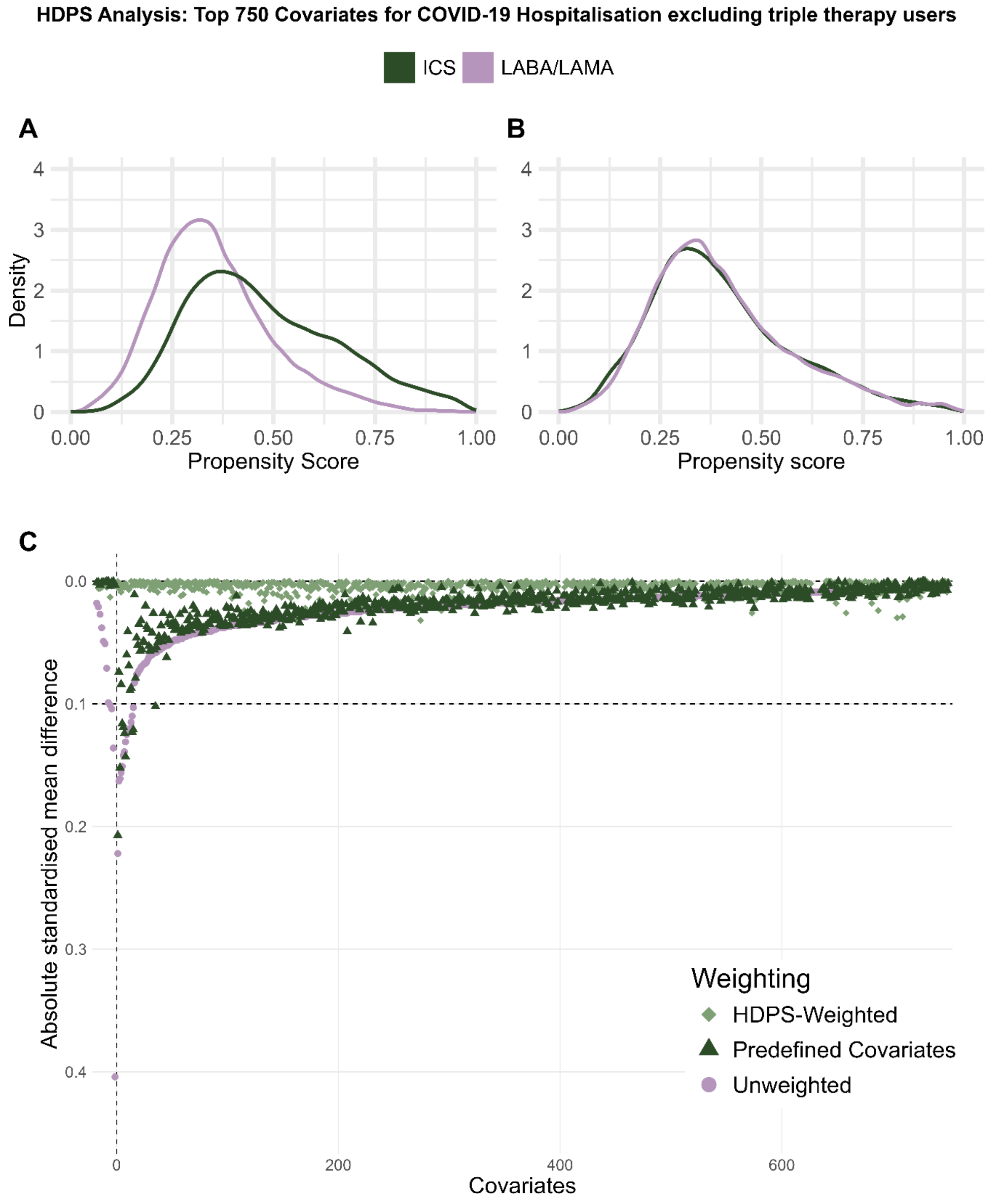

Supplementary Figure 30 Diagnostic plots for high-dimensional propensity score weighted analysis for COVID-19 hospitalisations, excluding triple therapy users, including the top 750 ranked covariates. A) high-dimensional propensity score (HDPS) distribution, B) weighted HDPS distribution, C) Comparison of absolute standardised differences in the pre-defined and high-dimensional propensity score covariates between unweighted, predefined and HDPS weighted cohort. Points to the left of the x-axis represent predefined covariates, points to the right represent HDPS-identified covariates.

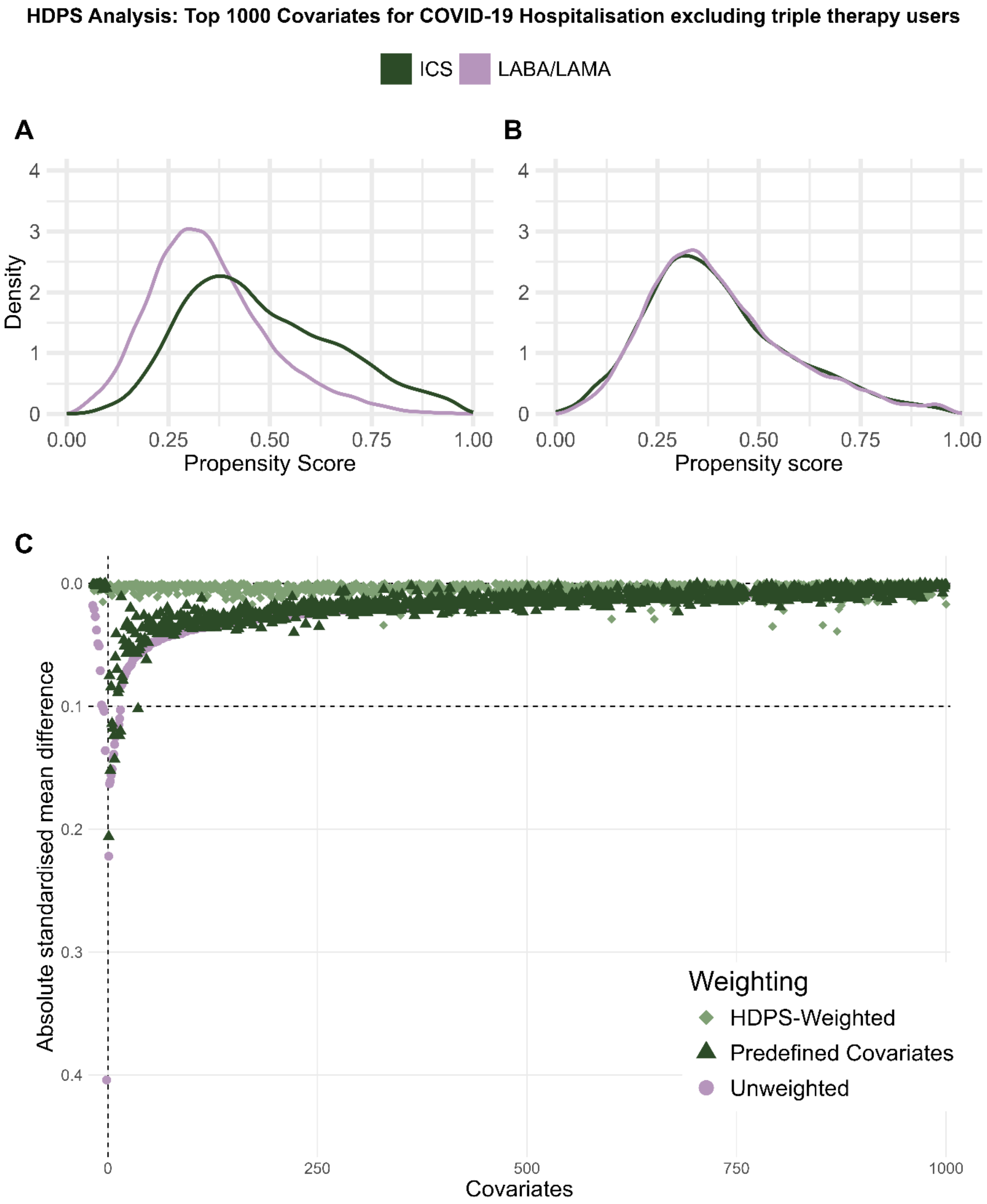

Supplementary Figure 31 Diagnostic plots for high-dimensional propensity score weighted analysis for COVID-19 hospitalisations, excluding triple therapy users, including the top 1000 ranked covariates. A) high-dimensional propensity score (HDPS) distribution, B) weighted HDPS distribution, C) Comparison of absolute standardised differences in the pre-defined and high-dimensional propensity score covariates between unweighted, predefined and HDPS weighted cohort. Points to the left of the x-axis represent predefined covariates, points to the right represent HDPS-identified covariates.

2.5.3. COVID-19 death, including triple therapy users

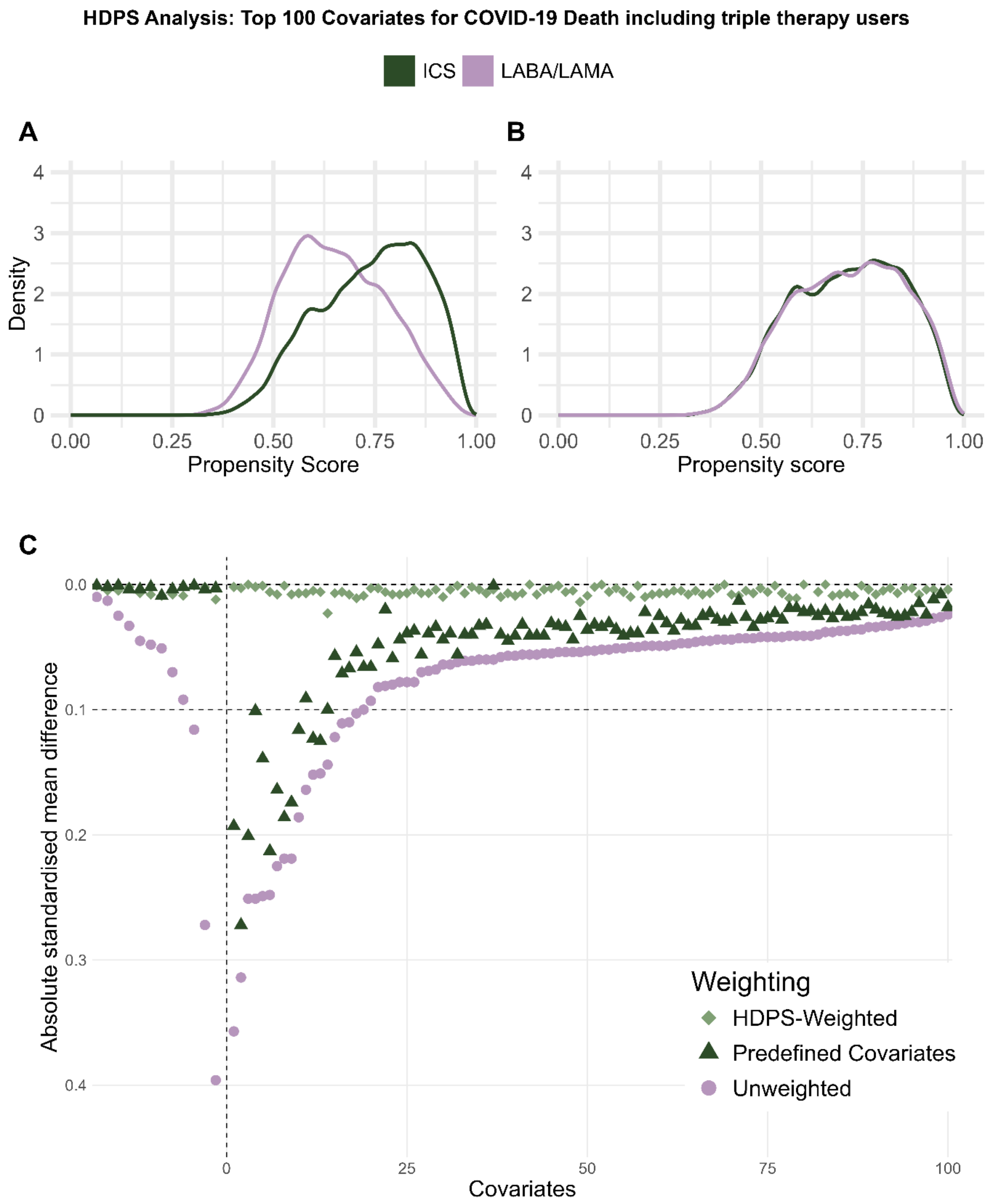

Supplementary Figure 32 Diagnostic plots for high-dimensional propensity score weighted analysis for COVID-19 deaths, including triple therapy users, including the top 100 ranked covariates. A) high-dimensional propensity score (HDPS) distribution, B) weighted HDPS distribution, C) Comparison of absolute standardised differences in the pre-defined and high-dimensional propensity score covariates between unweighted, predefined and HDPS weighted cohort. Points to the left of the x-axis represent predefined covariates, points to the right represent HDPS-identified covariates.

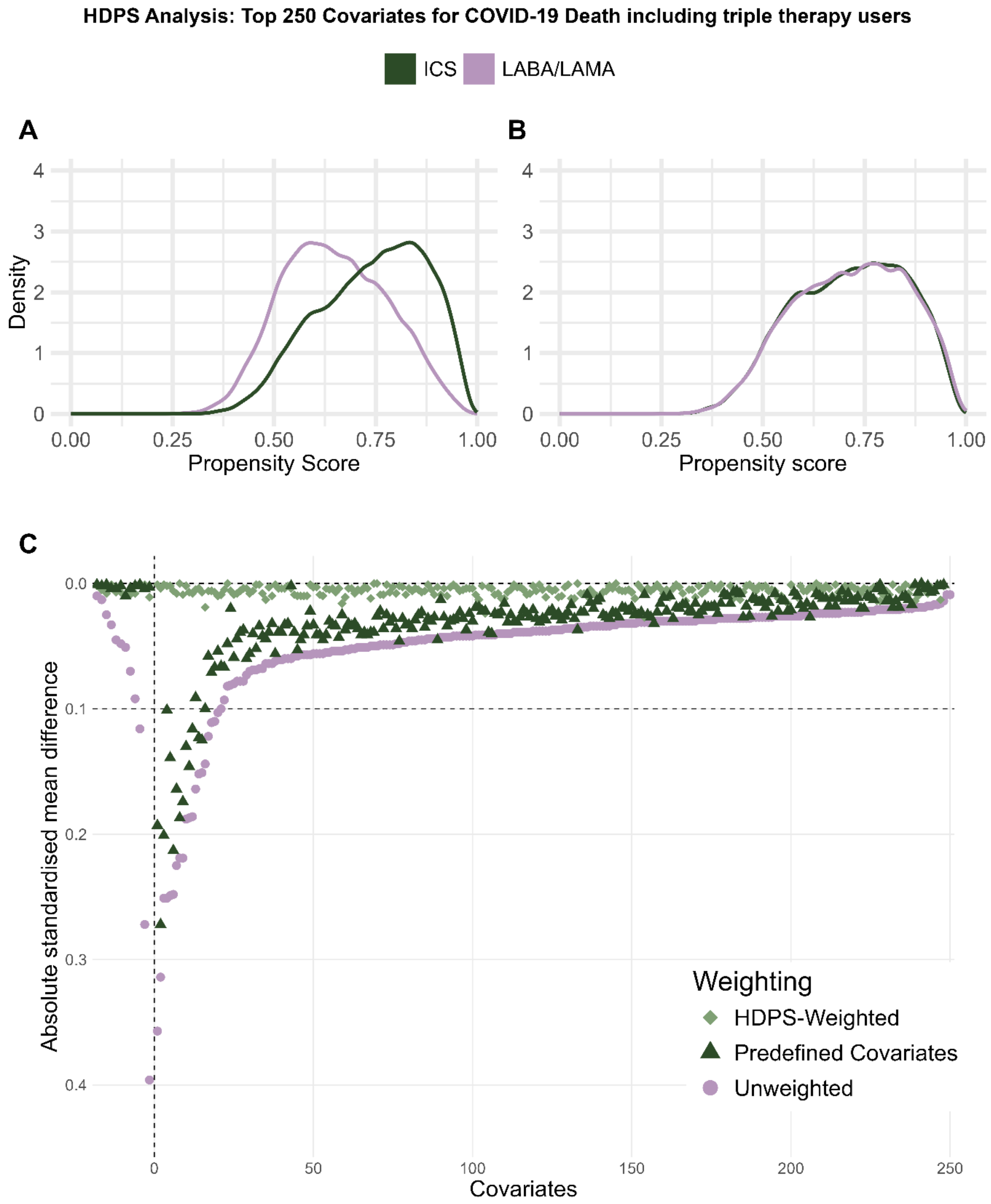

Supplementary Figure 33 Diagnostic plots for high-dimensional propensity score weighted analysis for COVID-19 deaths, including triple therapy users, including the top 250 ranked covariates. A) high-dimensional propensity score (HDPS) distribution, B) weighted HDPS distribution, C) Comparison of absolute standardised differences in the pre-defined and high-dimensional propensity score covariates between unweighted, predefined and HDPS weighted cohort. Points to the left of the x-axis represent predefined covariates, points to the right represent HDPS-identified covariates.

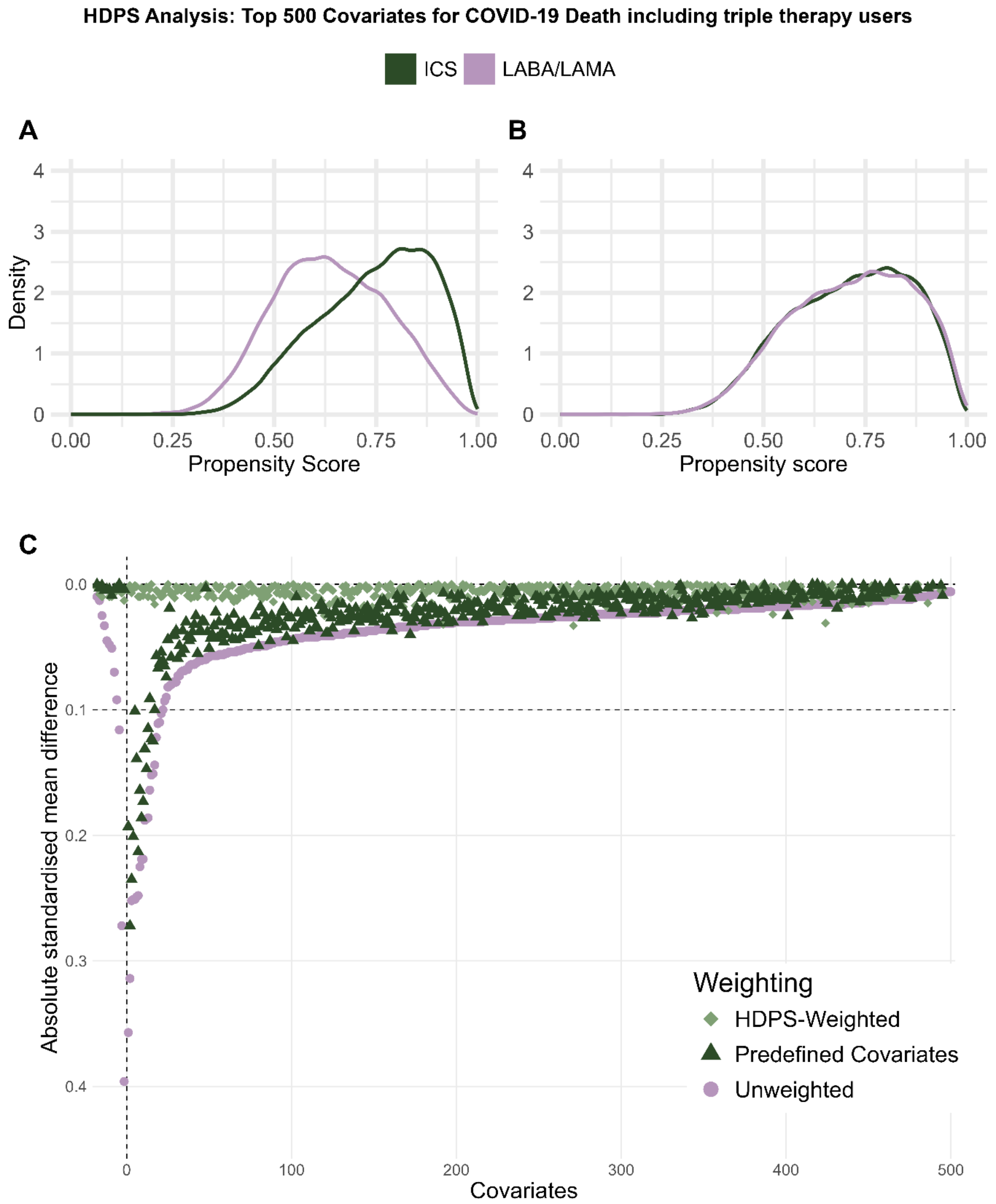

Supplementary Figure 34 Diagnostic plots for high-dimensional propensity score weighted analysis for COVID-19 deaths, including triple therapy users, including the top 500 ranked covariates. A) high-dimensional propensity score (HDPS) distribution, B) weighted HDPS distribution, C) Comparison of absolute standardised differences in the pre-defined and high-dimensional propensity score covariates between unweighted, predefined and HDPS weighted cohort. Points to the left of the x-axis represent predefined covariates, points to the right represent HDPS-identified covariates.

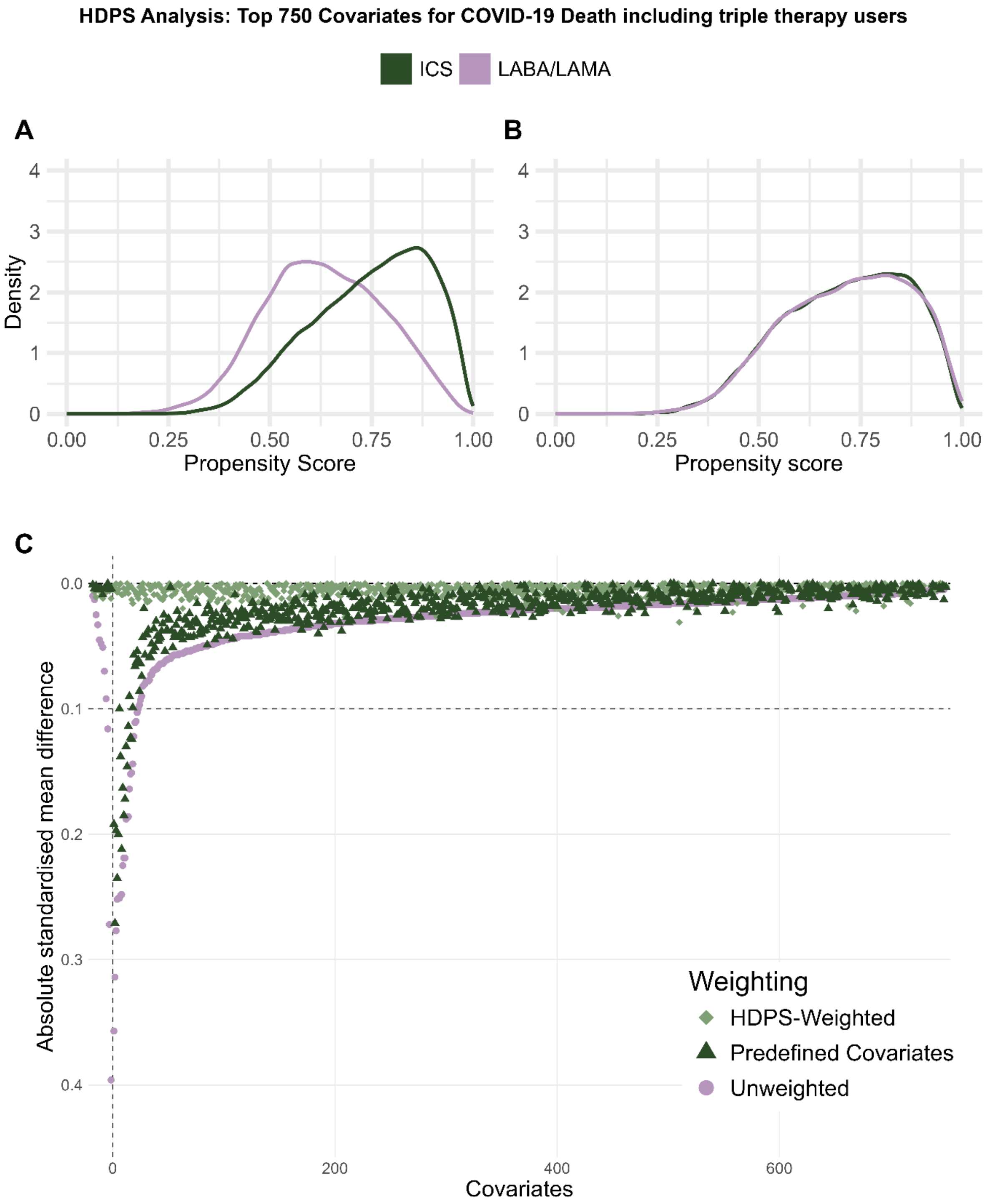

Supplementary Figure 35 Diagnostic plots for high-dimensional propensity score weighted analysis for COVID-19 deaths, including triple therapy users, including the top 750 ranked covariates. A) high-dimensional propensity score (HDPS) distribution, B) weighted HDPS distribution, C) Comparison of absolute standardised differences in the pre-defined and high-dimensional propensity score covariates between unweighted, predefined and HDPS weighted cohort. Points to the left of the x-axis represent predefined covariates, points to the right represent HDPS-identified covariates.

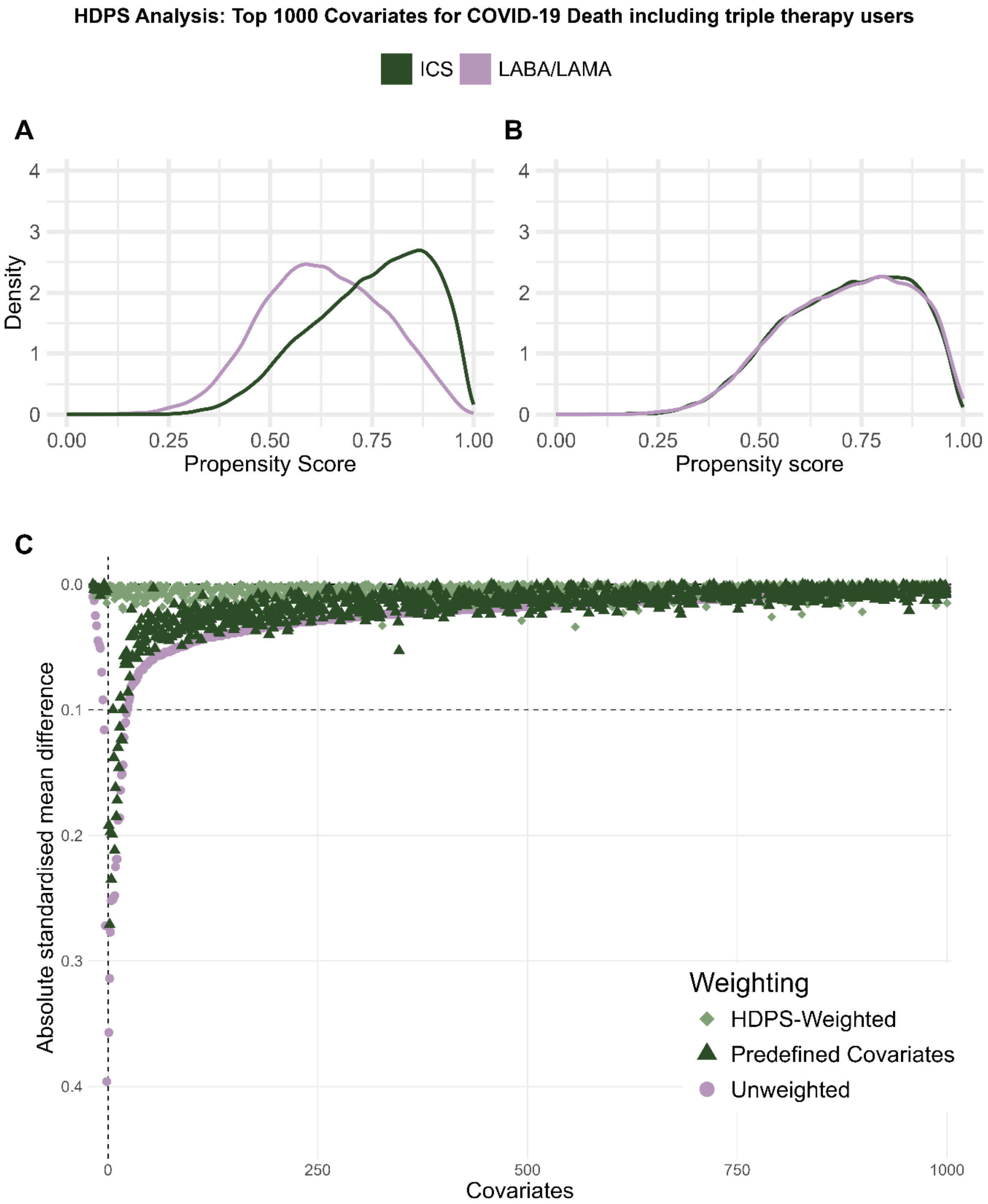

Supplementary Figure 36 Diagnostic plots for high-dimensional propensity score weighted analysis for COVID-19 deaths, including triple therapy users, including the top 1000 ranked covariates. A) high-dimensional propensity score (HDPS) distribution, B) weighted HDPS distribution, C) Comparison of absolute standardised differences in the pre-defined and high-dimensional propensity score covariates between unweighted, predefined and HDPS weighted cohort. Points to the left of the x-axis represent predefined covariates, points to the right represent HDPS-identified covariates.

2.5.4. COVID-19 death, excluding triple therapy users

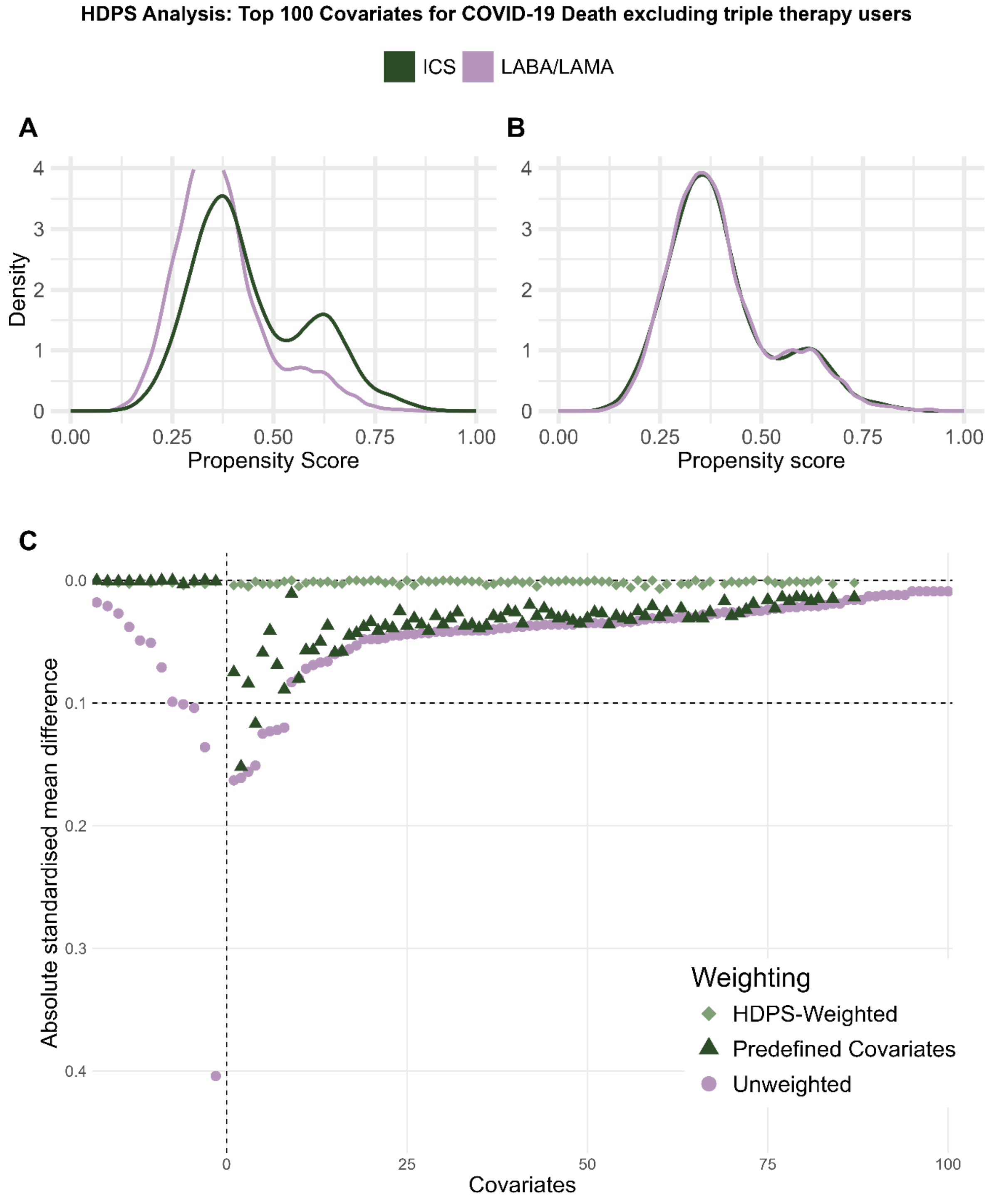

Supplementary Figure 37 Diagnostic plots for high-dimensional propensity score weighted analysis for COVID-19 deaths, excluding triple therapy users, including the top 100 ranked covariates. A) high-dimensional propensity score (HDPS) distribution, B) weighted HDPS distribution, C) Comparison of absolute standardised differences in the pre-defined and high-dimensional propensity score covariates between unweighted, predefined and HDPS weighted cohort. Points to the left of the x-axis represent predefined covariates, points to the right represent HDPS-identified covariates.

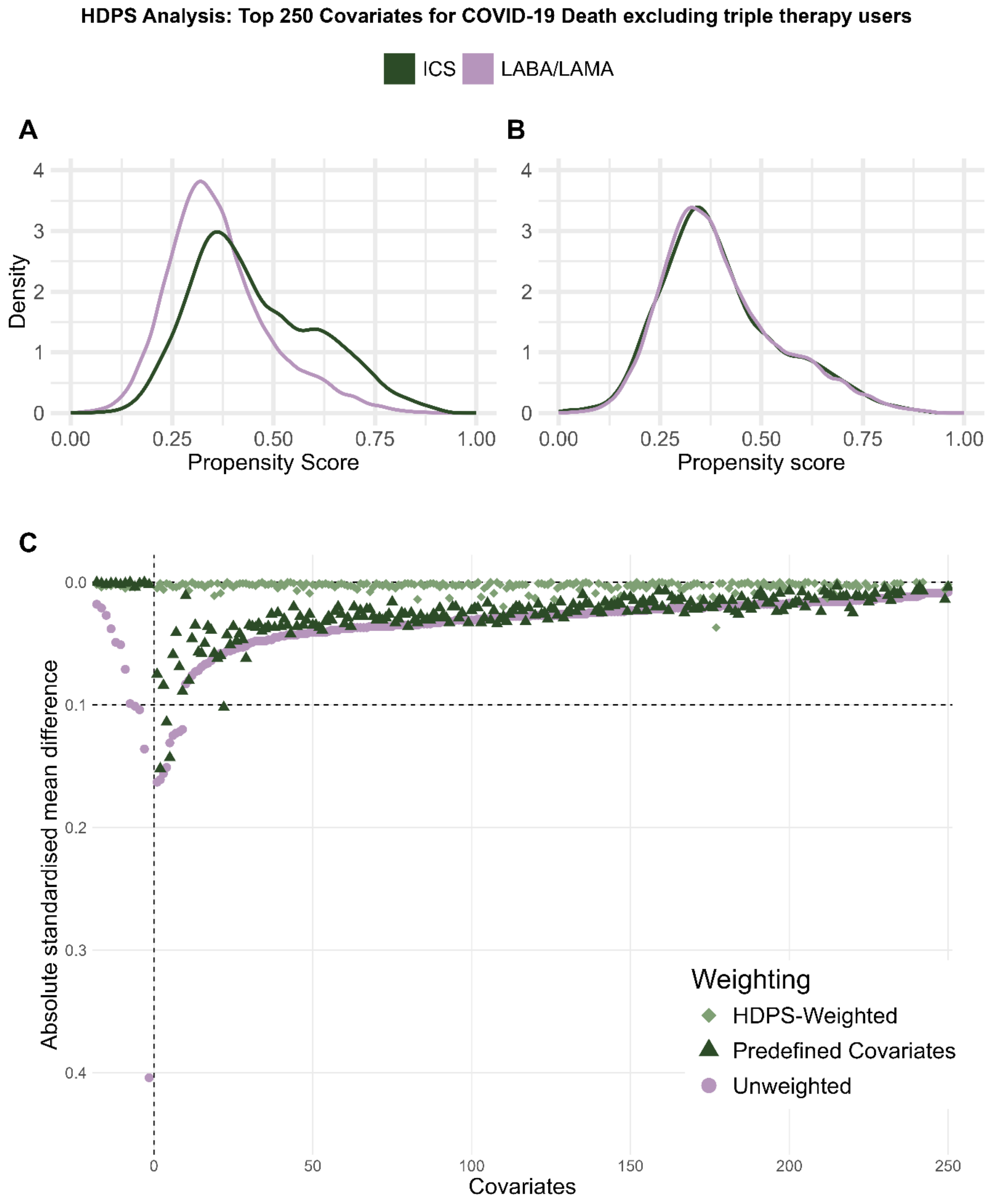

Supplementary Figure 38 Diagnostic plots for high-dimensional propensity score weighted analysis for COVID-19 deaths, excluding triple therapy users, including the top 250 ranked covariates. A) high-dimensional propensity score (HDPS) distribution, B) weighted HDPS distribution, C) Comparison of absolute standardised differences in the pre-defined and high-dimensional propensity score covariates between unweighted, predefined and HDPS weighted cohort. Points to the left of the x-axis represent predefined covariates, points to the right represent HDPS-identified covariates.

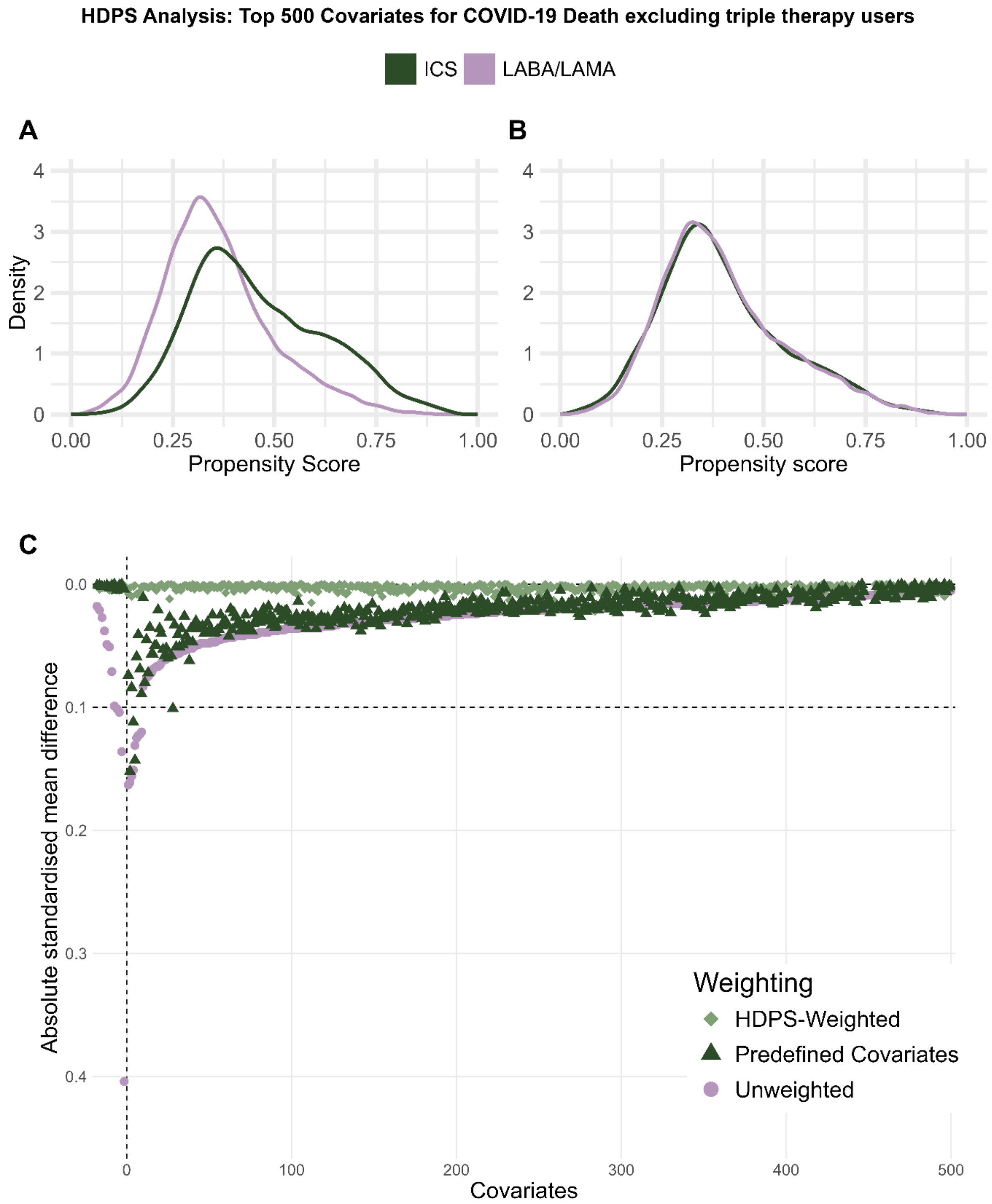

Supplementary Figure 39 Diagnostic plots for high-dimensional propensity score weighted analysis for COVID-19 deaths, excluding triple therapy users, including the top 500 ranked covariates. A) high-dimensional propensity score (HDPS) distribution, B) weighted HDPS distribution, C) Comparison of absolute standardised differences in the pre-defined and high-dimensional propensity score covariates between unweighted, predefined and HDPS weighted cohort. Points to the left of the x-axis represent predefined covariates, points to the right represent HDPS-identified covariates.

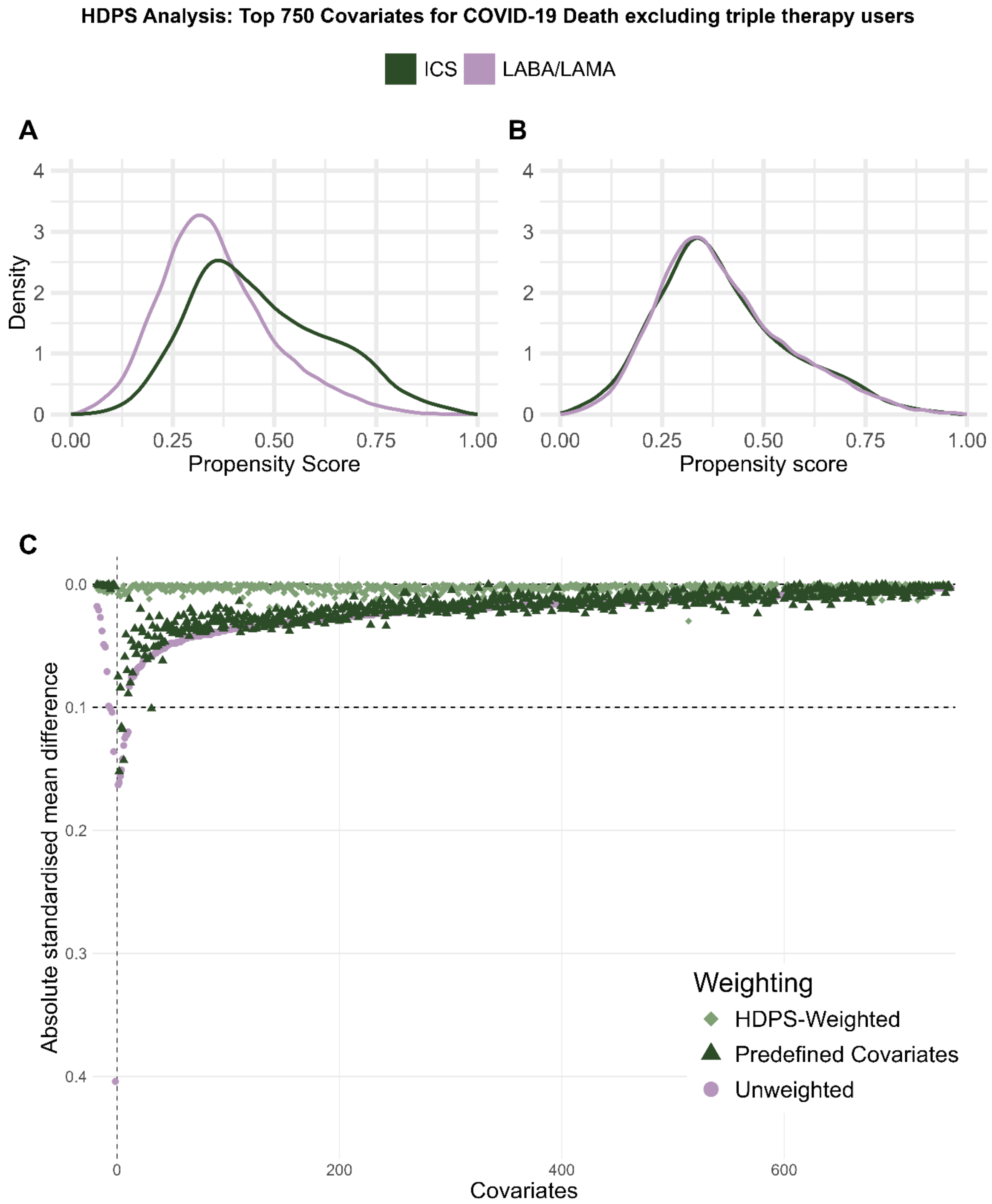

Supplementary Figure 40 Diagnostic plots for high-dimensional propensity score weighted analysis for COVID-19 deaths, excluding triple therapy users, including the top 750 ranked covariates. A) high-dimensional propensity score (HDPS) distribution, B) weighted HDPS distribution, C) Comparison of absolute standardised differences in the pre-defined and high-dimensional propensity score covariates between unweighted, predefined and HDPS weighted cohort. Points to the left of the x-axis represent predefined covariates, points to the right represent HDPS-identified covariates.

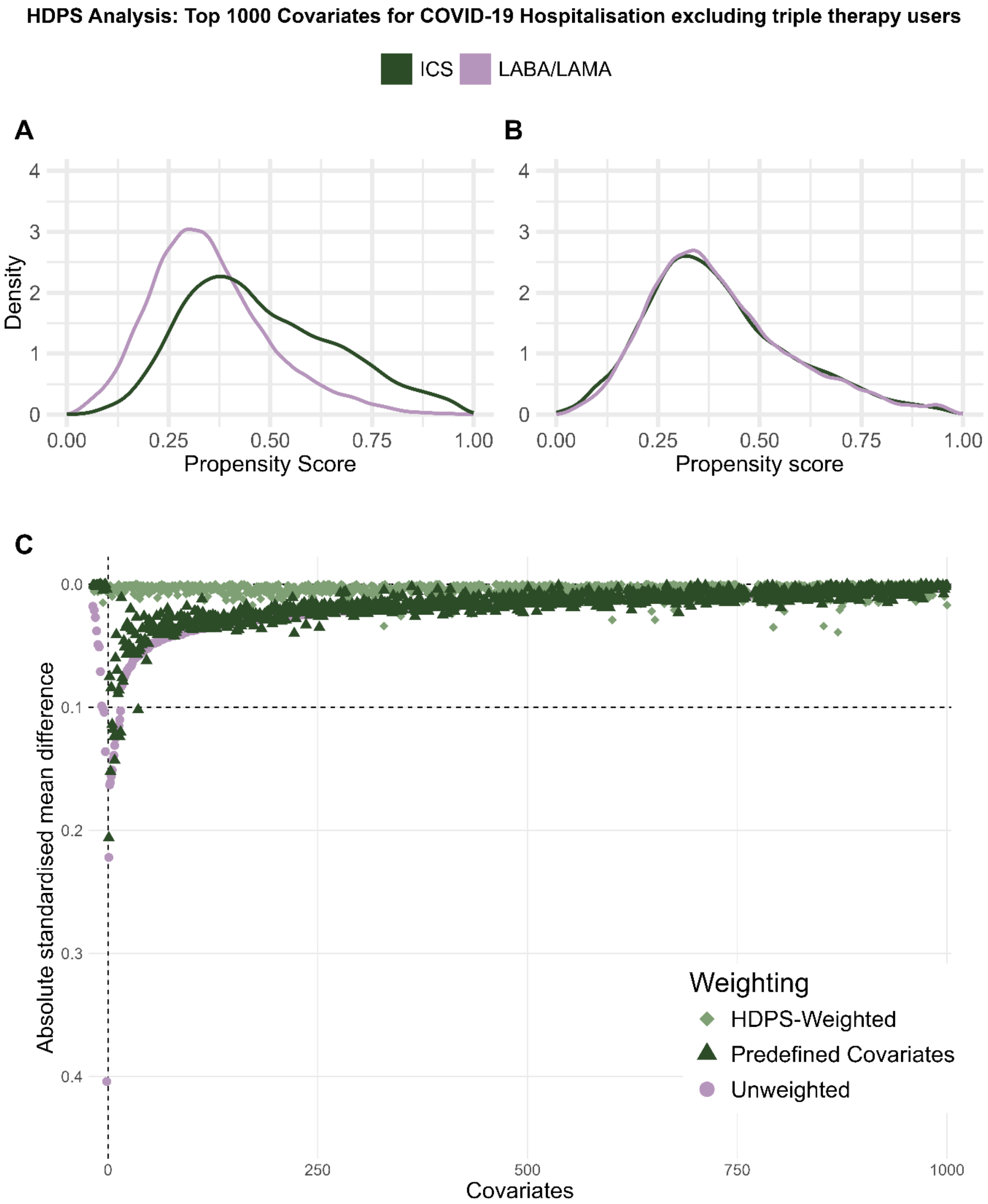

Supplementary Figure 41 Diagnostic plots for high-dimensional propensity score weighted analysis for COVID-19 deaths, excluding triple therapy users, including the top 1000 ranked covariates. A) high-dimensional propensity score (HDPS) distribution, B) weighted HDPS distribution, C) Comparison of absolute standardised differences in the pre-defined and high-dimensional propensity score covariates between unweighted, predefined and HDPS weighted cohort. Points to the left of the x-axis represent predefined covariates, points to the right represent HDPS-identified covariates.

2.6. Cox proportional hazards models

2.6.1. Kaplan-Meier plots

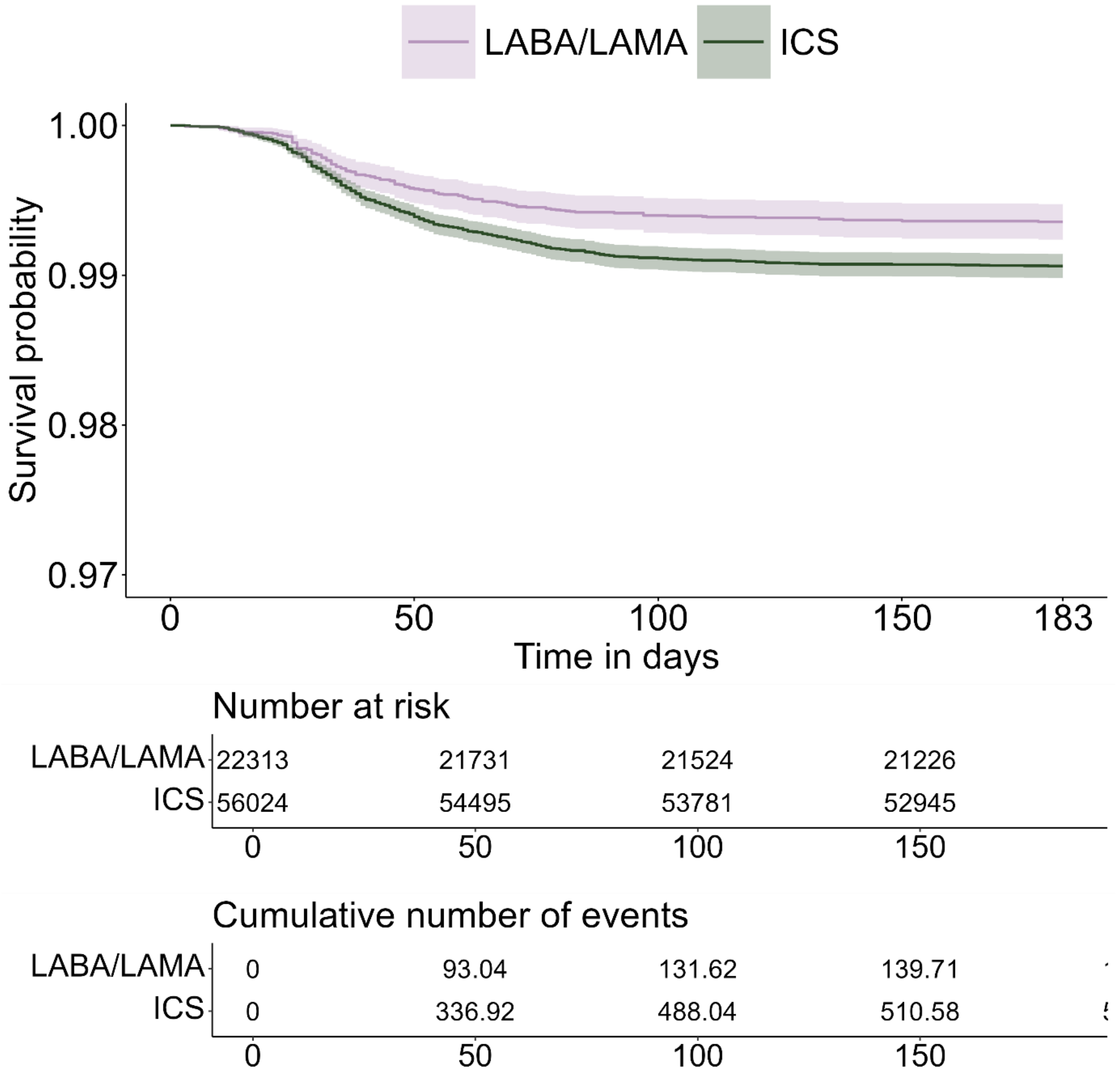

Supplementary Figure 42 Kaplan-Meier curves for COVID-19 hospitalisation, including triple therapy users, weighted using prespecified covariates

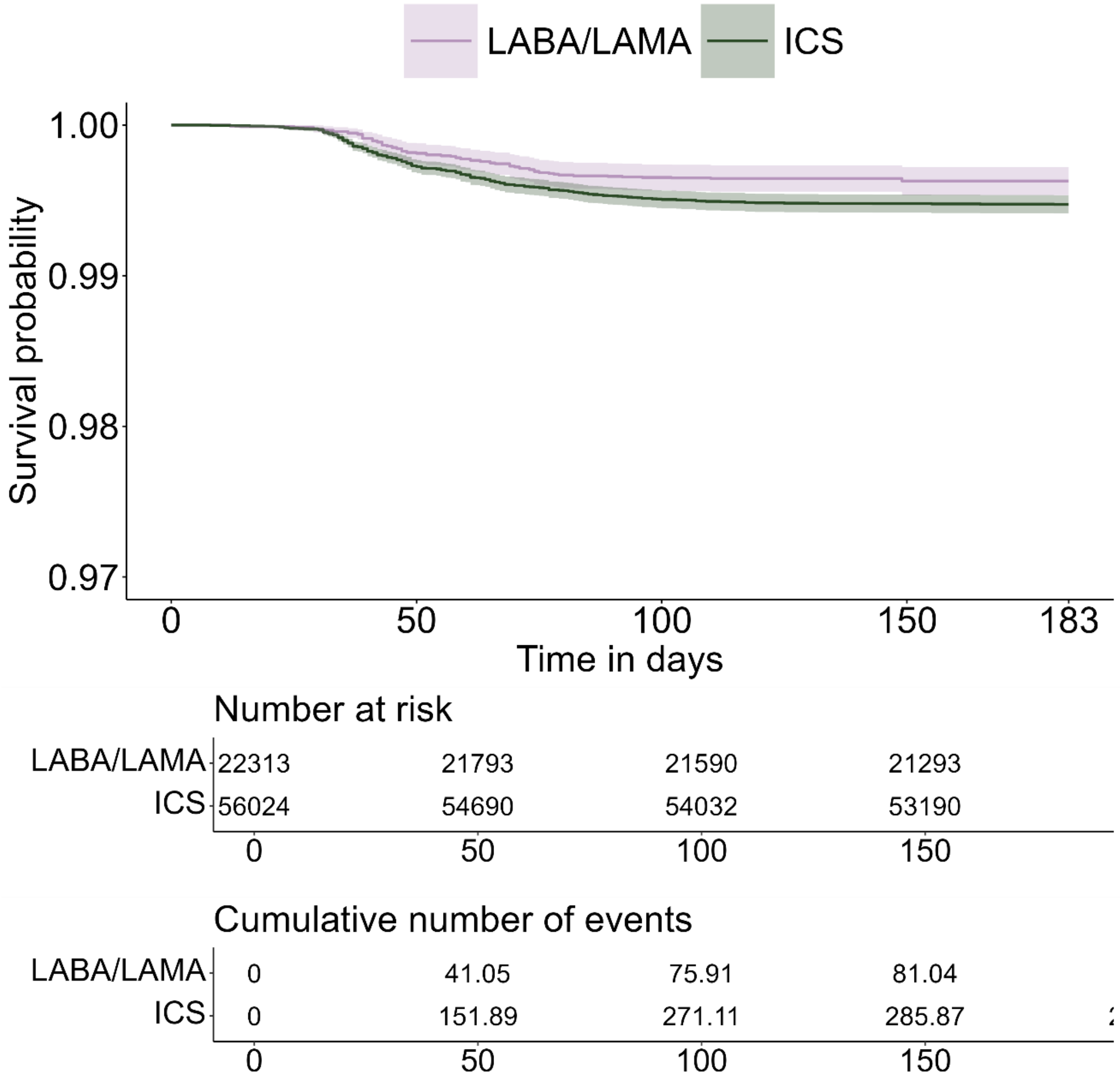

Supplementary Figure 43 Kaplan-Meier curves for COVID-19 death, including triple therapy users, weighted using prespecified covariates

2.7. Logistic regression models

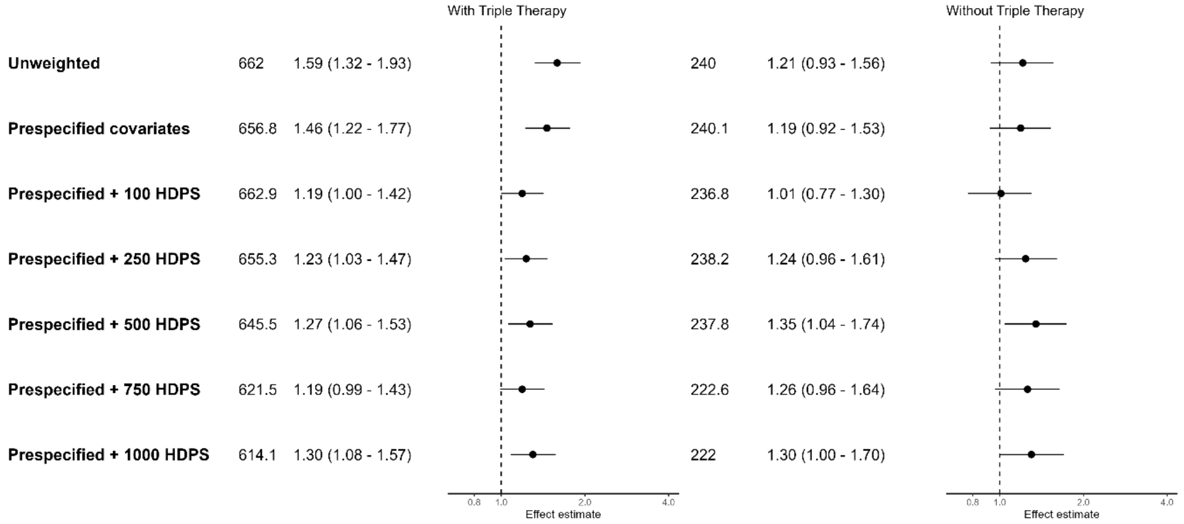

Supplementary Figure 44 Forest plot of odds ratios and 95% confidence intervals for COVID-19 hospitalisations, comparing ICS/LABA (+/- LAMA) users to LABA/LAMA users. Effect estimates >1 indicate an increased risk in the ICS group.

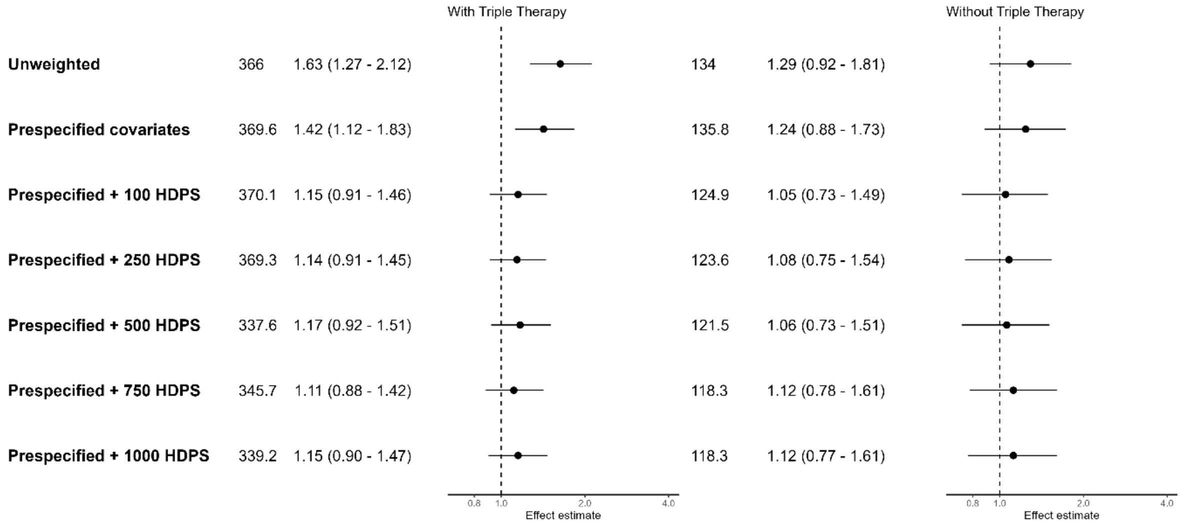

Supplementary Figure 45 Forest plot of odds ratios and 95% confidence intervals for COVID-19 deaths, comparing ICS/LABA (+/- LAMA) users to LABA/LAMA users. Effect estimates >1 indicate an increased risk in the ICS group.

2.8. Risk differences

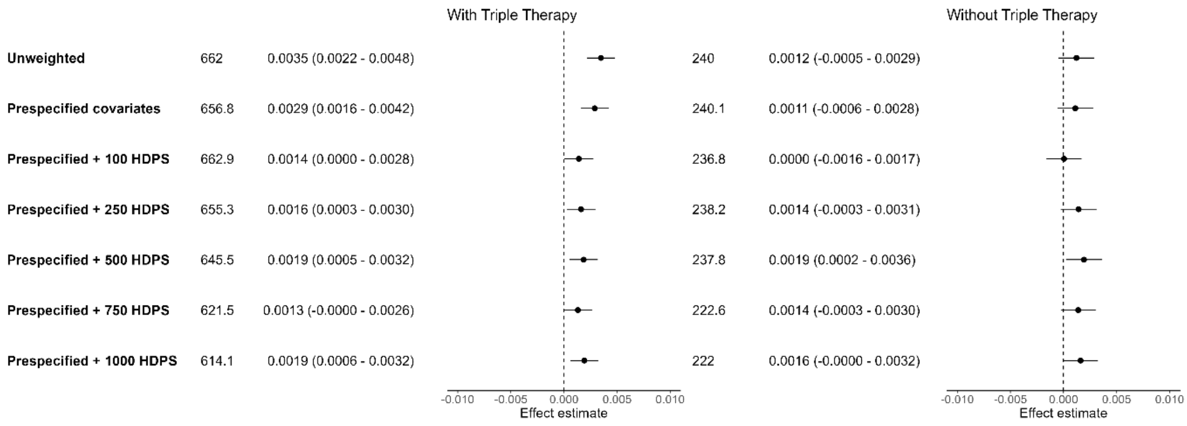

Supplementary Figure 46 Risk differences for the outcome COVID-19 hospitalisation

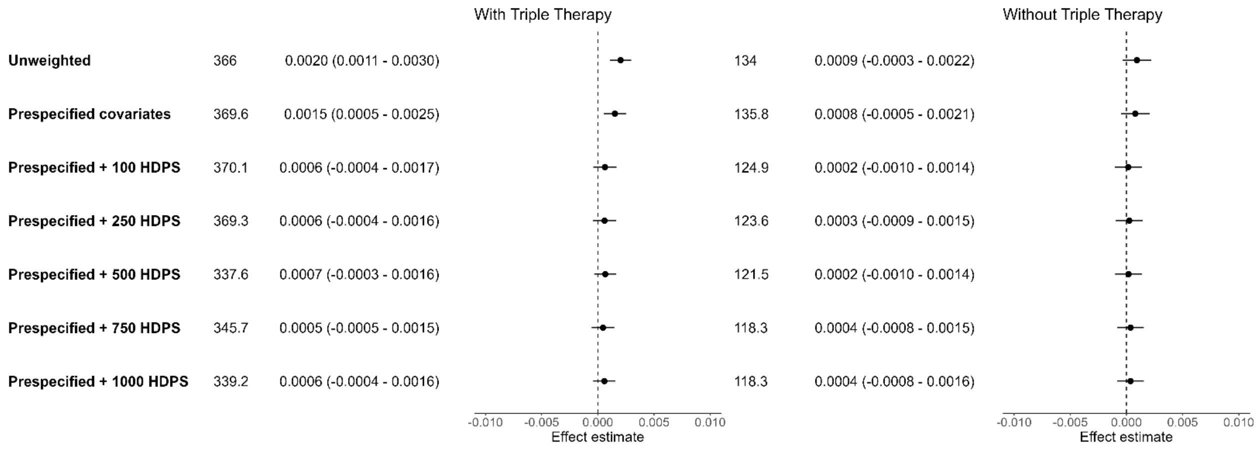

Supplementary Figure 47 Risk differences for the outcome COVID-19 death

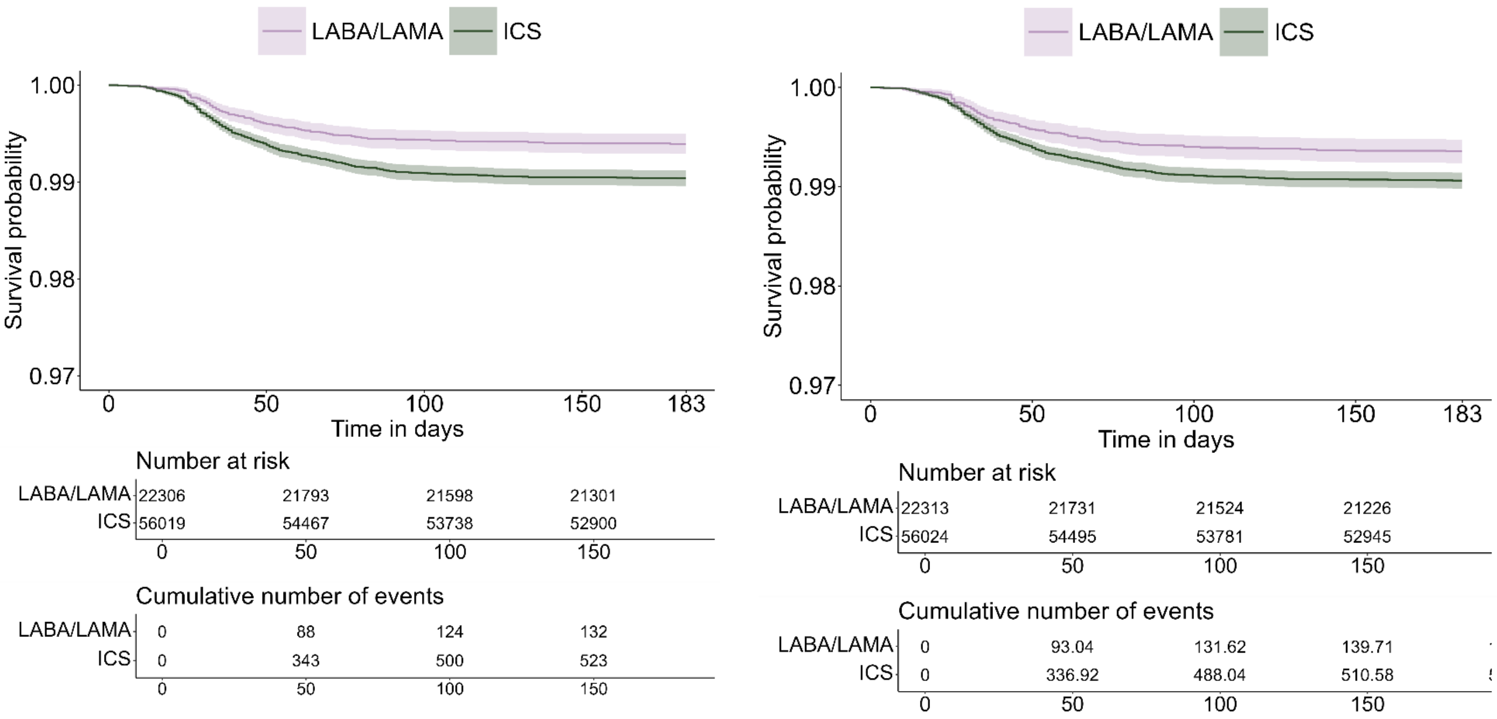

Supplementary Figure 48 Unadjusted (left) and adjusted (right) survival curves for COVID-19 hospitalisation, including triple therapy users. The adjusted curves account for prespecified covariates

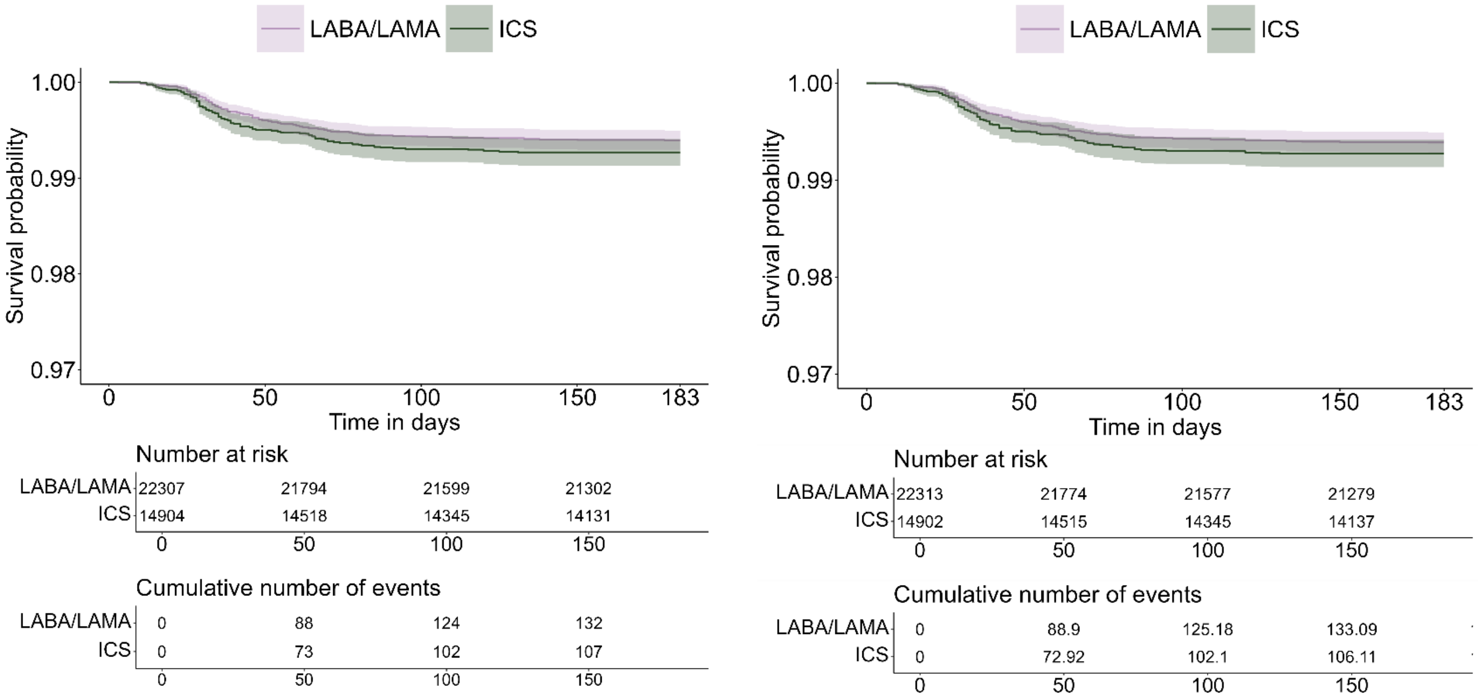

Supplementary Figure 49 Unadjusted (left) and adjusted (right) survival curves for COVID-19 hospitalisation, excluding triple therapy users. The adjusted curves account for prespecified covariates.

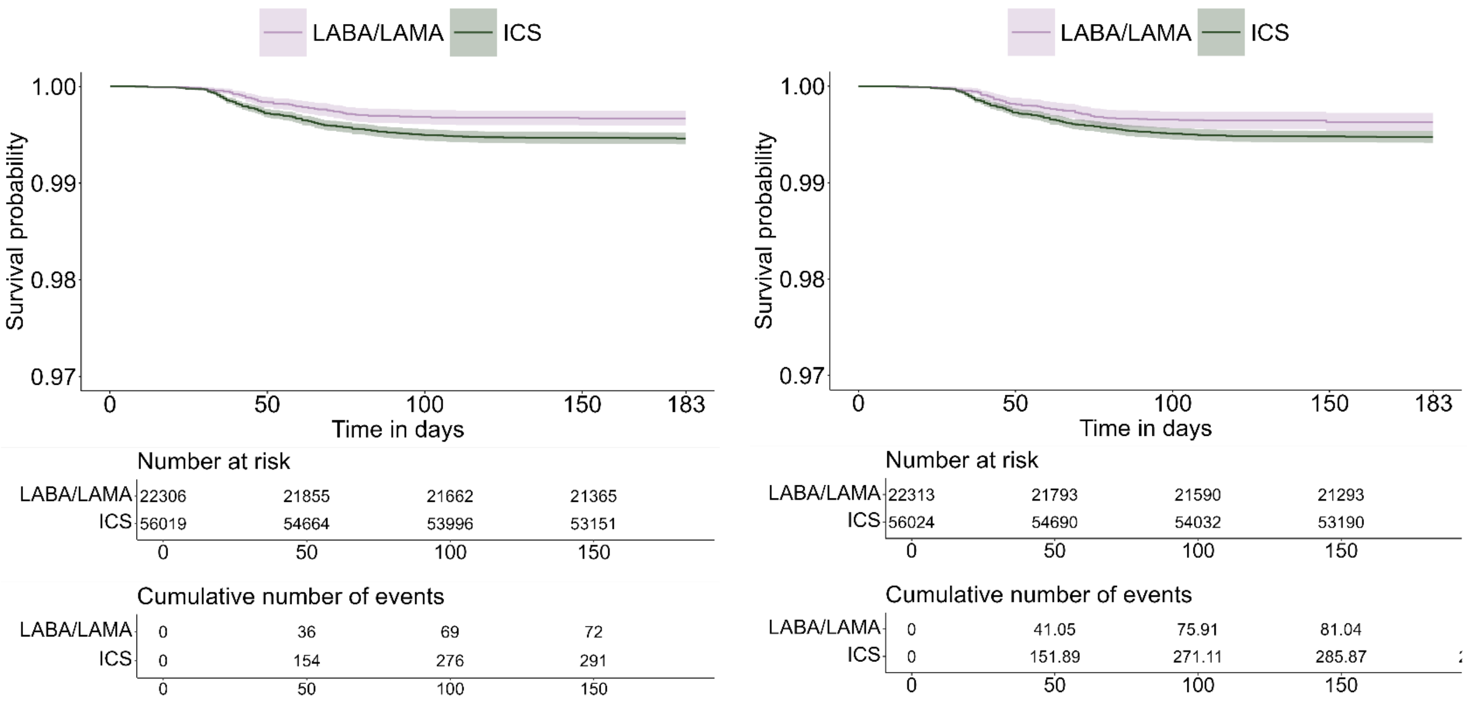

Supplementary Figure 50 Unadjusted (left) and adjusted (right) survival curves for COVID-19 death, including triple therapy users. The adjusted curves account for prespecified covariates.

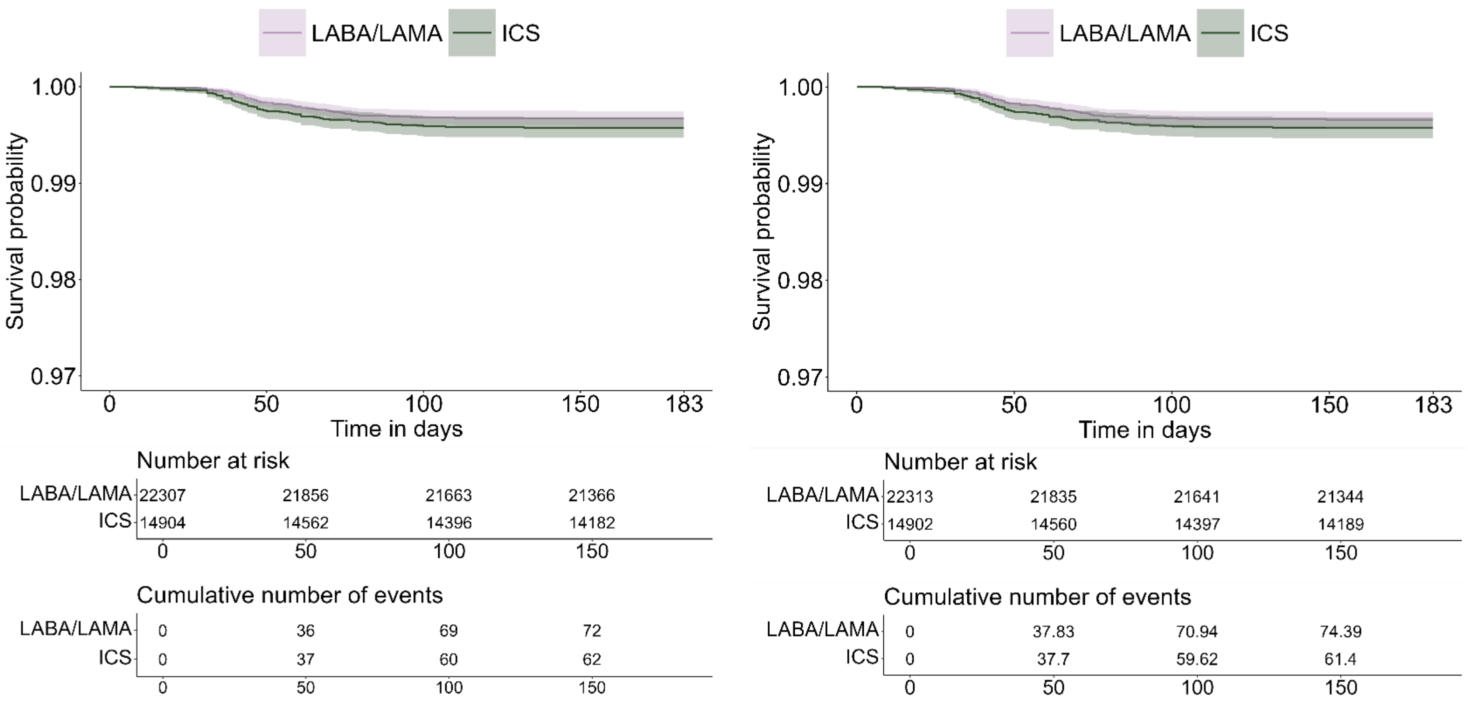

Supplementary Figure 51 Unadjusted (left) and adjusted (right) survival curves for COVID-19 death, excluding triple therapy users. The adjusted curves account for prespecified covariates
